# Single‐cell transcriptomics stratifies organoid models of metabolic dysfunction‐associated steatotic liver disease

**DOI:** 10.15252/embj.2023113898

**Published:** 2023-11-14

**Authors:** Anja Hess, Stefan D Gentile, Amel Ben Saad, Raza‐Ur Rahman, Tim Habboub, Daniel S Pratt, Alan C Mullen

**Affiliations:** ^1^ Division of Gastroenterology, Massachusetts General Hospital Harvard Medical School Boston MA USA; ^2^ Klarman Cell Observatory Broad Institute of MIT and Harvard Cambridge MA USA; ^3^ Autoimmune and Cholestatic Liver Center Massachusetts General Hospital Boston MA USA; ^4^ Center for the Study of Inflammatory Bowel Disease Massachusetts General Hospital Boston MA USA; ^5^ Harvard Stem Cell Institute Cambridge MA USA; ^6^ Present address: Department of Genome Regulation Max Planck Institute for Molecular Genetics Berlin Germany; ^7^ Present address: University of Massachusetts Chan Medical School Worcester MA USA

**Keywords:** fibrosis, liver organoids, MASLD, NAFLD, single‐cell RNA‐sequencing, Metabolism, Methods & Resources, Molecular Biology of Disease

## Abstract

Metabolic dysfunction‐associated steatotic liver disease (MASLD) is a growing cause of morbidity with limited treatment options. Thus, accurate *in vitro* systems to test new therapies are indispensable. While recently, human liver organoid models have emerged to assess steatotic liver disease, a systematic evaluation of their translational potential is still missing. Here, we evaluated human liver organoid models of MASLD, comparatively testing disease induction in three conditions: oleic acid, palmitic acid, and TGF‐β1. Through single‐cell analyses, we find that all three models induce inflammatory signatures, but only TGF‐β1 promotes collagen production, fibrosis, and hepatic stellate cell expansion. In striking contrast, oleic acid ameliorates fibrotic signatures and reduces the hepatic stellate cell population. Linking data from each model to gene expression signatures associated with MASLD disease progression further demonstrates that palmitic acid and TGF‐β1 more robustly model inflammation and fibrosis. Our findings highlight the importance of stratifying MASLD organoid models by signatures of clinical disease progression, provide a single‐cell reference to benchmark future organoid injury models, and allow us to study evolving steatohepatitis, fibrosis, and HSC susceptibility to injury in a dynamic, multi‐lineage human *in vitro* system.

## Graphical abstract

Comprehensive dissection of fatty liver disease in human organoids uncovers unexpected phenotype heterogeneity triggered by specific fatty acids.

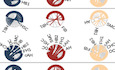

## Synopsis

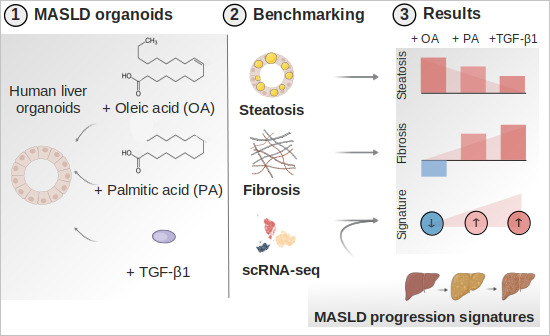

Metabolic dysfunction‐associated steatotic liver disease (MASLD, formerly NAFLD) is a major cause of chronic hepatic injury and failure, however the details of its induction remain unclear. This benchmark resource reports *in vitro* dissection of fatty liver disease in human organoids uncovering distinct disease phenotypes triggered by specific lipids.

- Human pluripotent stem cell‐derived liver organoids serve as models for injury related to MASLD.
- Palmitic acid induces inflammatory signatures while TGF‐β1 signaling induces inflammatory and fibrotic signatures.
- Oleic acid reduces hepatic stellate cell numbers and suppresses collagen production.
- Gene expression predicting MASLD severity increases from palmitic acid to TGF‐β1 treatment.

## Introduction

Chronic liver injury promotes sustained inflammation, leading to liver fibrosis, which can progress to cirrhosis (Loomba *et al*, 2021), a major cause of morbidity and mortality worldwide (Sepanlou *et al*, 2020). Metabolic dysfunction‐associated steatotic liver disease (MASLD, also known as non‐alcoholic fatty liver disease or NAFLD) (Rinella *et al*, 2023) is tightly linked to obesity and metabolic syndrome (Anstee *et al*, 2019). It is among the most common causes of chronic liver disease (Sepanlou *et al*, 2020) and the most rapidly increasing indication for liver transplantation in the United States (Younossi *et al*, 2021). While the majority of MASLD cases that lead to end‐stage liver disease progress from simple steatosis (fatty liver) to metabolic dysfunction‐associated steatohepatitis (MASH, inflammation due to steatosis) and then fibrosis (Kisseleva & Brenner, 2021; Huby & Gautier, 2022), it is important to acknowledge that only approximately 30% of individuals with liver steatosis develop MASH (Younossi *et al*, 2016). While the progression from MASH to fibrosis is well documented, there are currently few treatment options to disrupt this process other than weight loss (Ferguson & Finck, 2021). More broadly, there are no approved treatments available that target common inflammatory or fibrotic pathways arising from chronic liver injury, which could prevent the progression of MASH or other chronic liver diseases (Ferguson & Finck, 2021). Thus, the development and evaluation of *in vitro* human cell models of liver inflammation and fibrosis are critical for creating and testing new approaches to prevent liver failure.

A key prerequisite for MASLD‐associated chronic liver injury is the continued exposure to excess fatty acids (Nehra *et al*, 2001; Donnelly *et al*, 2005; Neuschwander‐Tetri, 2010). The free fatty acids (FFAs) oleic acid (OA) and palmitic acid (PA) accumulate in MASLD and thus are widely used to model MASLD *in vitro* (Müller & Sturla, 2019; Ramli *et al*, 2020). OA, a monounsaturated fatty acid (MUFA), induces hepatocyte steatosis but has also been reported to exert protective effects against MASLD (Chen *et al*, 2018; Zeng *et al*, 2020). PA, a saturated fatty acid (SFA), promotes hepatocyte apoptosis (Akazawa *et al*, 2010; Miura *et al*, 2013).

FFA‐induced hepatocyte damage activates pro‐inflammatory signaling pathways in resident liver cells (Anstee *et al*, 2019). This leads to the secretion of cytokines such as tumor necrosis factor (TNF) (Anstee *et al*, 2019; Kisseleva & Brenner, 2021), activating resident liver macrophages (Kupffer cells) (Sunami *et al*, 2012). Kupffer cells and recruited immune cells (Zigmond *et al*, 2014) are a source of transforming growth factor beta (TGF‐β) (Lodyga *et al*, 2019), which activates hepatic stellate cells (HSCs), and promotes their transdifferentiation towards myofibroblasts (Tsuchida & Friedman, 2017). These HSC myofibroblasts produce extracellular matrix (ECM, predominantly collagen type I and III) that accumulates to form the fibrotic scar (Tsuchida & Friedman, 2017). Hallmarks of fibrosis include the up‐regulation of transcripts *COL1A1*, *COL3A1*, *TIMP1*, *TNFS* members, and the receptor‐ligand‐pair *PDGFB*/*PDGFR* (Tsuchida & Friedman, 2017; Ramachandran *et al*, 2019). Additionally, chronic liver injury is also associated with the expansion of cells with ductular characteristics, which can originate from cholangiocytes, hepatic progenitors, and hepatocytes to replace injured hepatocytes (Español‐Suñer *et al*, 2012; Raven *et al*, 2017; Deng *et al*, 2018; Sato *et al*, 2019).

Recently, the generation of multi‐lineage hepatic organoids from human pluripotent stem cells (hPSCs) has emerged (Ouchi *et al*, 2019; Wu *et al*, 2019; Sekine *et al*, 2020; Guan *et al*, 2021; Shinozawa *et al*, 2021). These systems contain cells of at least endodermal and mesodermal identity (e.g., hepatocyte‐, cholangiocyte‐, and HSC‐like cells) and are also referred to as multi‐tissue organoids (Marsee *et al*, 2021). Some multi‐tissue organoids, herein referred to as human liver organoids (HLOs) (Ouchi *et al*, 2019), have been shown to recapitulate aspects of liver inflammation and fibrosis (Ouchi *et al*, 2019; Guan *et al*, 2021), and thus are promising *in vitro* models for MASLD. However, to validate their translational potential, a comparative evaluation of such systems is urgently needed. Single‐cell RNA sequencing (scRNA‐seq) has emerged as a key tool to identify disease signatures in human organoids at high resolution (Bock *et al*, 2021). To our knowledge, there are currently no studies comparing the hepatic injury type and MASLD severity induced by different agents in HLO models at single‐cell resolution.

Here, we develop a structured approach to evaluate MASLD models in an HLO system and provide a reference of ~ 100,000 single‐cell transcriptomes reflecting the HLO injury landscape. We examine OA, PA, and TGF‐β1 for their potential to induce collagen production and select the optimal 3D culture environment for this purpose. Next, we apply functional and 10× scRNA‐seq transcriptomic analysis to evaluate the induction of inflammatory and fibrotic signatures. Finally, we apply clinical MASLD gene signatures to score the severity of the generated HLO injury. This approach allows us to stratify *in vitro* MASLD models by their alignment to disease progression in patients and identifies PA as the more robust FFA model of MASH.

## Results

### Human liver organoids recapitulate the transcriptional landscape of major cell types in the adult human liver

**Figure 1:**
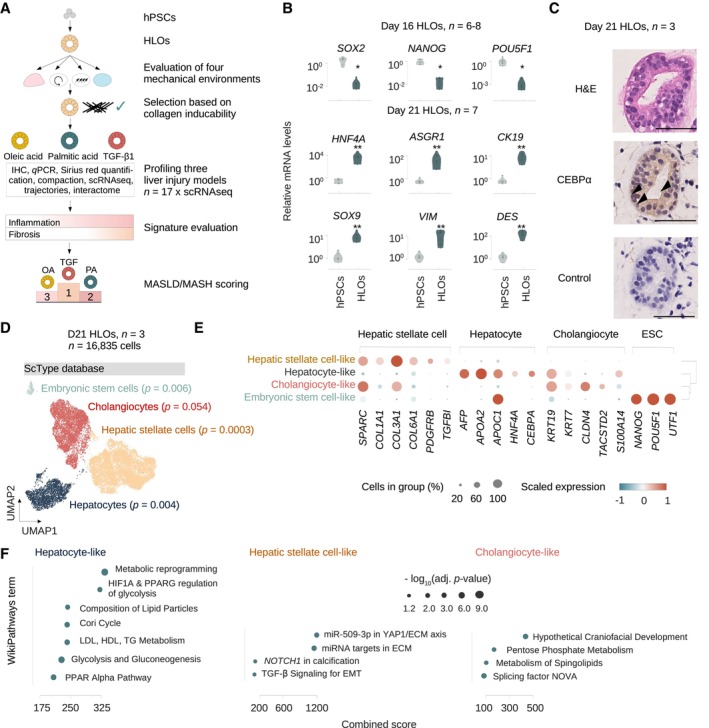
Human liver organoids recapitulate the transcriptional landscape of major cell types in the adult human liver AOverview of the experimental design. Human pluripotent stem cells (hPSCs) are differentiated into human liver organoids (HLOs) and cultured in four different mechancial environments. The environment suitable for collagen induction is selected, and three injury scenarios (OA, PA, TGF‐β1) are evaluated for their potential to model inflammation and fibrosis by functional and transcriptomic readouts, with a total of 17 HLO samples being subjected to 10× single‐cell RNA sequencing (scRNA‐seq). Transcriptomic signatures for inflammation and fibrosis are derived and evaluated. Finally, a MASLD/MASH severity score is applied, allowing for the hierarchical ordering of HLO injury models by their potential to model MASLD disease progression.BExpression of pluripotency genes is reduced with HLO differentiation, while liver markers are induced. Relative gene expression is measured by quantitative reverse transcriptase (qRT) PCR and normalized by housekeeping gene *ACTB* and displayed relative to hPSC controls ($2^{-\Delta \Delta C_{t}}$). Comparison between hPSCs and HLOs from the same experiment were performed at day 16 or 21 as indicated. *N* ≥ 6 individual experiments (batches) as indicated by circles. Horizontal lines in violin plots represent quartiles, Kernel density estimates are trimmed to the range of the observed data. Mann–Whitney‐*U*‐statistics (two‐tailed) hPSCs vs. HLOs: *P*‐values^day 16 HLOs^ = 0.0024, Bonferroni‐adjusted level of significance: 0.0125 (*); *P*‐values^day 21 HLOs^ = 0.0022, Bonferroni‐adjusted level of significance: 0.0071 (**).CHLOs on day 21 show an organized, luminal structure and express human liver proteins. Representative immunohistochemistry images of HLOs stained in hematoxylin & eosin (H&E), CEBPa, and negative control with secondary antibody on day 21. Arrowheads indicate sites of nuclear protein staining. Scale bars, 50 μm. *N* = 3 individual experiments (batches).DscRNA‐seq analysis of day 21 HLOs identifies the major cell types of the human liver as annotated by scType (Ianevski *et al*, 2022) database marker genes. UMAP plots showing cells from 10× scRNA‐seq in day 21 HLOs (*n* = 3 technical replicates of one experimental batch). Cell type annotations by EnrichR over‐representation analysis (Kuleshov *et al*, 2016; Fang *et al*, 2022) with scType (Ianevski *et al*, 2022), adjusted *P*‐value as indicated in the figure.EDotplot showing the expression of canonical cell‐type‐specific marker genes expected in the human liver across clusters. Cell types defined by single‐cell data are displayed on the *y*‐axis, and marker genes (bottom) are sorted by cell type (top). The fraction of cells expressing a gene is indicated by the size of the circle, and the scaled mean expression of a gene is indicated by color. Hierarchical clustering is represented by the dendrogram on the right.FTop enriched WikiPathways (Martens *et al*, 2021) terms for the three major cell types in day 21 HLOs. Terms were sorted by combined score and have been shortened for readability. Dot sizes correspond to the negative decadic logarithm of the adjusted *P*‐value, dot color represents the Odds ratio for each term. Source data are available online for this figure.

We first differentiated hPSCs into HLOs as described (Ouchi *et al*, 2019; Thompson & Takebe, 2020) (Fig 1A). We confirmed loss of the canonical pluripotency genes *SOX2*, *NANOG*, and *POU5F1* by day 16 of differentiation and induction of the human liver genes *ASGR1* and *HNF4A* (hepatocyte markers, Aizarani *et al*, 2019), *KRT19*/*CK19* and *SOX9* (cholangiocyte markers, MacParland *et al*, 2018) as well as *VIM* and *DES* (HSC markers, Payen *et al*, 2021) in day 21 HLOs (Fig 1B). To evaluate HLO identity at the protein and histologic level, we performed hematoxylin and eosin (H&E) staining and immunohistochemistry (IHC) and found that HLOs at day 21 display an organized, sphero‐luminal structure, and multiple cells express the nuclear hepatocyte marker CEBPα (Fig 1C, Appendix Fig S1). These results are in agreement with previous observations on the emergence of distinct liver cell types from at least two different lineages in HLOs after day 20 (Ouchi *et al*, 2019).

To further investigate the cellular composition of HLOs, we performed 10× scRNA‐seq on day 21 and analyzed a total of 16,835 cells after quality control (QC) (Appendix Fig S2a, Materials and Methods). Annotation of HLO populations can create difficulties since differentiating systems may contain transient cell states and retain premature features (Bhaduri *et al*, 2020). To address these challenges, we initially evaluated three different annotation strategies (Clarke *et al*, 2021). We first annotated cell clusters based on marker genes from the literature for fetal and adult liver cell types (Appendix Fig S2b, Dataset EV1), rendering four main clusters of hepatocyte‐like, HSC‐like, cholangiocyte‐like cells, and a small fraction of embryonic stem cell (ESC)‐like cells (Appendix Fig S2c). In a second approach, we evaluated CellTypist (Domínguez Conde *et al*, 2022), a logistic regression‐based classifier to compare the major HLO clusters to scRNA‐seq data from the developing human liver (Wesley *et al*, 2022) (Appendix Fig S2d). The CellTypist annotations are consistent with the literature approach, except the embryonic stem cell‐like population is attributed to the hepatoblast‐like cluster, potentially reflecting the partial expression of pluripotency genes such as *NANOG* in hepatoblasts (Wesley *et al*, 2022). We then evaluated ScType, a marker gene database validated on human adult liver scRNA‐seq data (Ianevski *et al*, 2022) (Fig 1D, Dataset EV3). Analysis with the scType database showed similar results to the previous approaches, and labeled a small embryonic stem cell‐like population in agreement with the literature‐based approach.

Overall, the attributions of hepatocyte, HSC, and cholangiocyte identities are overlapping across annotation strategies, and two out of three strategies also indicated a small embryonic‐stem cell population as a fourth cell type in day 21 HLOs. Based on this comparative analysis we decided to utilize the ScType (Ianevski *et al*, 2022) database annotation method for all subsequent analyses since it allows for the annotation of potentially emerging cell types beyond the repertoire of a literature list or a single reference study. To account for the *in vitro* generation of the cells, we refer to them as cell type*‐like* in this study.

We confirmed that each cluster was represented in all replicates (Appendix Fig S2e and f) and further ensured cellular identities by evaluating the expression of canonical marker genes for each cell type compared to consensus annotations (Fig 1E). These results support the annotations from the previous strategies, however, *KRT19* was still positive in the hepatocyte‐like population, indicating they retained premature features on day 21 in HLOs. We then performed pathway enrichment analysis, revealing upregulated signatures for liver‐characterizing metabolic processes and lipid metabolism in hepatocyte‐like cells. HSC‐like cells were enriched for pathways related to extracellular matrix and focal adhesion. Cholangiocyte‐like cells showed enrichment for pentose phosphate metabolism and sphingolipids (Fig 1F). Together, these results indicate the presence of hepatocyte‐, HSC‐, and cholangiocyte‐like cells in day 21 HLOs across cell type annotation strategies.

### Hepatocyte maturation and cell type distribution are regulated by mechanical culture conditions

**Figure 2:**
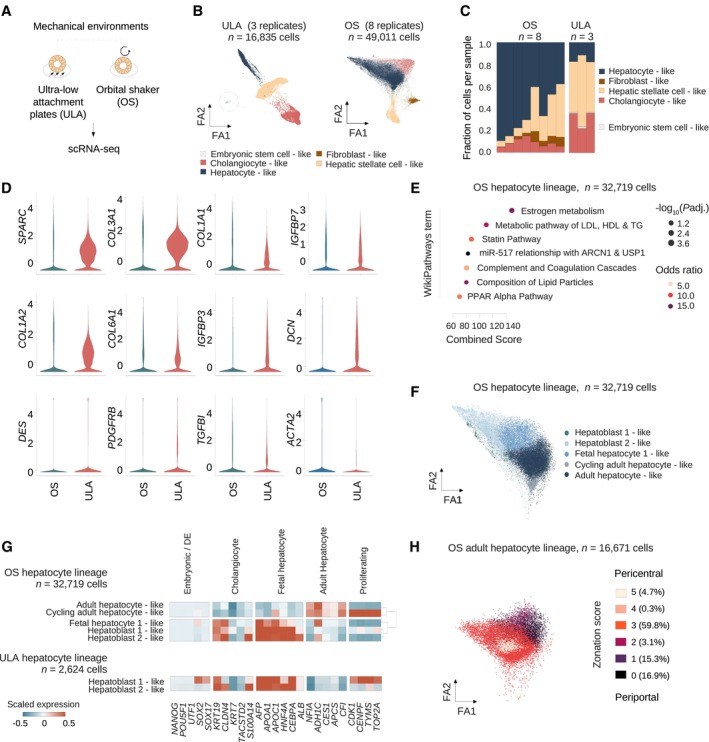
Hepatocyte maturation and cell type distribution are regulated by mechanical culture conditions AOverview of the two mechanical environments selected for 10× scRNA‐seq.BForceAtlas2 plots mapping cells from control ULA‐HLOs (cultured in ultra low attachment plates, *n =* 16,835 cells, *n* = 3 replicates) and control OS‐HLOs (cultured in orbital shaker, *n =* 49,011 cells, *n* = 8 replicates). Dotted line highlights the embryonic stem cell‐like population, which is only present in ULA‐HLOs. ScType database annotations in OS‐HLOs: Hepatocytes (*P* = 2.56E‐07), Cholangiocytes (*P* = 0.01), Hepatic stellate cells (*P* = 1.3E‐10), Fibroblasts (*P* = 0.003).COS‐HLOs show a relative reduction of HSC‐ and cholangiocyte‐like cells while the fraction of hepatocyte‐like cells is increased. Barplots showing the cell cluster proportions as the fraction of total cells per sample for each of the individual OS‐ and ULA‐HLO replicates. The color indicates the cell type as annotated in (B), and cell types are listed in the order they are displayed.DViolin plots displaying the normalized, scaled expression of hepatic stellate cell marker genes in OS‐ and ULA‐HLOs across all cells. Corresponding ForceAtlas2 representations are provided in Appendix Fig S2h.EDoplot showing the top enriched WikiPathways (Martens *et al*, 2021) terms by combined score. Dot sizes correspond to negative decadic logarithm of the adjusted *P*‐value, colors represent the odds ratio. Terms have been shortened for readability.FForceAtlas2 plots mapping hepatocyte‐like cells from control OS‐HLOs. Cells were annotated to the human fetal liver development atlas (Wesley *et al*, 2022). *n* = 32,719 cells (*n* = 8 controls).GHLOs exposed to the OS‐environment show KRT19^low^ hepatocyte subpopulations expressing mature hepatocyte markers. Matrixplot shows the scaled mean expression for marker genes for stages of hepatocyte development for OS‐hepatocyte lineage subclusters (top) as annotated in (F). in comparison to ULA‐hepatocyte‐like subpopulations (bottom). Marker genes (bottom) are sorted by cell type (top). Dendrogram representing hierarchical clustering for lineages with > 2 hepatocyte‐like subgroups is shown on the right. ULA: *n* = 2,624 cells (*n* = 3 controls), OS: *n* = 32,719 cells (*n* = 8 controls). Annotations were selected based on the highest‐ranked annotation by marker gene overlap. *P*‐values (sub‐clustered ULA populations): HB1 (cluster 1, *P* = 0.99), HB1 (cluster 2, *P* = 0.99), HB1 (cluster 3, *P* = 0.99), HB2 (cluster 4, *P* = 0.99), HB2 (cluster 1, *P* = 0.92), HB2 (cluster 2, *P* = 0.98). Sub‐clustered OS‐populations: AH (*P* = 0.94), HB2 (*P* = 0.46), HB1 (*P* = 0.79), FH1 (*P* = 0.88), FH2 (*P* = 0.733), cluster 6 (*P* = 0.73, manually annotated as cycling AH, Appendix Fig S2g).HAdult hepatocyte‐like cells from HLOs exposed to the OS‐environment display hepatocyte zonation. ForceAtlas2 plots mapping OS‐adult hepatocyte‐like cells from (F). Color indicates the hepatocyte zonation score as calculated based on marker genes from a human adult liver scRNAseq reference (MacParland *et al*, 2018) (Materials and Methods). *n* = 16,671 cells (*n* = 8 controls).

To gain insights into the differences between HLOs cultured under different conditions, we compared HLOs conventionally cultured on an ultra low attachment plate with 10% Matrigel (Thompson & Takebe, 2020) (ULA‐HLOs) and HLOs isolated from Matrigel and cultured on an orbital shaker (OS‐HLOs, Fig 2A). We analyzed a total of 11 HLO scRNA‐seq samples on ULA plates (*n* = 3 ULA day 21, Fig 1D) and control HLOs on the OS (*n* = 8 from day 25) (Fig 2B). After joint QC and normalization, our dataset contained a total of 16,835 single cells from ULA‐HLOs and 49,011 single cells from OS‐HLOs. We next ran our standard pipeline and determined optimal clustering resolution by calculating cluster robustness metrics (Appendix Fig S2g), which detected four cell clusters in each HLO culture context, and projected the clusters on a force‐directed layout for optimal global structure preservation (Huang *et al*, 2022). ULA‐HLO cells displayed the same cell types shown in Fig 1D, and the OS‐HLOs were annotated as hepatocyte‐, cholangiocyte‐, HSC‐, and fibroblast‐like cells (Fig 2B). No cluster of embryonic stem cell‐like cells was identified in OS‐HLOs. Stem cells were additionally searched by using an algorithm specifically designed to detect rare cell types (Lubatti *et al*, 2023) and were not detected. We further observed that ULA‐HLOs contained a greater proportion of cholangiocyte‐like cells, while the hepatocyte‐like cell population expanded in OS‐HLOs (Fig 2C). We also found higher expression levels of HSC marker genes in ULA‐HLOs (Fig 2D, Appendix Fig S2h). Together these results indicate that OS‐HLOs show a distribution of cell types that more closely resembles the adult liver compared to ULA‐HLOs. Importantly, OS conditions display a reduced expression of collagen transcripts and lowered average HSC fractions, potentially reflecting a reduced baseline fibrotic activity.

We next focused on the hepatocyte lineage. OS‐hepatocyte‐like cells showed enrichment in genes involved in specialized hepatocyte metabolic processes, such as estrogen metabolism and coagulation‐related pathways in addition to metabolic and lipid particle processes (Fig 2E). We then annotated OS‐hepatocyte‐like cells with marker genes for hepatocyte development (Wesley *et al*, 2022) in order to derive the closest matching hepatocyte identity based on marker gene expression (Fig 2F, Materials and Methods). which revealed a mix of hepatoblast‐, fetal hepatocyte‐, and adult hepatocyte‐like cells (Dataset EV3). A cluster mapping close to the adult hepatocyte‐like cells containing cells in G2M phase was termed cycling adult hepatocyte‐like cells (Appendix Fig S2i). We next evaluated the expression of marker genes for normal hepatocyte development stages (Wesley *et al*, 2022) across the jointly preprocessed and normalized cells from OS‐ and ULA‐HLOs (Fig 2G). Unlike OS‐HLOs which contained clusters across hepatocyte development, ULA‐HLO clusters were all categorized as hepatoblast‐like (Dataset EV3). Consistent with a less mature phenotype, *SOX2* and *SOX17* were still expressed in one ULA‐HLO hepatocyte cluster. Furthermore a broad proportion of ULA‐HLO hepatocytes expressed proliferation markers, in contrast to only two hepatocyte subclusters in OS‐HLOs. Most importantly, OS‐HLOs displayed a major sub‐cluster of *KRT19* ^low^ cells expressing adult hepatocyte‐markers *NFIA*, *ADH1C*, *CES1*, *APCS* and *CFI*. To understand the zonal composition of the non‐cycling adult hepatocyte‐like cells, we sub‐clustered the 16,671 adult hepatocyte‐like cells and calculated a hepatocyte zonation score based on marker genes from a human adult liver scRNA‐seq reference (MacParland *et al*, 2018) (Materials and Methods). This revealed adult hepatocyte‐like cells representing periportal, interzonal, and pericentral zones (Fig 2H), with a bias towards interzonal hepatocytes. Together, these analyses suggest a more mature transcriptional landscape of hepatocyte‐like cells developed with OS culture conditions.

### Inducible liver injury phenotypes in HLOs upon TGF‐β1 and fatty acid treatment

**Figure 3:**
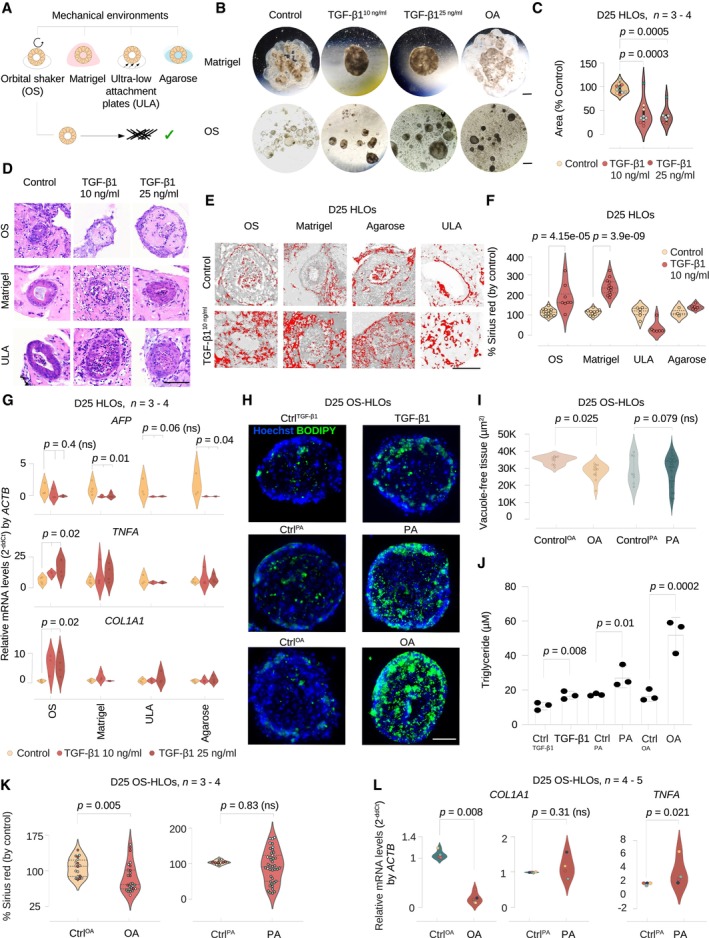
Inducible liver injury phenotypes in HLOs upon FFA and TGF‐β1 treatment AOverview of the four mechanical environments evaluated for their potential to promote collagen induction with injury models.BHLOs in the Matrigel‐ and OS‐environments display morphologic changes when treated with TGF‐β1 and OA. Brightfield images of whole Matrigel domes with embedded HLOs (upper panel, scale bar 3 mm) and HLOs isolated from Matrigel in the orbital shaker environment (lower panel, scale bar 0.1 mm) after 4 days of treatment with TGF‐β1 (10, 25 ng/ml), OA (400 μM), and controls. Contraction of the Matrigel dome with TGF‐β1 treatment and darkening of color (among all treatments) was observed.CCompaction assay for Matrigel‐environment cultured HLOs indicates a significant surface area reduction for HLOs treated with TGF‐β1. Violin plots displaying the Matrigel drop area normalized by the control. Kruskal–Wallis test (two‐tailed) followed by a *post hoc* Conover's test with Bonferroni correction. Dot colors represent experiments, circles represent individual technical replicates. *N* = 3–4 experiments.DH&E stainings of OS‐HLOs (upper row), Matrigel‐HLOs (middle row), and ULA‐HLOs (lower row) after 4 days of treatment with TGF‐β1 (10, 25 ng/ml) display wall thickening in Matrigel‐ and ULA‐ HLOs while hyaline‐like intra‐organoidal mass accumulation is present only in OS‐HLOs. Scale bar 100 μm. Precise numbers of technical replicates are represented by circles. *N* = 1 experiment.EThresholded images of Sirius red‐stained HLOs cultured in four different environments stimulated with TGF‐β1 (10 ng/ml) show collagen deposition in Matrigel‐ and OS‐HLOs. Scale bars 100 μm. 3 HLOs (technical replicates). *N* = 1–2 experiments.FSirius red staining quantification indicates significant collagen fiber deposition only in HLOs exposed to Matrigel‐ and OS‐environments during stimulation with TGF‐β1 (10 ng/ml). Violin plots showing the percentage of Sirius red positive tissue normalized by the mean of the control. Horizontal lines in violin plots represent quartiles. Kruskal–Wallis (two‐tailed) test followed by a *post hoc* Conover's test with Bonferroni correction. Precise numbers of technical replicates per group as represented by circles. *N* = 1 experiment.GqRT‐PCR for *COL1A1*, *TNFA*, and *AFP* indicates a significant induction of *COL1A1* and *TNFA* transcripts only in HLOs stimulated in the OS‐environment. Violin plots showing relative mRNA levels normalized to *ACTB* for each mechanical environment ($2^{-\Delta \Delta C_{t}}$). Kruskal–Wallis (two‐tailed) test followed by a *post hoc* Conover's test with Bonferroni correction comparing three treatment groups per culture method. *P*‐values as indicated in the figure, ns, not significant. *N* = 3–4 experiments.HBODIPY staining of lipids in HLOs cultured in the OS‐environment with exposure to TGF‐β1 and FFAs. Representative BODIPY immunofluorescence images of day 25 OS‐HLOs treated TGF‐β1, PA, and OA (right) or the control solutions (left). BODIPY staining (green), nuclear DNA staining with Hoechst (blue), scale bar, 50 μm. *N* = 2 experiments. See Appendix Fig S3c for analysis of HLOs derived from a second PSC line.ITissue quantification for 200 × 200 μm regions of interest (ROIs) around single HLOs treated with OA, PA, and their respective controls. Kruskal–Wallis test (two‐tailed) followed by a *post hoc* Conover's test with Bonferroni correction. *P*‐values as indicated in the figure, ns, not significant. 66 HLOs (technical replicates). *N* = 3 experiments.JTriglyceride (TAG) content is quantified in OS‐HLOs treated with TGF‐β1, PA, and OA for 4 days. Bar plots display the total TAG concentration in μM for a representative experiment. Individual dots represent technical replicates, bar plots show mean ± SD. Kruskal–Wallis test (two‐tailed) followed by a *post hoc* Conover's test. *P*‐values as indicated in the fig *N* = 1 experiment.KSirius red quantification in HLOs treated with OA, PA, and their controls. Shown is the percentage of Sirius red positive tissue by total tissue (normalized by the mean of the control HLOs in each experiment). Quantification for 200 × 200 μm regions of interest (ROIs) around single HLOs. Horizontal lines in violin plots represent quartiles. Kruskal–Wallis test (two‐tailed) followed by a *post hoc* Conover's test with Bonferroni correction, ns, not significant. 95 HLOs (technical replicates). *N* = 3–4 experiments.L
*COL1A1* expression is reduced in OS‐HLOs treated with OA for 4 days, *TNFA* expression is induced in OS‐HLOs treated with PA for the same duration. Shown are qPCR results for relative mRNA levels of *COL1A1* and *TNFA* normalized to *ACTB*. Mann–Whitney‐*U* test (two‐tailed). *P*‐values as indicated in the figure, ns, not significant. *N* = 3–5 experiments (represented by individual dot colors). Source data are available online for this figure.

We next evaluated conditions to induce a fibrotic phenotype in the HLO model. TGF‐β1 drives liver fibrogenesis *in vivo* (Kanzler *et al*, 1999; Kim *et al*, 2018), and we treated HLOs with TGF‐β1 (10 and 25 ng/ml) in four different 3D culture systems and evaluated collagen expression, as a metric of fibrosis. The final differentiation steps for HLOs are performed in Matrigel, and HLOs were either left in Matrigel or removed from Matrigel and cultured on (i) ULA plates (Ouchi *et al*, 2019), (ii) 1% agarose coated plates, and (iii) an orbital shaker (Fig 3A).

We observed morphological changes in HLOs cultured in Matrigel and on the orbital shaker after 4 days of TGF‐β1 treatment, including HLO tissue consolidation, and surface roughening (Fig 3B, Appendix Fig S3a). For comparison, we also evaluated HLOs treated with OA, which did not demonstrate the same compaction in Matrigel as observed with TGF‐β1 but was associated with a darker appearance on light microscopy when cultured in the orbital shaker, as previously described (Ouchi *et al*, 2019). These results suggest HLOs are reacting in an injury‐specific manner to the applied treatments.

We further quantified the contractile effect of TGF‐β1 by culturing HLOs in Matrigel drops and measuring the Matrigel drop area after TGF‐β1 application. This analysis demonstrates a significant reduction in droplet size with TGF‐β1 treatment, consistent with increased contractile activity (Fig 3C). To further investigate alterations in HLOs we stained for H&E and observed the intraluminal accumulation of hyaline, monomorphic structures upon TGF‐β1 treatment (Fig 3D), providing further evidence of an injury response to TGF‐β1 treatment.

We next performed Sirius red staining (Fig 3E), a standard method for quantification of type I and III collagen deposition in liver fibrosis (Huang *et al*, 2013). We optimized an existing pipeline (Materials and Methods) for the computational quantification of Sirius red staining in liver for sections with low tissue amounts and options to select individual HLO areas of interest for the calculation of Sirius red percentage per tissue and per area separately (Materials and Methods). We utilized our pipeline to analyze HLOs cultured via the four previously described methods and treated with TGF‐β1 (10 ng/ml) for 4 days. We found a significant increase in collagen deposition only in Matrigel and OS‐HLOs (Fig 3F), suggesting the latter culture methods lead to accumulation of type I and III collagen as observed in liver fibrosis.

We next evaluated gene expression response across the four culture methods in HLOs when treated with TGF‐β1. Canonical transcriptional changes in steatohepatitis include the activation of *TNFA* (Loomba *et al*, 2021), while induction of the alpha‐1 subunit of type 1 collagen (*COL1A1*) is characteristic of fibrosis (Ramachandran *et al*, 2019). We analyzed mRNA levels for the respective genes. We found that only OS‐HLOs with TGF‐β1 showed a significant increase in *TNFA* and *COL1A1* expression (Fig 3G), while the fetal liver transcript alpha‐fetoprotein (*AFP*) was reduced in HLOs cultured on Matrigel and 1% agarose, potentially reflecting different levels of TGF‐β1‐responsive progenitor‐like cells across culture conditions (Damdinsuren *et al*, 2006; Yang *et al*, 2016). These results show that culturing in the orbital shaker provides the conditions under which HLOs demonstrate the most robust inflammatory and fibrotic response to TGF‐β1.

We therefore focused on the orbital shaker method to evaluate the response to fatty acids. Treatment with OA and PA resulted in the formation of lipid droplets, observed as stain‐free vacuoles on H&E (Appendix Fig S3b, arrowheads). Treatment with TGF‐β1, OA, and PA resulted in the formation of lipid droplets, observed as BODIPY enrichment in immunofluorescence imaging, with OA inducing the greatest accumulation of lipid droplets (Fig 3H). These results were confirmed in HLOs derived from an additional PSC‐line (Appendix Fig S3c). We also assessed lipid accumulation by quantifying the proportion of stain‐free tissue in regions of interest (ROIs, 200 × 200 μm) capturing individual organoids. Only OA‐treated HLOs showed significant tissue rarefaction due to steatosis (Fig 3I, Appendix Fig S3d). In addition, triglyceride levels increase in each condition, with OA showing the greatest increase and TGF‐β1 the least increase in two PSC lines (Fig 3J, Appendix Fig S3e).

We then sought to understand if OA and PA could induce collagen production. Surprisingly, OA treatment led to a reduction in Sirius red staining and the quantified percentage of Sirius red positive tissue, as a marker of collagen deposition, while PA did not change Sirius red staining (Fig 3K, Appendix Fig S3f). In line with these results, treatment with OA significantly reduced *COL1A1* mRNA levels at the transcriptional level, and *COL1A1* levels did not change with PA (Fig 3L). However, PA treatment led to a significant increase in *TNFA* mRNA levels, whereas OA was associated with a significant reduction in *TNFA* at 400 μM concentration (Fig 3L, Appendix Fig S3g). These results show that OA induced more robust steatosis, while PA induced *TNFA*, consistent with an inflammatory response. Neither OA nor PA induced fibrotic injury, with OA treatment actually reducing levels of collagen at both the transcriptional and protein level.

### Oleic acid, palmitic acid, and TGF‐β1 induce distinct inflammatory and fibrotic responses at the single‐cell level

**Figure 4:**
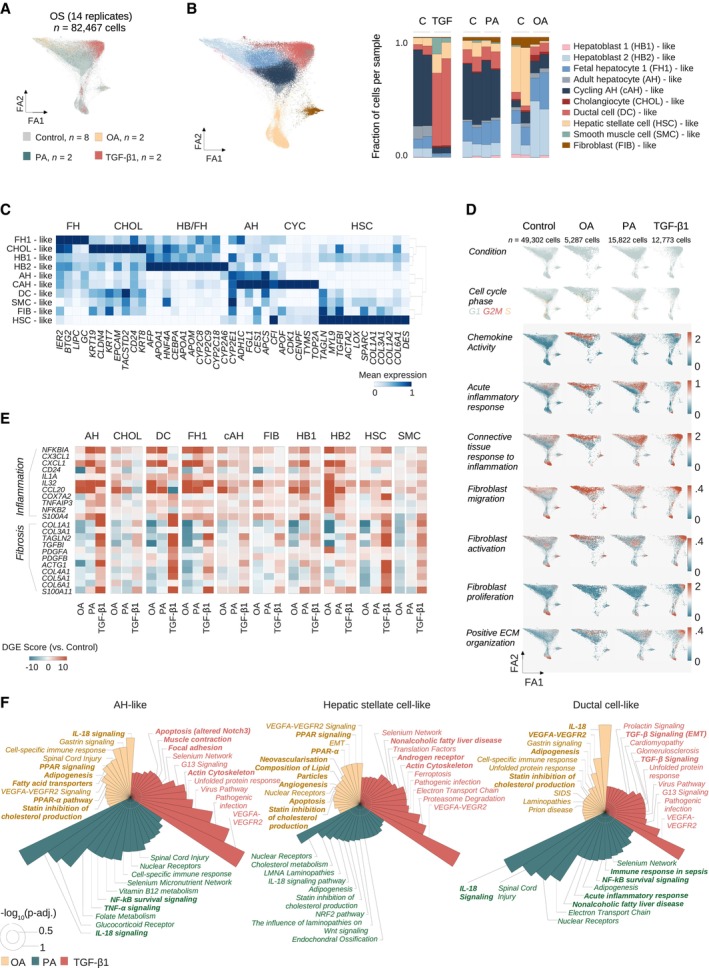
Oleic acid, palmitic acid, and TGF‐β1 induce distinct inflammatory and fibrotic responses at the single‐cell level ForceAtlas2 representation of cells from OS‐HLOs treated for 4 days with OA (500 μM), PA (500 μM), or TGF‐β1 (10 ng/ml), and their respective controls, colored by treatment condition. *N* = 82,467 cells from 14 replicates (*n* = 2 replicates per condition, *n* = 8 controls).ForceAtlas2 representation from (A), colored by cell type as annotated with the ScType (Ianevski *et al*, 2022) database and hepatocyte‐like cells resolved to the human fetal liver development atlas (Wesley *et al*, 2022) annotation (Materials and Methods). Barplot to the right shows cell cluster proportions as the fraction of total cells per sample for each of the individual replicates. Color encodes cell type annotation, and annotations are listed in the order they are displayed. *P*‐values for sub‐clustered populations: Ductal cell‐like 1 (*P* = 2E‐05), ductal cell‐like 2 (*P* = 0.88, manually annotated, Materials and Methods, Appendix Fig S4d), smooth muscle cell‐like (*P* = 6.99E‐07), cholangiocyte‐like (*P* = 3.2E‐06).Matrixplot shows the scaled mean expression for marker genes in each cluster from (B). Canonical marker genes (bottom) are sorted by cell type (top). Hierarchical clustering is represented by the dendrogram on the right. *N* = 82,467 cells from 14 replicates (*n* = 2 replicates per condition, *n* = 8 controls). AH, adult hepatocyte; CHOL, cholangiocyte; CYC, cycling; FH, fetal hepatocyte; HB, hepatoblast; HSC, hepatic stellate cell.OA and PA induce inflammatory signals, and TGF‐β1 induces expanded fibrotic signatures. ForceAtlas2 plots show GO term (The Gene Ontology Consortium, 2000; Carbon *et al*, 2021) scores for selected categories related to inflammation and fibrosis (labeled, left) for HLOs treated with OA, PA, TGF‐β1, and controls (labeled, top). Increased expression is indicated by a shift from blue to red. Cell cluster annotations are provided in (B). Scale bars indicate the expression score. *N =* 14 replicates (*n* = 2 replicates per condition, *n* = 8 controls). Cell numbers are indicated.FFAs induce an inflammatory signature, TGF‐β1 induces a fibrotic signature, and OA ameliorates the fibrotic signature in HLOs. Cell clusters were separated for expression analysis (top). Differential expression score (pairwise comparison between treatment‐specific controls and treatments, Materials and Methods) for selected genes associated with fibrosis and inflammation for cell types indicated on the top. Differential gene expression (DGE) data are provided in Dataset EV6. AH, adult hepatocyte‐like; CHOL, cholangiocyte‐like; DC, ductal cell‐like; FH1, fetal hepatocyte 1‐like; FIB, fibroblast‐like; HB1, hepatoblast 1‐like; HB2, hepatoblast 2‐like; HSC, hepatic stellate cell‐like; SMC, smooth muscle cell‐like.Top enriched WikiPathways (Martens *et al*, 2021) terms for treatment‐specific differentially expressed genes in three HLO cell populations corresponding to (B), as labeled. Circular bar plots display the negative decadic logarithm of adjusted *P*‐values for the respective terms. Cut‐off for plots is an adjusted *P*‐value below 0.05. White circles indicate negative decadic logarithm of adjusted *P*‐values of 0.5 (inner white circle) and 1 (outer white circle). Terms have been shortened for readability, full lists and further cell clusters are provided in Appendix Fig S5.

To dissect cell‐type‐specific transcriptional injury‐response patterns, we treated HLOs cultured on an orbital shaker with OA, PA, or TGF‐β1 for 4 days before performing scRNA‐seq (Fig 4A). We analyzed 82,467 cells after QC (Materials and Methods) from 14 HLO samples, annotating cell types with the scType database. We next incorporated hepatocyte sub‐stages (Fig 2G) and sub‐clustered cholangiocyte‐like cells in order to resolve shifts within major HLO populations under treatment conditions. This yielded two new sub‐clusters with expression patterns associated with ductal cell (DC)‐like, and smooth muscle cell (SMC)‐like cells (Fig 4B, left, Appendix Fig S4a–e). The DC‐like 2 population did not meet statistical significance for a specific cell type (Dataset EV9), and was assigned to the DC‐like cluster after evaluating marker gene expression and distance dendrograms (Appendix Fig S4d).

We then evaluated how cell type distributions change with each condition. TGF‐β1‐treated HLOs displayed a significant increase in HSC‐related populations. HSC‐like cells increased from < 10% in controls (5.4 and 6.6%) to > 10% (15.7 and 13.5%) with TGF‐β1 treatment. DC‐like cells increased significantly from 4 and 7% in the controls to 61 and 72%, potentially mirroring the ductular reaction seen in chronic liver injury. SMC‐like cells increased significantly from 0.2% to 14 and 37% with TGF‐β1 treatment. In contrast, hepatocyte‐like populations decreased by more than 10‐fold from 59 to 63% to ~5%, and a near‐total loss of cycling cells was observed, though not reaching statistical significance (Fig 4B, right, Dataset EV3), in line with previously reported cell cycle arrest through TGF‐β1 (Laiho *et al*, 1990; Sheahan *et al*, 2007). While the analysis shows an expansion of HSC‐, and ductal‐like cells in response to TGF‐β1, as observed in the development of liver fibrosis (Raven *et al*, 2017; Deng *et al*, 2018; Kisseleva & Brenner, 2021), no significant alterations in cell population proportions were observed with PA treatment.

In contrast, OA‐treated HLOs showed a dramatic and statistically significant reduction of HSC‐like cells from 38 and 48% to 1.9 and 1.6%, even though HSCs were comparatively high in this batch of OS‐HLOs (see for Fig 2C). SMC‐ and fibroblast‐like cells were not affected. Conversely, adult‐, fetal‐, and most strikingly hepatoblast 2‐like cells expanded under OA treatment, the latter from 26 and 8% in controls to 47 and 39% in OA‐treated HLOs, and these effects were statistically significant for fetal hepatocyte 1‐ and hepatoblast 2‐like cells. Zonal analysis of adult hepatocyte‐like cells revealed a relative loss of pericentral hepatocyte‐like cells with TGF‐ꞵ1 (Appendix Fig S4f). These results suggest that OA treatment in HLOs reduces HSC‐like cells and expands hepatocyte‐like cells, specifically hepatoblast‐like sub‐populations.

Variability in the relative number of cell types can be observed with each batch of HLO differentiation, as observed for HSCs in control conditions (Fig 4B, right). To confirm a reduction in HSCs in response to OA, we repeated our analysis using *DES* expression as a marker of relative HSC abundance in HLOs (Fig 4C). HLOs differentiated from the original PSC line and those differentiated from a second iPSC line both showed significant reduction in *DES* expression with OA treatment, which did not occur with either TGF‐ꞵ1 or PA (Appendix Fig S4g and h). In both cases, the loss of HSCs was also accompanied by reduced expression of *COL1A1* with OA treatment, as previously observed (Fig 3I). These results provide additional evidence to support the conclusion that OA reduces the number of HSC‐like cells. Evaluation of marker gene profiles revealed that DC‐like cells were positive for *KRT19* and *HNF4A* as previously reported for a bi‐potent cell type induced under conditions of chronic liver injury (Deng *et al*, 2018; Pu *et al*, 2023). Interestingly, DC‐like, SMC‐like, and fibroblast‐like cells acquired a myofibroblast‐like gene expression signature (*TAGLN*, *MYL9*, *SPARC*) accompanied by a reduction in the expression of ductal markers *EPCAM*, *CD24*, and *CLDN4* (Fig 4C).

To define gene expression signatures activated with each treatment, we evaluated injury‐response scores based on gene ontology (The Gene Ontology Consortium, 2000) term scoring. OA and PA‐treated HLOs showed induction of immune response‐related scores when compared to their controls and were enriched for chemokine‐activity associated genes, as well as fibroblast migration and activation signatures (Fig 4D). TGF‐β1 treatment increased scores for fibroblast proliferation and positive regulation of ECM organization along with alterations in cell cycle phase distributions, while these scores remained mostly unchanged in the OA and PA‐treated HLOs. Cells with induced inflammatory scores under OA treatment were primarily hepatoblast‐like cells, while PA‐treated cells were more broadly enriched for inflammatory scores. Induction of pro‐fibrotic scores with TGF‐β1 occurred mostly in the mesenchymal clusters (HSC‐, fibroblast‐like cells) and DC‐like cells.

Differential gene expression analysis per cell cluster further displayed injury‐specific signatures depending on the respective cell type. The fibrotic module included *COL1A1*, *TAGLN2*, and *TGFBI* and was induced across TGF‐β1 treated cell types, with AH‐, DC‐, and HSC‐like cells showing the strongest relative induction (Fig 4E). The inflammatory module of differential genes included Interleukin 32 *(IL32)* (the top up‐regulated gene in MASLD, Baselli *et al*, 2020) and other canonical inflammatory genes including *NFKBIA* and *CCL20* (Fig 4E, Dataset EV6), showing the strongest relative induction in AH‐, and hepatocyte precursor‐like cells treated with PA, followed by DC‐ and HSC‐like populations. Interestingly, OA caused a relative reduction of the fibrotic signature across almost all cell types (indicated by a shift towards blue color). Furthermore, the inflammatory module showed only moderate changes in OA‐treated adult hepatocyte‐like cells, while the expanding precursors, especially the hepatoblast 2‐like cells increased the expression of inflammatory genes.

To contextualize differential gene expression more broadly, we performed cell‐type‐resolved pathway analysis and found TGF‐β1‐treatment induced apoptosis‐, and cytoskeleton‐related pathways in AH‐like cells, while HSC‐like cells were enriched for genes associated with MASLD (Fig 4F, Appendix Fig S5). Strikingly, PA‐treated DC‐, hepatoblast 1 and 2‐, and fibroblast‐like cells displayed the MASLD pathway among their top‐ranked enriched terms. Additionally, genes associated with TNFɑ‐signaling were induced in PA‐treated AH‐like cells. OA treatment resulted in lipid‐metabolism related hits. Together, these analyses show that PA and TGF‐β1 mirror MASLD‐associated inflammatory responses, while the OA‐related signature is dominated by genes involved in adipogenesis, fat metabolism, and steatosis alongside a general inflammatory response mainly driven by hepatoblast‐like cells.

### OA induces an interactome distinct from crosstalk observed with TGF‐β1 and PA treatment

**Figure 5:**
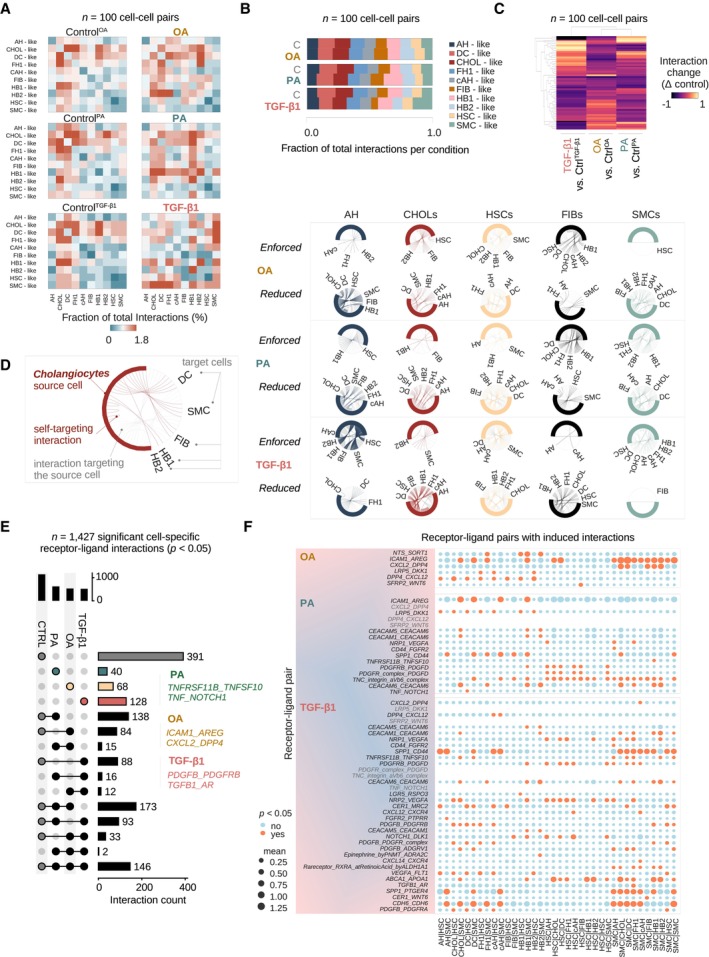
OA induces an interactome distinct from crosstalk observed with TGF‐β1 and PA treatment CellPhoneDB (Efremova *et al*, 2020) analysis of cell–cell interactions in healthy and injured HLOs. Heatmaps show the fraction of total interactions per condition in day 25 OS‐cultured HLOs treated with OA, PA, TGF‐β1, and their respective controls. HSC, hepatic stellate cell‐like; AH, adult hepatocyte‐like; cAH, cycling adult hepatocyte‐like; CHOLs, cholangiocyte‐like; DC, ductal cell‐like; FH1, fetal hepatocyte 1‐like; FIB, fibroblast‐like; HB1, hepatoblast 1‐like; HB2, hepatoblast 2‐like; SMC, smooth muscle cell‐like. *N* = 100 possible cell–cell interactions per condition, *n* = 2 replicates per condition, *n* = 6 controls.Stacked bar plots show the fraction of total interactions per condition for all cell clusters, where the respective cell is the source of the interaction. Abbreviations are as listed in (A). Cell clusters are colored in the order of their appearance. *N* = 100 possible cell–cell interactions, *n* = 2 replicates per condition, *n* = 6 controls.Clustered heatmap shows the difference in the fraction of total interactions between each treatment and its control group. Dendrograms show the hierarchical clustering by condition (top) and cell–cell interaction (left). *N* = 100 possible cell–cell interactions, *n* = 2 replicates per condition, *n* = 6 controls.Chord diagrams show the fraction of total interactions per source cell with its respective target cells in HLOs treated with OA, PA, and TGF‐β1 relative to their controls. Chord diagrams are divided by interactions that have (i) a higher fraction of total interactions in treatment conditions (“enforced”), and (ii) interactions with a lower fraction of total interactions (“reduced”) compared to controls. Colored lines indicate interactions initiated by the source cell in each column, while gray lines show interactions of other cell types targeting the source cell. *N* = 100 possible cell–cell interactions, *n* = 2 replicates per condition, *n* = 6 controls.UpSet plot displays the numbers of intersecting and unique significant (*P*‐value < 0.05) cell‐type‐specific receptor‐ligand interactions of HLOs treated with OA, PA, TGF‐β1, and controls. Examples for receptor‐ligand pair categories containing unique interactions for each treatment are displayed (Materials and Methods). *N* = 1,427 significant cell–cell partner‐specific receptor‐ligand pair interactions, *n* = 2 replicates per condition, *n* = 6 controls.Dotplots show receptor‐ligand pairs with an induced number of significant cell–cell interactions in HLOs treated with OA, PA, TGF‐β1, compared to their respective controls. Receptor‐ligand pairs with at least two induced interactions are shown. Mean expression (mean of all partners' individual average expression, Materials and Methods) is indicated by the circle size, and significant *P*‐values < 0.05 are indicated by orange color. An interaction can be non‐significant despite high mean expression among two partnering molecules. Receptor‐ligand pairs are shown on the *x*‐axes, and cell clusters are shown on the *y*‐axes. *N* = 2 replicates per condition, *n* = 6 controls. Source data are available online for this figure.

To understand the cell–cell interactions in OA, PA, and TGF‐β1 injury models, we utilized CellPhoneDB (Efremova *et al*, 2020) and assessed the relative abundance of interactions between each cell type. Overall, the relative induction of interactions increased from OA‐, to PA‐, to TGF‐β1‐treated HLOs (Fig 5A). OA moderately induced interactions between hepatoblast 2‐, cholangiocyte‐, and DC‐like cells (HB2, CHOLs, DCs). PA‐mediated crosstalk was mainly driven by cholangiocyte‐like, hepatocyte progenitor‐, fibroblast‐ and SMC‐like cells (CHOLs, DCs, HB1, FIBs, SMCs). Only TGF‐β1 yielded a strong activation in cell–cell communication driven by SMC‐ (most likely representing myofibroblast‐like cells) and HSCs‐like cells. TGF‐β1 also induced HSCs‐SMC‐like cell–cell interactions and HSC‐AH‐like interactions (Fig 5A). Plotting the proportion of interactions for each cell type revealed the relative reduction of SMCs‐involving interactions in OA‐treated HLOs, and their relative induction with TGF‐β1 (Fig 5B). Hierarchical clustering of the delta values between control and treatment fractions of each interaction suggested that TGF‐β1 induced an interaction pattern more distinct from the ones characterizing HLOs treated with PA and OA (Fig 5C, Appendix Fig S6a). These evaluations of the interactome reveal a hierarchical increase in the relative interaction changes induced, in particular with respect to fibrosis‐related cell types (HSCs, SMCs), from OA‐, to PA, to TGF‐β1 treatment.

We next aimed to understand which interactions were enforced or reduced with each treatment when compared to its control. We plotted chord diagrams for each cell type and each case, where “enforced” indicates a positive delta value compared to the control and “reduced” indicates a negative delta value (Fig 5D, Appendix Fig S6b). This analysis showed that in OA‐treated HLOs, particularly SMC‐ and HSC‐like cells lost more interactions than they gained, and this was in line with the general decrease in interaction abundance in these cell types (compare Fig 4B). In PA‐treated HLOs the fraction of SMC‐like mediated interactions were shifted in favor of targeting hepatocyte precursors and HSC‐like cells. Even though their proportion in cell numbers was not changed with PA (compare Fig 4B), adult hepatocyte‐like sourced interactions were shut down across many interacting partners with PA in favor of enforced communication with hepatoblast 1‐ and HSC‐like cells. In TGF‐β1‐treatment, we observed an increased fraction of SMC‐like cell interactions (Fig 5B). These cells globally expanded their interactions and shifted their crosstalk specifically towards each other in the presence of TGF‐β1. Together, these results suggest OA‐mediated reduction in HSC‐ and SMC‐like cellular crosstalk while TGF‐β1 promotes crosstalk between these cell types.

As a next step, we identified cell‐type‐resolved receptor‐ligand pairs exclusive for each injury type. With PA treatment, receptor‐ligand expression involving *TNF superfamily (TNFS)* members was observed, reflecting the induction of previously described molecular mediators of cellular responses to injury and inflammation in the liver (Anstee *et al*, 2019). In TGF‐β1‐treated HLOs, receptor‐ligand expression was observed involving *PDGFB* and *PDGFRB*, recapitulating an important hallmark of liver fibrosis *in vivo* (Kocabayoglu *et al*, 2015; Ying *et al*, 2017; Ramachandran *et al*, 2019). We further identified *CXL2* and *DPP4* ligand‐receptor pair expression signatures indicative of OA treatment. Inhibitors of the exopeptidase dipeptidyl‐peptidase 4 (DPP4) are anti‐diabetes drugs and have been shown to reduce steatosis in MASLD patients (Androutsakos *et al*, 2022), though failing to consistently ameliorate liver inflammation and fibrosis in larger meta‐analyses (Dos Santos *et al*, 2021). The induction of predominantly steatosis‐related interacting transcripts is in line with our previous analyses where OA induced steatosis in HLOs without fibrosis (Fig 3) and had more localized effects on the broad inflammatory response. This evaluation of the interactome reveals groups of receptor‐ligand pairs changing in response to inflammatory and fibrotic injury in HLOs overlapping with molecular mediators previously reported *in vivo*.

We then examined injury‐specific interactions between individual receptor‐ligand pairs (Fig 5F). These analyses revealed new *DPP4‐CXCL12* pairs with OA treatment potentially allowing hepatocyte‐like lineages and fibroblast‐like cells to cleave CXCL12 expressed by HSC‐like cells. The expression of TNF‐Related Apoptosis Inducing Ligand (TRAIL), encoded by TNF superfamily member *TNFS10*, in linkage with its decoy receptor Osteoprotegerin (OPG) (Emery *et al*, 1998), encoded by *TNFRSF11B*, was observed exclusively between HB‐like cells (*TNFS10*) and SMC‐like cells (*TNFRSF11B*) in PA conditions. This interaction expanded with TGF‐β1 treatment to include interactions between SMC‐like cells and matured hepatocyte‐like stages. OPG expression has been suggested as an evasion mechanism from TRAIL‐induced apoptosis acquired by activated myofibroblasts in liver fibrosis (Habibie *et al*, 2021), and the expansion in signaling to SMC‐like cells through this pathway from PA to TGF‐β1 treatment suggests the gradual acquisition of fibrogenic activation in these myofibroblast‐related cells. Osteopontin (encoded by *SPP1*) can bind CD44 to regulate cell adhesion and migration (Weber *et al*, 1996). SMC‐like cells with PA and TGF‐β1 treatment induced autocrine interactions through *SPP1* and *CD44*, consistent with the behavior of activated HSCs in mouse and human MASH livers (Wang *et al*, 2023). With TGF‐β1, the SMC‐like cell‐sourced *SPP1*/*CD44* interaction further expanded within the mesenchymal targeting HSC‐like cells. TGF‐β1 treatment also broadly induced *PDGFB*/*PDGFR* interactions, indicating the acquired potential for hepatocyte‐like lineages to signal to HSC‐like cells through PDGFRA and hepatocyte‐ and DC‐like lineages to signal to HSC‐ and SMC‐like cells through PDGFRB, in line with the critical role of PDGFB‐mediated HSC‐activation in liver fibrogenesis (Kocabayoglu *et al*, 2015; Ying *et al*, 2017). We also identified less studied interactions between cell types, including TGF‐β1‐mediated *TGFB1*/*Androgen receptor (AR)* and *CD44*/*FGFR2* crosstalk between SMC‐like and hepatocyte‐like cells. Together, these findings highlight the alignment of subsequent cellular crosstalk changes from OA, to PA, to TGF‐β1‐treated HLOs to pathways involved in human MASLD progression. Our analyses further highlight the androgen‐receptor pathway as a potential molecular target in fatty acid‐induced liver injury and fibrosis.

### Trajectory inference reconstructs the emergence of major HLO lineages and injury‐specific terminal states

Progression from steatosis to steatohepatitis and then fibrosis can be understood as a sequential process that includes the emergence of myofibroblast lineages (Anstee *et al*, 2019; Kisseleva & Brenner, 2021). We investigated the projection of our steatosis (OA), steatohepatitis (PA), and fibrosis model (TGF‐β1) on a force‐directed layout in conjunction with Palantir (Setty *et al*, 2019) trajectory inference analysis to dissect this process.

**Figure 6:**
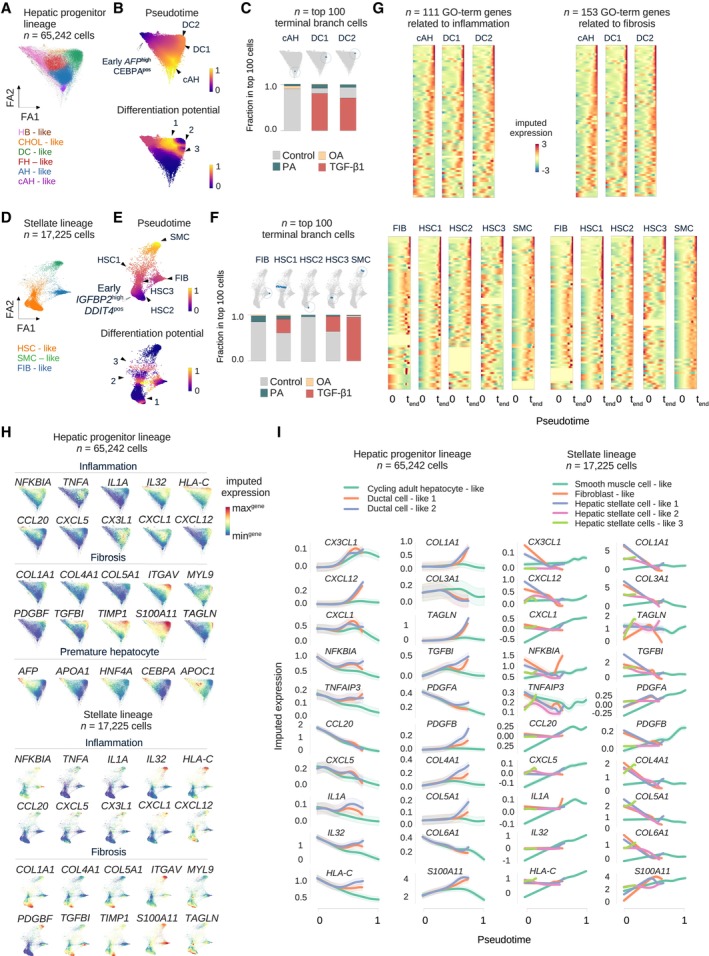
Trajectory inference reconstructs the emergence of major HLO lineages and injury‐specific terminal states AForceAtlas2 representation of the hepatic progenitor lineage composed of cells from healthy controls and OS‐HLOs treated with OA, PA, and TGF‐β1, colored by cell type. *N* = 65,242 cells from 14 replicates (*n* = 2 replicates per condition, *n* = 8 controls).BForceAtlas2 representation from (A), colored by Palantir trajectory inference results based on the ForceAtlas2 representation, showing pseudotime (top) and differentiation potential (bottom). For pseudotime, black arrowheads indicate three pseudotime maxima, and the white arrowhead identifies the early cell (selected based on the highest level of *AFP* expression among cells expressing *CEBPA*, both of the genes marking hepatocyte precursors, Wesley *et al*, 2022). Regions of high differentiation potential are indicated by arrowheads 1–3.CProjection of the top 100 terminal branch cells (sorted by branch probabilities) for each terminal state identified by Palantir on the ForceAtlas2 representation from (A) (top). Corresponding barplots below displaying the relative distribution of treatment conditions among the top 100 terminal branch cells for each terminal state.D–FDisplay the plots from (A) to (C) for the HSC lineage, in this case the early cell is selected based on the highest level of *IGFBP2* expression among cells expressing *DDIT4*, as a marker of fetal HSCs (Wesley *et al*, 2022). *N* = 17,225 cells from 14 replicates (*n* = 2 replicates per condition, *n* = 8 controls).GGene expression trends reveal the increase in inflammatory and pro‐fibrotic transcripts along the pseudotime ordering towards PA and TGF‐β1 dominated terminal states. Heatmaps showing the imputed gene expression over Palantir pseudotime for GO term (The Gene Ontology Consortium, 2000; Carbon *et al*, 2021) derived inflammation (left) and fibrosis (right) related genes sorted by their imputed expression level at each terminal state (indicated on top of each heatmap). Imputed expression levels are indicated by color, *y*‐axes correspond to genes, and *x*‐axes display the pseudotime. Ranked gene lists are provided in Dataset EV8.HForceAtlas2 representations for hepatic progenitor and HSC lineages from (A) and (D), showing the MAGIC‐imputed gene expression of selected inflammation‐ and fibrosis‐related genes from (G) enriching towards specific terminal states. Fetal hepatocyte‐related transcripts enrich along the trajectory towards the OA‐treated hepatoblast‐like cells. Projection of the imputed gene expression of transcripts related to inflammation, fibrosis, and the developmental stage of hepatoblasts is indicated by section headings. Color represents the range of imputed expression values for each gene.IGene expression trends for hepatic progenitor (left three columns) and HSC (two right columns) lineages of representative enriched genes from (G). The pseudotime is indicated on the *x*‐axes and normalized expression is shown on the *y*‐axes. Cell types are indicated by color (top) and can be identified in (A) and (D). Note that not not all terminal cells reach the maximum pseudotime value of 1 and therefore terminate their trend line before the *x*‐axis maximum (compare (B) and (E)).

We first analyzed the hepatic progenitor lineage (Fig 6A) choosing the *ALB* ^highest^/*CEBPA* ^pos^ hepatoblast‐like cell as the early cell (Fig 6B, top). As expected from the immature gene expression signatures in hepatoblast‐like cells, pseudotime increased towards the adult hepatocyte‐like cluster (Fig 6B, top). Three terminal states were identified, including the cycling adult hepatocyte‐like state and two DC‐like terminal states emerging through the cholangiocyte‐like cells (Fig 6B, top). We next identified cells with high differentiation potential (Fig 6B, bottom), showing enhanced activity in three defined regions, including one at the hepatoblast/cholangiocyte (arrowhead 1) and two at the cholangiocyte‐ductal (arrowhead 2 and 3) interface. Terminal states are defined by a single cell and do not allow us to evaluate the population of cells most closely related to the terminal state. To understand the contribution of each treatment to terminal states, we selected the top 100 terminal cells for each of the three terminal states identified and projected these cells on the force‐directed layout (Fig 6C, top). Analysis of the cell proportions revealed that the AH‐like cycling terminal state (cAH) was primarily composed of cells from the control conditions, while the DC‐like states were dominated by TGF‐β1‐treated cells (Fig 6C, bottom). Performing a similar analysis on the HSC lineage (Fig 6D), choosing the *IGFBP* ^highest^/*DDIT4* ^pos^ cell as the early cell, revealed five terminal states in the regions of three HSC‐like clusters (referred to as HSC1‐3), the fibroblast‐like cluster (FIB), and one SMC‐like cluster (Fig 6E, top). The three regions of high differentiation potential corresponded to the interfaces between these major mesenchymal cell types (Fig 6E, bottom, arrowheads 1–3). Analysis of the cell proportions revealed that the FIB and HSC2 terminal states were mostly represented by cells from the control condition, while the HSC1 and HSC3 were primarily composed of cells from control and TGF‐β1‐treated cells. In contrast, SMC‐terminal state was largely represented by TGF‐β1‐treated cells (Fig 6F, bottom). Together, these analyses suggest that only TGF‐β1‐treatment favored the emergence of a myofibroblast‐like terminal state, consistent with a canonical feature of fibrosis pathology (Kisseleva & Brenner, 2021).

To examine the differentiating regions as potential representations of healthy‐to‐injury transitions, we next applied MAGIC (van Dijk *et al*, 2018) to generate imputed data and visualize the expression of relevant genes along the differentiation trajectory with a focus on the acquisition of “pro‐fibrotic” and “pro‐inflammatory” gene expression signatures. We selected genes based on GO terms (The Gene Ontology Consortium, 2000) and sorted the genes according to their imputed expression at the final pseudotime to visualize whether these signatures were enriched towards each terminal state (Fig 6G, Appendix Fig S7, Dataset EV8). For the hepatic progenitor lineage, this analysis revealed an increase in pro‐fibrotic gene signature enrichment across pseudotime for cAH‐ and DC‐like terminal states (Fig 6G, top). In the HSC‐like lineage, the SMC‐like terminal state enriched a broad spectrum of pro‐inflammatory and pro‐fibrotic gene signatures along pseudotime, with variable enrichment for HSC‐like and fibroblast‐like cells (Fig 6G, bottom). Interestingly, OA‐treated cells were hardly represented across the cells constituting the extrema of trajectories leading to terminal states with fibrotic signatures.

To resolve transcripts specific to each terminal state, we projected the imputed expression of genes enriched in the above analyses or related to hepatocyte precursor states on the force directed layout for each lineage (Fig 6H). In the hepatic progenitor lineage, inflammatory transcripts *NFKBIA*, *CXCL6* and *CXCL1* increased towards the TGF‐β1‐dominated DC‐like terminal state. We observed a targeted diffusion distribution of pro‐fibrotic genes towards the same cells, again corresponding to the TGF‐β1 treatment enriched trajectory (e.g., *TGFBI* and *TIMP1*). The hepatoblast‐like terminal state (that expanded under OA treatment, Fig 4B) showed enrichment for hepatocyte precursor defining transcripts *AFP*, *APOA1*, *APOC1*, *HNF4A,* and *CEBPA*, providing further evidence that OA favors the expansion of hepatocyte precursor states. Profiling of the HSC‐related lineages revealed *IL32* and *HLA‐C* as components of the inflammatory, and *ITGAV* and *S100A11* as components of the fibrotic signature towards the SMC‐like terminal state, which was overrepresented by TGF‐β1 treated cells. These transcripts are also characteristic of activated HSCs and a hallmark of fibrosis *in vivo* (Henderson *et al*, 2013; Baselli *et al*, 2020), suggesting the emerging SMC terminal state represents the activation of these HSC‐like cells.

To better understand gene expression signatures accounting for deviating terminal states observed in injured HLOs, we next projected lineage‐specific gene expression trends over pseudotime (Fig 6I). This highlighted pro‐fibrotic and pro‐inflammatory genes characterizing individual terminal states of the hepatic progenitor lineage, which were the cAH‐ and DC‐like states (Fig 6I, two left columns; green, orange, and blue lines, respectively). In the HSC‐related lineages (Fig 6I, two right columns), inflammatory signatures accompanied the trajectory towards the TGF‐β1‐specific SMC‐like terminal state (green line), along with fibrosis‐related genes such as *PDGFB*, *S100A11* and *TAGLN*. The pseudotime is indicated on the *x*‐axis, with a value of 1 representing the greatest pseudotime. Some cell populations terminate before 1 based on pseudotime calculations for each terminal cell state. Together, these results support the presence of inflammatory and fibrotic signatures on the path towards fibrosis being modeled by PA and TGF‐β1 treatments, and underscore the induction of premature hepatocyte stages by OA.

### Gene signature scores predicting MASLD severity progressively increase from PA to TGF‐β1, and show a mixed response with OA

**Figure 7:**
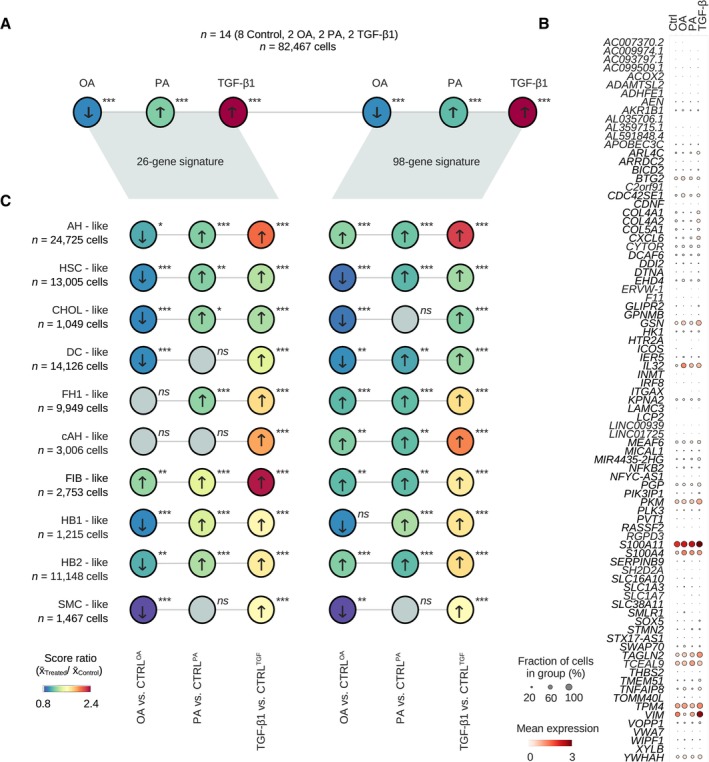
Gene signature scores predicting MASLD severity progressively increase from PA to TGF‐β1, and show a mixed response with OA Application of 26 and 98 gene signatures (Pantano *et al*, 2021) to predict fibrosis stages across MASLD to control and injured OS‐HLOs. Dot plot showing the ratio of the mean gene signature score in treatments and their respective controls. *P*‐values derived from pairwise Mann–Whitney‐*U*‐statistics (two‐tailed) comparing scores across all cells in treatment vs. their respective control condition. *P ≤ 0.05; **P ≤ 0.01; ***P ≤ 0.001; ns, non‐significant. All *P*‐values are provided in the source data of the fig *N* = 82,467 cells from 14 replicates (*n* = 2 replicates per condition, *n* = 8 controls).Dotplot shows the scaled mean expression of commonly expressed genes from the 98‐signature score across treatment conditions. Dot sizes correspond to the percentage of cells expressing the respective gene in each condition, and the level of expression is indicated by color.Deconvolution of the scores from (A) to individual cell types as annotated in Fig 4B. Dot plot showing the ratio of the mean gene signature score in treatments and their respective controls. *P*‐values derived from pairwise Mann–Whitney‐*U*‐statistics (two‐tailed) comparing scores across all cells of the indicated cell type in treatment vs. their respective control condition. *P ≤ 0.05; **P ≤ 0.01; ***P ≤ 0.001; ns, non‐significant. All *P*‐values are provided in the source data for the figure. *N* numbers are indicated below cell cluster names. Source data are available online for this figure.

We next investigated how changes in gene expression following treatment of HLOs with OA, PA, and TGF‐β1 relate to the development of fibrosis in patients with MASLD. We applied the 26 and 98 gene signatures established to predict fibrosis in MASLD (Pantano *et al*, 2021) to bulk gene expression in HLOs. This analysis revealed a significant increase in the scores with TGF‐β1 and PA treatment for both gene signatures, with the mean score increasing to the greatest extent with TGF‐β1 treatment, followed by PA (Fig 7A). We then plotted expression signatures for individual genes that demonstrated the greatest dynamic range in expression across clusters (Fig 7B). Many of these genes show a trend of induction from control, to OA, to PA, to TGF‐β1, including *TAGLN2*, *CXCL6*, *CYTOR*, and *S100A11*, while other genes, such as *S100A4* show the highest expression with OA treatment. These results demonstrate a stepwise induction of MASLD disease progression scores with PA and TGF‐β1, and a mixed response with OA in HLOs.

We then evaluated the 26 and 98 gene signatures at the level of cell types. The 26 and 98 gene signatures increased across the major cell types of adult hepatocyte‐, HSC‐, and cholangiocyte‐like cells with significance in at least one of the two signature scores for PA and TGF‐β1 treatment (Fig 7C). In contrast, we observed a reduction in both 26 and 98 gene signatures with OA treatment for adult hepatocyte, HSC‐ and cholangiocyte‐like cells. The main contributors to the OA‐related MASLD signature were hepatocyte‐like populations within the 98 gene signature. To further dissect the contribution of individual cell types to the score enrichment, we next plotted the relative expression of individual genes per cell cluster across treatment conditions (Appendix Fig S8). Adult hepatocyte‐like cells in HLOs treated with TGF‐β1 showed higher expression of *S100A11*, while OA and PA induced *TCEAL9* in this population. HSC‐like cells showed induction of *COL4A1*, *COL4A2*, *COL5A1*, *PKM*, *VIM*, and *TPM4* with TGF‐β1 treatment, while OA and PA treatment resulted in increased expression of the inflammatory signature gene *IL32* in both epithelial and mesenchymal clusters. Together, these results indicate the gradual acquisition of gene signatures linked to disease progression in MASLD across the major HLO cell types with PA and TGF‐β1, additionally resolving a mixed response in the OA condition, where hepatocyte precursors were the main drivers of gene expression changes observed with MASLD progression, while AH‐, cholangiocyte‐, and HSC‐like populations lost the MASLD fibrosis signature.

## Discussion

While studies have begun to decipher the contribution of individual cell types to the development of cirrhosis in humans (Ramachandran *et al*, 2019), our understanding of gene expression programs and the interaction between cell types during evolving liver fibrosis in the context of MASLD is limited. Conventional 2D cultures fail to provide cellular complexity, and animal models come with ethical constraints and do not involve human cells. Recent multi‐lineage *in vitro* organoid systems, such as human liver organoids (HLOs), hold the promise to bridge the gap between cell culture and clinical observations (Sharma *et al*, 2020). In HLOs, MASLD and fibrosis have been studied so far by oleic acid (OA) treatment (Ouchi *et al*, 2019) and genetic manipulation (Guan *et al*, 2021). However, saturated FFAs, like 16:0 palmitic acid (PA), show a tighter association with MASLD (Hliwa *et al*, 2021) and more efficiently induce hepatocyte damage (Ricchi *et al*, 2009; Moravcová *et al*, 2015). Despite the critical importance of choosing the most suitable HLO‐MASLD model, a systematic evaluation of the efficacy on MASLD transcriptome induction among lipotoxic agents in HLOs has not yet been performed.

Here, we examined the effect of OA‐, PA‐, and TGF‐β1‐mediated injury in HLOs and the ability of each condition to model the progression of steatohepatitis and fibrosis caused by MASLD‐induced liver disease. This analysis covered histologic, phenotypic, and gene expression studies, including the creation of a reference of ~ 100,000 single‐cell transcriptomes of injured and healthy MASLD‐HLOs. We find that OA induces patterns of gene expression that are more reflective of benign steatosis, while the inflammatory changes of steatohepatitis are induced with PA, and the combination of steatohepatitis and fibrosis is most prominent with TGF‐β1.

While OA induces steatosis, inflammatory changes were only observed in hepatocyte precursors. Unexpectedly, OA treatment not only fails to induce *COL1A1* mRNA measured by qRT‐PCR and reduces intra‐organoidal collagen levels as quantified by Sirius red stainings, but it also significantly ameliorates fibrotic gene signatures across cell types and largely reduces the HSC population in HLOs. In contrast, PA induces a more robust steatohepatitis signature from more mature hepatocyte sub‐populations, while having minimal effect on HSC population size or gene expression. TGF‐β1 is the only agent tested that generated a global fibrotic phenotype including collagen type 1 induction. Additionally, only TGF‐β1 treatment results in the expansion of the *KRT19*/*HNF4A*+ DC‐like population, consistent with the reaction observed with chronic liver injury *in vivo* (Español‐Suñer *et al*, 2012; Raven *et al*, 2017; Deng *et al*, 2018; Sato *et al*, 2019; Pu *et al*, 2023). It is not clear why previous studies in HLOs (Ouchi *et al*, 2019) show a more marked fibrotic change with OA treatment, but it may reflect differences in culture conditions or the presence of a small number of Kupffer‐like cells, which we do not observe in our HLOs. However, the conclusion that PA is a more effective model for steatohepatitis in HLOs is consistent with both human and mouse studies.

OA, a monounsaturated fatty acid, is the major component of olive oil (Yubero‐Serrano *et al*, 2019). Olive oil is an integral component of the Mediterranean diet (MD) (Dinu *et al*, 2018), and growing evidence over the past decades has demonstrated health benefits from the MD, attributed to its high olive oil content (Dinu *et al*, 2018). The beneficial effects on cardiovascular disease are well established (Estruch *et al*, 2018; Delgado‐Lista *et al*, 2022), and a recent meta‐analysis of randomized controlled trials highlights the positive effects of the MD in MASLD, with the observation of reduced ALT levels and liver stiffness (Haigh *et al*, 2022). *In vivo* studies in mice and rats have also demonstrated that OA protects against inflammatory and fibrotic effects in models of MASH (Lee *et al*, 2011; Chen *et al*, 2018; Zeng *et al*, 2020). The observation that OA reduces the fraction of HSCs and induces an almost pan‐cellular reduction of fibrotic signatures in HLOs could help explain beneficial effects observed in these studies.

Differential effects have been observed for OA and PA *in vitro* that also indicate OA could reduce fibrosis. While OA and PA induce steatosis in hepatocytes, and OA is observed to have a more pronounced effect on steatosis than PA in some studies and similar effects in other studies (Müller & Sturla, 2019), PA more potently induces cytotoxicity and apoptosis compared to OA (Ricchi *et al*, 2009; Moravcová *et al*, 2015). Additionally, evidence for OA‐mediated protection from lipotoxicity in cultured hepatocytes has been observed (Ricchi *et al*, 2009). Treatment of HSCs directly with OA leads to reduced collagen, ɑSMA, and vimentin levels and inhibits proliferation (Hetherington *et al*, 2016; Hong *et al*, 2018). In contrast, PA has been linked more directly to fibrosis, as conditioned media from PA‐treated hepatocytes induces fibrotic gene expression in HSCs (Piras *et al*, 2020).

Our study does come with limitations. First, in their current state, HLO systems do not fully mimic the cellular landscape found *in vivo*. This is evident in alternate cell type proportions in HLOs compared to the human liver, such as the relative abundance of cholangiocyte‐like cells. HSC‐like cells consistently range at higher‐than‐physiological fractions in HLOs (Ouchi *et al*, 2019). We addressed this limitation through the relative reduction of HSC‐like and cholangiocyte‐like cells with the OS culture method, resulting in a relative expansion of hepatoblast‐ and hepatocyte‐like fractions. Adult hepatocyte‐like cells are also detected in OS conditions with expression profiles consistent with zonation, but with an under‐representation of pericentral hepatocytes. While HSC‐ and cholangiocyte‐like cells decrease in OS conditions, they remain more frequent than observed in the liver, where both cell types comprise about 5% of cells (Yin *et al*, 2013; Banales *et al*, 2019). Additionally, immune cells are a critical component in MASLD development, and in our HLO system we do not detect Kupffer cells previously reported (Ouchi *et al*, 2019), which is potentially a result of heterogeneity in cell type annotation strategies of rare populations. Second, our analysis still revealed proportions of premature cell stages, particularly in the hepatocyte‐like fraction. Third, inter‐batch variability is present in HLO culture, which is why in this and future studies the differentiation of appropriate controls along with treatment samples in each generation of HLOs is strictly required. We do observe a larger fraction of cholangiocyte‐like cells compared to previous reports (Ouchi *et al*, 2019), but this is likely to reflect discrepancies in different cell type annotation strategies more than inter‐sample variability. Fourth, replicates were limited (*n* = 2 technical scRNA‐seq replicates), which may have reduced the power to detect small changes between groups. Additionally, our initial observations of size shifts in Matrigel‐cultured HLOs (Fig 3B) may potentially be associated with changes in transcriptomic phenotypes, which we did not investigate due to our initial focus on expression of collagen and *TNFA* as markers of fibrosis and inflammation in MASLD. Together, further studies will be necessary to (i) develop HLO models of greater cell type variety and maturity, (ii) reduced inter‐batch variability, and (iii) continuously evaluate their capacity to model human pathologies.

In summary, we herein provide a systematic approach to benchmark MASLD‐HLOs, including single‐cell analysis, which will serve as a reference atlas for future studies. The interplay between inflamed, steatotic hepatocytes, cholangiocytes, and HSCs provided in the HLOs demonstrate the antifibrotic effects of OA observed *in vivo*, and suggest they are driven at least in part by a reduction of HSCs. This may be an underexplored mechanism by which OA exerts beneficial effects in animal models and MASLD patients. Our results also provide evidence for the rationale to favor PA over OA to model steatohepatitis in HLOs and to use TGF‐β1 to study the combination of inflammation and fibrosis observed in later‐stage disease. In the future, such model systems could be used to predict the differential outcomes on MASH and fibrosis of drug candidates prior to clinical trials.

## Materials and Methods

### hPSC and HLO culture

H1 hPSC cells (WA01) were obtained from WiCell. ESCRO approval was received from Massachusetts General Hospital (MGH). The iPSC4 line was generated in collaboration with the Harvard Stem Cell Institute from nucleated blood cells from a male donor in their forties. Blood was collected after informed consent and in accordance with the MGH Institutional Review Board Approval (Protocol: 2009P002847). The iPSC4 line was generated through approaches as previously described (Park *et al*, 2008; Muratore *et al*, 2014) using cDNAs for Oct4, Sox2, Klf4, and Myc delivered with Sendai Virus. Karyotyping and fingerprinting analysis were performed for iPSC4. hPSCs and hiPSCs were maintained in mTeSR medium (StemCell Technologies) on Matrigel (Corning, 354230) as previously described (Daneshvar *et al*, 2016) and split with Accutase (Thermo Fisher Scientific) upon 80–90% confluence. For passage and thawing, Rho‐associated kinase (ROCK)‐Inhibitor (StemCell Technologies, Y‐27632) was added to mTeSR at 10 μM. For the differentiation of human liver organoids (HLOs), cells were split at a ratio of 1:10 and cultured until ~ 85% confluent before HLOs were differentiated as previously described (Ouchi *et al*, 2019; Thompson & Takebe, 2020). On day 21, HLOs were either kept in Matrigel domes or isolated. To perform isolation, HLOs were incubated in DPBS(−/−) on ice for 15 min followed by repeated manual dissociation with a P1000 at 4°C followed by centrifugation at 290 *g* for 3 min, removal of supernatant, and resuspension in 10 μl final medium per receiving well of a 24‐well plate. Isolated HLOs in media were either plated to (i) 1% agarose‐coated plates, (ii) plates on an orbital shaker at 80 revolutions per minute, or iii) hydrogel plastics ultra low attachment surface 24‐well plates (Corning, 3473).

### Liver injury induction

Injury media solutions based on Hepatocyte Culture Medium (HCM, Lonza, CC‐3198) were prepared to obtain solutions of TGF‐β1 (R&D Systems 240‐B‐002), palmitic acid (PA, Sigma Aldrich P0500), and oleic acid (OA, Sigma Aldrich O1383). HLO media was changed to injury media on day 21. Isolated HLOs were resuspended in the final injury media at the final step of the isolation process (see above) and kept in solution for 4 days and harvested on day 25. Controls for TGF‐β1 were cultured in HCM. PA was dissolved into HCM with 10% BSA and 1% ethanol before dilution to final concentration in HCM. OA was dissolved into DPBS(−/−) with 12.5 mM NaOH and 1.67% BSA at 8 mM before dilution to final concentration in HCM. PA and OA controls were prepared accordingly, omitting the initial step of dissolving the active agent in the carrier solutions.

### Contraction assay

Day 20–21 HLOs in 50 μl Matrigel domes cultured in HCM were exposed to TGF‐β1 at 10 and 25 ng/ml. Images of plates with scale bars were taken prior to treatments and on day 5. Measurements were performed with ImageJ (version 1.53a) by manually selecting the HLO/matrix drop areas. All distances in mm^2^ were normalized to the mean of the control areas in mm^2^, and two‐tailed Kruskal–Wallis‐statistics were performed on all groups with a Conover *post hoc* test.

### qRT‐PCR

Total RNA was isolated from HLOs to perform qPCR. Briefly, 1 ml medium was removed from 6 well plates. Matrigel domes containing HLOs were carefully detached from the wells with a cell scraper and transferred to a 1.5 ml tube including medium. If isolated HLOs served as starting material, the detachment step was not needed. Matrigel‐embedded HLOs were left at 4°C for 15 min to dissolve the Matrigel. Then, HLOs were centrifuged for 3 min at 290 *g* at 4°C (Matrigel‐embedded HLOs) or at RT (non‐matrigel HLOs or 2D cell layers). Supernatant was discarded, and the pellet was resuspended in 300 μl Trizol (Invitrogen, 15596026), rigorously vortexed or mechanically processed until fully dissociated, and finally incubated for 10 min at room temperature and then stored at −80°C. RNA was prepared via phenol‐chloroform extraction. For cDNA synthesis, 200 or 500 ng RNA were reverse‐transcribed using the iScript gDNA Clear cDNA Synthesis Kit (Biorad, 1725034), and no‐RT‐controls and mastermix controls were prepared. qPCR reactions were prepared from cDNA at 1:5 dilution with SYBR Green iTaq Universal SYBR Green Supermix (Biorad, 1725120) and qPCR primers at 10 μM in a total volume of 10 μl in a ≥ 40‐cycle and melt curve reaction cycler (Biorad, Base #CT009383, Optical Head #786BR2648). All biological samples were measured in technical triplicates.

### qRT‐PCR data analysis

All gene *C*_t_ means were calculated from three technical replicates. Only samples with housekeeping *C*_t_ values below 35 were considered for analysis. Mean *C*_t_ values for housekeeping genes β‐Actin (*ACTB*) or glyceraldehyde‐3‐phosphate dehydrogenase (*GAPDH*) were subtracted from target gene means to generate the Δ*C*_t_ value. The control Δ*C*_t_ value average was calculated from three biological replicates and subtracted from all experimental Δ*C*_t_s to render ΔΔ*C*_t_ values. The log2 fold‐change was calculated as $2^{-\Delta \Delta C_{t}}$.

### Primer design and validation

Primers were designed using PrimerBlast. Briefly, transcripts of interest were selected by NCBI Reference Sequence ID and the ‘pick primers’ hyperlink function was used to import the cDNA sequence into the PrimerBlast mask. The following settings varied from the defaults: Exon junction span → primer must span an exon‐exon junction, PCR‐product size → 50–200, allowing primers to bind to variant transcripts of the same gene. Blast was performed against both, RefSeq mRNA (Homo sapiens) and RefSeq representative genomes (Homo sapiens) in order to obtain primers specific to transcript sequence and mRNA rather than gDNA. Primers with (i) no additional match in RefSeq mRNA or at least > 4 mismatches and (ii) no additional genomic hits or hits with at least > 800 bp product size, were selected for further validation. Next, primers were tested on human liver tissue RNA at a final concentration of 10 μM as described above, and 2% agarose gels of the primer product were prepared with a 100 bp ladder. Primers were selected for further experiments if they showed a single band in the expected size range. The band was cut, cDNA was extracted from agarose and sent for Sanger sequencing, and the product identity was assured by re‐blasting. A primer was selected for experiments when the sequence matched the selected sequence of the initially selected transcripts (NCBI BLAST). Primer sequences are provided in Dataset EV9.

### Triglyceride assay

HLOs treated with OA/PA/TGF‐ꞵ1 or control solutions were collected, washed twice with DPBS(−/−), and homogenized in glycerol lysis buffer. Triglyceride quantification was performed using the enzymatic assay Triglyceride‐GloTM Assay (J3160, Promega) following the manufacturer's instructions.

### Bodipy staining

After being cultured with OA/PA/TGF‐ꞵ1 or control solutions, HLOs were collected and washed with DPBS(−/−). Lipid droplets and nuclei were stained with Bodipy 493/503 (0.5 μg/ml, D3922, ThermoFisher) and Hoechst (5 μg/ml, H1399, Invitrogen) for 40 min at 37°C. The stained HLOs were visualized and imaged with the Leica DMi8 automated Microscope (Leica Systems) using the 10× objective. All image analysis was performed using the ImageJ‐Fiji software.

### Histology and immunohistochemistry

Medium was removed from day 16, day 21, or day 25–26 HLOs treated for 4 days with pro‐fibrotic substances, washed twice with warm DPBS(−/−), and fixed overnight at 4°C with 4% PFA in DPBS(−/−). PFA was removed and the HLOs were washed 2× with DPBS(−/−) before being transferred to 70% ethanol for storage and paraffin embedding. H&E and Sirius red staining was performed on cut and deparaffinized HLOs in line with standard protocols. Anti‐human‐CEBPα (Sigma Aldrich, HPA052734) and the secondary antibody (HPR anti‐mouse) were used at a dilution of 1:200.

### Organoid Sirius red quantification pipeline

The pipeline for HLO‐specific Sirius red staining analysis is written in python (≥ version 3), ImageJ (version 1.53a) macro, and bash and is publicly available at https://github.com/anjahess/organoid‐sirius‐red. First, full slides were cleared from black artifacts. Then, for whole slide quantification, all image areas containing tissue were selected and saved for quantification. The Sirius red quantification module was adopted from the NIH‐Image J macro “Quantifying Stained Liver Tissue” (available at https://imagej.nih.gov/ij/docs/examples/stained‐sections/index.html, requested 2020‐11‐14). Briefly, RGB stacks were generated from images, and the tissue containing area was defined and measured by the *setAutoThreshold(“Default stack”)* function. The Sirius red staining threshold reached from minimum to maximum as retrieved from the *setAutoThreshold* and *getThreshold(min*, *max)* functions measured in the green channel of the RGB stack, and divided by an experiment‐specific brightness factor (0.95 or 1.3, similar across all control and treatment conditions). For single HLO analysis, 200 × 200 μm selection squares were placed around HLOs in the interactive, user‐supervised mode and resulting images were processed as described for whole slide scans. Finally, csv‐formatted result tables were called from python (version 3.7) and the Sirius red stained area was calculated either per total area or per tissue (measures as the auto‐thresholded area in the blue channel). For vacuole quantification, the fraction of stain‐free tissue was compared. If normalization was performed, values were calculated as the percentage of mean of all control samples of the respective experiment.

### Single‐cell RNA sequencing

For day 21 ULA HLO single‐cell RNA sequencing, HLOs underwent a passage step at day 16 and remained in HCM medium with 10% Matrigel on ultra‐low attachment plates as previously described (Thompson & Takebe, 2020). At day 21, one well of a six well plate of free‐floating HLOs was collected, washed 1× with warm DPBS(−/−) and briefly dissociated by incubation with 300 μl 0.05–0.25% Trypsin–EDTA for 10 min at 37°C (Corning, Ref. 25‐052‐CI RT). Day 25 orbital shaker‐cultured HLOs underwent an additional DBPS (−/−) wash and 10 min of trypsinization to allow complete HLO digestion. For each replicate, full single‐cell dissociation was confirmed manually by light microscopy. After a final DPBS(−/−) wash, HLOs were resuspended in DPBS(−/−), counted after Trypan blue staining with the TC20™ Automated Cell Counter according to a previously determined optimal gating of 7–20 μm and transferred on ice. Library preparation was performed on biological replicates with the highest viability counts. Libraries were prepared according to the 10× ChromiumSingle Cell 3′Reagent Kits v3 instructions. The library was sequenced by paired‐end sequencing on an Illumina® NextSeq 2000 P3 flow cell or the NovaSeq 6000 system according to the manufacturer's recommendations.

### Single‐cell RNA sequencing analysis

#### Alignment and quality controls (QCs)

Raw bcl files were converted to fastq by the command *bcl2fastq ‐‐use‐bases‐mask Y26*,*I8*,*Y98*. Fastq quality was assessed with multiqc (Ewels *et al*, 2016) (version 1.9). Fastq files were aligned to the GRCh38 genome with *cellranger count* (=> version 3.0.2). Doublet scores were calculated with Scrublet (version 0.2.3) (Wolock *et al*, 2019) with default settings on the cellranger filtered count matrices. Cells with doublet scores below 0.5 were accepted for downstream analysis performed with scanpy (Wolf *et al*, 2018) (version 1.7.2, functions abbreviated with *sc* from here on) in python (version 3.7). All replicates were loaded and merged using the *sc*.*concatenate* function.

#### Cell QC

Violin and scatter plots of QC parameter distributions were manually inspected for all samples and the following joint criteria were applied: (i) at least 500 genes per cell, (ii) a maximum mitochondrial gene fraction of 20%, (iii) a maximum ribosomal gene fraction of 40%, and (iv) a minimum of 500 and a maximum of 30,000 counts. Transcripts were accepted if present in at least 10 cells. Prior to QC, the dataset contained a total of 21,067 cells (ULA‐HLOs), and 99,119 cells (OS‐HLOs). After QC, the dataset contained 16,835 cells (ULA‐HLOs), and 82,467 cells (OS‐HLOs, Dataset EV10). All replicates were jointly total‐count normalized excluding the top 10% highly expressed genes, logarithmized (*X* = log(*X* + 1), natural logarithm). Highly variable genes were selected using the sc.pp.highly_variable_genes function with the n_top_genes parameter set to 5,000. Each gene was transformed to unit variance by applying the *sc.pp.scale* function with the *max_value* parameter set to 5.

### Cell cycle scoring

Cell cycle scores (S‐, G2M‐Score) and cell cycle phase (S, G2M, G1) were assigned based on a previously published cell cycle defining gene list (Tirosh *et al*, 2016) with the *sc.score_genes_cell_cycle* function.

### Dimensionality reduction, embedding, clustering

Normalized, log‐transformed, and scaled data were objected to calculation of principal component analysis (PCA) coordinates, loadings, and variance via *sc.tl.pca*. The neighborhood graph was calculated using the first 50 principal components and embedded utilizing the Uniform Manifold Approximation and Projection (UMAP) (McInnes *et al*, 2018) algorithm via *sc.api.tl.umap*. Clusters were identified with the Leiden algorithm (Traag *et al*, 2019) at resolution 0.1. Coarsenesses lower than the default parameters were chosen to reproduce approximately five cell types experimentally validated in HLOs (Ouchi *et al*, 2019) and based on optimal performance in cluster robustness metrics, as evaluated through calculation of Davies‐Bouldin indices and Silhouette scores in sklearn (Pedregosa *et al*, 2011) calling the *sklearn.metrics.silhouette_score* and *sklearn.metrics.davies_bouldin_score* functions as previously described (Fast *et al*, 2021) (Appendix Fig S2g). Clusters were required to represent all individual control replicates, and if this criterion was not met the clusters were excluded from the downstream analysis (led to the exclusion of 717 cells, Dataset EV10). The force‐directed graph was computed via *sc.tl.daw_graph* implementing ForceAtlas2 (Jacomy *et al*, 2014, 2) with default parameters.

### Cluster marker gene identification and pathway enrichment analysis

Cluster‐characterizing genes were defined using scanpy's implementation of the Wilcoxon rank‐sum test (comparing each cluster against all other clusters) followed by Benjamini–Hochberg correction (*sc.tl.rank_genes_group*).

### Batch correction and force‐directed graph drawing

Batch effects were corrected with the scanpy implementation of Harmony (Korsunsky *et al*, 2019) applying the *sce.pp.harmony_integrate* function followed by recalculation of the neighborhood graph and UMAP based on the Harmony representation. Convergence was reached prior to the maximum of 10 iterations.

### Cell cluster annotation

#### Automated cluster annotation based on literature markers

Literature research was performed to assemble a set of specific marker genes for cell types potentially emerging in HLOs, including mature liver cells and progenitors (Dataset EV1). The top 200 Wilcoxon rank sum test derived marker genes of each cluster, based on control conditions, were forwarded to the *gseapy.enrichr* (Kuleshov *et al*, 2016; Fang *et al*, 2022) function together with the literature reference list, the number of genes in the dataset was provided as the background parameter. Results from *enrichr* with an adjusted *P*‐value below 0.05 were assigned as the new cluster identity based on the number of significantly enriched marker genes. If *P*‐value criteria were not met, the label with the lowest adjusted *P*‐value and maximum number of matching genes was chosen as the identity. Accepting the label “Embryonic stem cells “required expression of either *SOX2*, *NANOG*, *POU5F1* or *KLF1*.

#### Automated annotation based on curated databases

The ScType (Ianevski *et al*, 2022) marker gene set (accessed 2022‐Nov‐25, https://sctype.app/database.php) was used as a reference for the statistical enrichment described above. Cholangiocyte‐like cells were further sub‐clustered and annotated as described. The ductal cell‐like cluster 2 was initially annotated as “Stromal cells” (*P* > 0.05, not significant) and was manually merged into the ductal cell‐like cluster after inspection of marker gene expression and euclidean distance dendrograms (Appendix Fig S4d). Hepatocyte‐like cells were sub‐clustered and annotated to previously published marker genes from a single‐cell atlas of human liver development (Wesley *et al*, 2022) (pairwise hepatocyte DGE from “Source Data Fig. 1”, accessed 2022‐Dec‐10: https://static‐content.springer.com/esm/art%3A10.1038%2Fs41556‐022‐00989‐7/MediaObjects/41556_2022_989_MOESM4_ESM.xlsx, sheet “Hepatocyte_pairwise DEG”). Genes with a negative log fold change in HB2 compared to HB1 were used as HB1 marker genes since no HB1‐specific pairwise genes with a positive log fold change were available, for all other hepatocyte stages genes with a positive log fold change compared to their respective precursor state were selected as marker genes, and cells were annotated as described above.

#### Cell‐type‐specific pathway enrichment analysis

For each cell type the top 600–700 genes identified by the Wilcoxon rank sum test were forwarded to EnrichR (Kuleshov *et al*, 2016)/gseapy (Fang *et al*, 2022) (version 0.10.7, “WikiPathways_2021_Human” gene set, organism “Human”).

### Hepatocyte zonation score

Marker genes for six available adult human hepatocyte clusters were retrieved from adult human liver scRNAseq data (Wesley *et al*, 2022) (cluster DGE from “Source Data Fig. 1”, accessed 2022‐Dec‐10, https://static‐content.springer.com/esm/art%3A10.1038%2Fs41556‐022‐00989‐7/MediaObjects/41556_2022_989_MOESM4_ESM.xlsx), reduced to the top 100 genes per cluster and sub‐clustered cells were annotated as described above. Next, each hepatocyte cluster was assigned an integer between 0 and 5 (Periportal‐C5:0, Periportal‐C14:1, Periportal‐C6:2, Interzonal‐C15:3, Pericentral‐C1:4, Pericentral‐C3:5) reflecting the position of the cluster on the periportal‐to‐pericentral spectrum defined by the authors of the reference study (Wesley *et al*, 2022), and this value constituted the zonation score.

### CellTypist annotation

#### Generation of a reference AnnData object

For comparing HLO cells to human liver cells, human 10× scRNA‐seq data were chosen as a reference (Wesley *et al*, 2022). H5ad files for each cell type were downloaded from the online portal (https://collections.cellatlas.io/liver‐development) and concatenated in scanpy to create a composite *AnnData* object and normalized to 10,000 cells to align with the CellTypist input requirements.

#### CellTypist classification of HLO‐cell types based on human reference data

Using CellTypist (version 1.5.0), the created *AnnData* object was defined as the reference dataset. A classifier was trained with the top 100 genes, the “cell” slot in the adata.obs slot as the reference cluster identifier and feature selection enabled. Subsequently, the HLO object was normalized to 10,000 cells to align with the CellTypist input requirements and classified in the probability match mode and a minimum probability of 0.1.

### Inflammatory gene and fibrosis scoring

To render gene lists for biological processes related to fibrotic and inflammatory injury scenarios, publicly available GO term (The Gene Ontology Consortium, 2000) gene lists were browsed via AmiGO2 (Carbon *et al*, 2009, 2) filtering for Homo sapiens genes only and the *“bioentity_label”* column was exported. All GO terms are listed in Dataset EV5. Duplicate values, and genes not present in the *adata.var* slot were removed from the lists prior to scoring Finally, *sc.tl.score_genes* with the number of control genes set to the size of the input gene list was used for scoring cells.

### DGE and pathway enrichment analysis

#### Differential gene expression (DGE)

DGE was performed on raw count data for each cluster separately in pairwise comparisons between treatments and respective controls (e.g. Control^TGF‐β1^ vs. TGF‐β1) applying the Wilcoxon rank‐sum test after exclusion of mitochondrial and ribosomal genes.

#### Pathway enrichment analysis

For each cluster, a hypergeometric enrichment test was performed on the top 200 differentially expressed genes using EnrichR (Kuleshov *et al*, 2016)/gseapy (Fang *et al*, 2022) (version 0.10.7, “WikiPathways_2021_Human” gene set, organism “Human”).

### CellPhoneDB cell–cell interactome

CellPhoneDB (Efremova *et al*, 2020) (version 2.1.7) with rpy2 (version 3.0.5) was run on *h5ad* files containing log‐transformed count data to infer cell–cell interactions. Metadata from previously harmony‐integrated and cell‐type‐annotated *AnnData* objects served as the input to the *statistical_analysis* function along with “gene_name” set as an identifier for genes in the counts data. To generate the count network data and inputs for dotplots and heatmaps, *plot_heatmap_plot* and *plot_dotplot* functions were applied, and plots were created in python (version 3.7). The fraction of total interactions was calculated by dividing each cell–cell interaction count by the sum of all interactions in the respective condition group. The delta value of interactions between treatment and control condition was calculated by subtracting the fraction for each cell–cell interaction in the treatment group by the corresponding fraction of the cell–cell interaction in the control group. Clustered heat maps with euclidean distance dendrograms were created using seaborn (Waskom, 2021) based on either the delta of interactions or the total interaction count. Chord diagrams were computed with the python packages holoviews (version 1.15.3) and bokeh (version 3.0.3) based on the delta of interactions and computed separately for interactions with a positive delta value (labeled “enforced”) or a negative delta value (labeled “reduced”) with respect to the control condition. Mean expression indicated by dot size in all dot plots refers to CellPhoneDB output “means”, and is the mean of the respective two individual partners' average expression. Venn diagrams displaying overlapping and distinct receptor‐ligand interactions, the CellPhoneDB output file “pvalues” served as an input. The list was filtered for significant interactions (*P*‐value < 0.05), and each interaction received an identifier for its cell–cell‐pair and receptor‐ligand pair (e.g. “NRP1_VEGFA_Cholangiocytes|Hepatic stellate cells”). Next, the overlapping and distinct fractions were calculated based on lists of identifiers found in each treatment condition (Dataset EV7), and UpSet plots were generated (Lex *et al*, 2014). For representing induced receptor‐ligand pairs the set was filtered to receptor‐ligand pairs with an increase of at least two significant cell–cell pairs in the treatment condition compared to the control.

### Palantir trajectory inference

ForceAtlas2‐generated matrices served as distances for Palantir (Setty *et al*, 2019) trajectory analysis. For lineage subsetting, the whole AnnData object was reduced to hepatocyte‐, cholangiocyte‐, and DC‐like cells for the hepatic progenitor lineage. The HSC lineage included all HSC‐, SMC‐, and fibroblast‐like cells. Early cells for the hepatic progenitor lineage were automatically defined by searching for the cell with the highest *AFP* expression among all cells expressing *CEBPA*. For the HSC lineage, the cell with the highest *IGFBP2* expression among all cells expressing *DDIT4* was chosen, based on reference data of fetal HSCs (Wesley *et al*, 2022). No terminal or start cells were predefined. Palantir analysis was performed with 30 nearest neighbors, 30 components, and 9,000 numerical waypoints.

### Retrieval of human MASLD signatures in injured HLOs

Previously published 26 and 98 gene signatures predicting fibrosis in MASLD/MASLD (Pantano *et al*, 2021) were transferred to lists, and each cell from the integrated dataset of all orbital‐shaker cultured HLOs (*n* = 8 controls, *n* = 2 OA, *n* = 2 PA, *n* = 2 TGF‐β1, totaling 82,467 cells, quality control and processing as described above) obtained a score for the respective gene lists as described for other inflammatory and fibrosis‐associated scores. Dot plots were generated with scanpy based on scaled count data for the entire dataset or for each cell type individually splitting into the treatment groups. For statistical analysis, lists of numerical score values of all cells from each group (control, OA, PA, TGF‐β1), either in the full dataset or per cell cluster and were forwarded to statistical testing described below.

### Statistics and reproducibility

All statistical tests were performed in python (version 3.6) with scipy.stats (Virtanen *et al*, 2020) (version 1.8.0) and scikit‐posthocs (Terpilowski, 2019) (version 0.6.7). Two groups were compared using the two‐tailed Mann–Whitney *U* test followed by a Bonferroni correction. For three or more groups, the two‐tailed Kruskal–Wallis non‐parametric test followed by a *post hoc* Conover's test with Bonferroni correction was applied. A difference in mean between groups was considered significant at a *P*‐value below 0.05. *N* numbers for individual HLO samples in qPCR and immunohistochemistry experiments indicate individual experiments (individual differentiation cycles starting from day 0 hPSCs). For scRNA‐seq, *n* of samples refer to individual HLO single‐cell suspensions that arose from the same differentiation experiment if performed in replicate, e.g. for day 21 HLOs 3 replicates (*n* = 3) were sequenced, which in this case means from one HLO experiment, single‐cell suspensions from three different wells from the same treatment and time point were harvested and underwent individual library preps.

## Author contributions

**Anja Hess:** Conceptualization; resources; data curation; software; formal analysis; validation; investigation; visualization; methodology; writing – original draft; writing – review and editing. **Stefan D Gentile:** Conceptualization; investigation; methodology; writing – original draft; writing – review and editing. **Amel Ben Saad:** Formal analysis; investigation; visualization; methodology; writing – review and editing. **Raza‐Ur Rahman:** Data curation; methodology; writing – review and editing. **Tim Habboub:** Investigation; methodology. **Daniel S Pratt:** Resources. **Alan C Mullen:** Conceptualization; resources; data curation; supervision; funding acquisition; investigation; writing – original draft; writing – review and editing.

## Disclosure and competing interests statement

ACM receives research funding from Boehringer Ingelheim, Bristol‐Myers Squibb, and GlaxoSmithKlein for other projects and has been a consultant for Third Rock Ventures. R‐UR is co‐founder of deepnostiX GmbH and deepnostiX Private limited.

## Supporting information



Appendix S1

Dataset EV1

Dataset EV2

Dataset EV3

Dataset EV4

Dataset EV5

Dataset EV6

Dataset EV7

Dataset EV8

Dataset EV9

Dataset EV10

Dataset EV11

Source Data for Figure 1

Source Data for Figure 3

Source Data for Figure 5

Source Data for Figure 7

## Data Availability

Raw data for single‐cell RNA sequencing are available at Gene Expression Omnibus (GEO) under the accession GSE207889. Previously published scRNA‐seq data that were re‐analyzed can be found at GSE115469 and E‐MTAB‐8210. Scripts are available under the following links: https://github.com/anjahess/hlo‐scrnaseq, https://github.com/anjahess/organoid‐sirius‐red.
