# Author Correction: Single‐cell transcriptomics stratifies organoid models of metabolic dysfunction‐associated steatotic liver disease

**DOI:** 10.1038/s44318-024-00308-w

**Published:** 2024-11-20

**Authors:** Anja Hess, Stefan D Gentile, Amel Ben Saad, Raza-Ur Rahman, Tim Habboub, Daniel S Pratt, Alan C Mullen

**Affiliations:** 1 Division of Gastroenterology, Massachusetts General Hospital, Harvard Medical School, Boston, MA USA; 2 Klarman Cell Observatory, Broad Institute of MIT and Harvard, Cambridge, MA USA; 3 Autoimmune and Cholestatic Liver Center, Massachusetts General Hospital, Boston, MA USA; 4 Harvard Stem Cell Institute, Cambridge, MA USA; 5 Center for the Study of Inflammatory Bowel Disease, Massachusetts General Hospital, Boston, MA USA; 6 Present Address: Department of Genome Regulation, Max Planck Institute for Molecular Genetics, Berlin, Germany; 7 Present Address: University of Massachusetts Chan Medical School, Worcester, MA USA

## Abstract

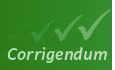

**Correction to:** *The EMBO Journal* (2023) 42:e113898. 10.15252/embj.2023113898 | Published online 14 November 2023

The authors notified the journal after identifying minor labelling errors in Figure 4B. Additionally, they discovered that certain preparation details were inadvertently omitted from the Methods section.

**Figure 4B is corrected**.

**Figure 4:**
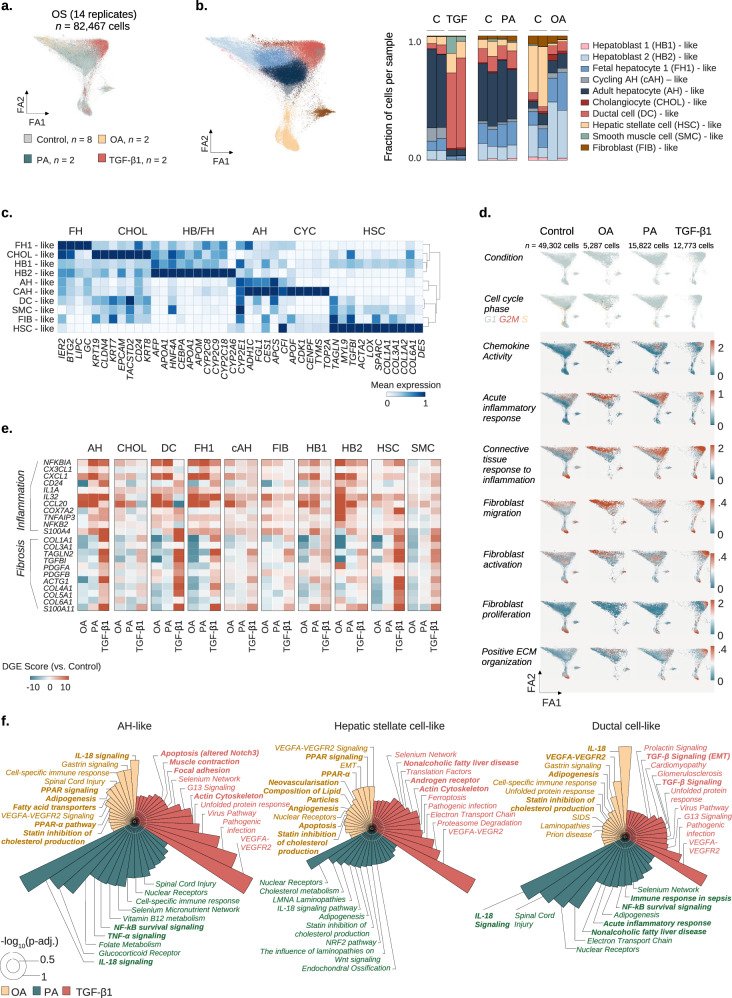
Corrected.

**Figure 4:**
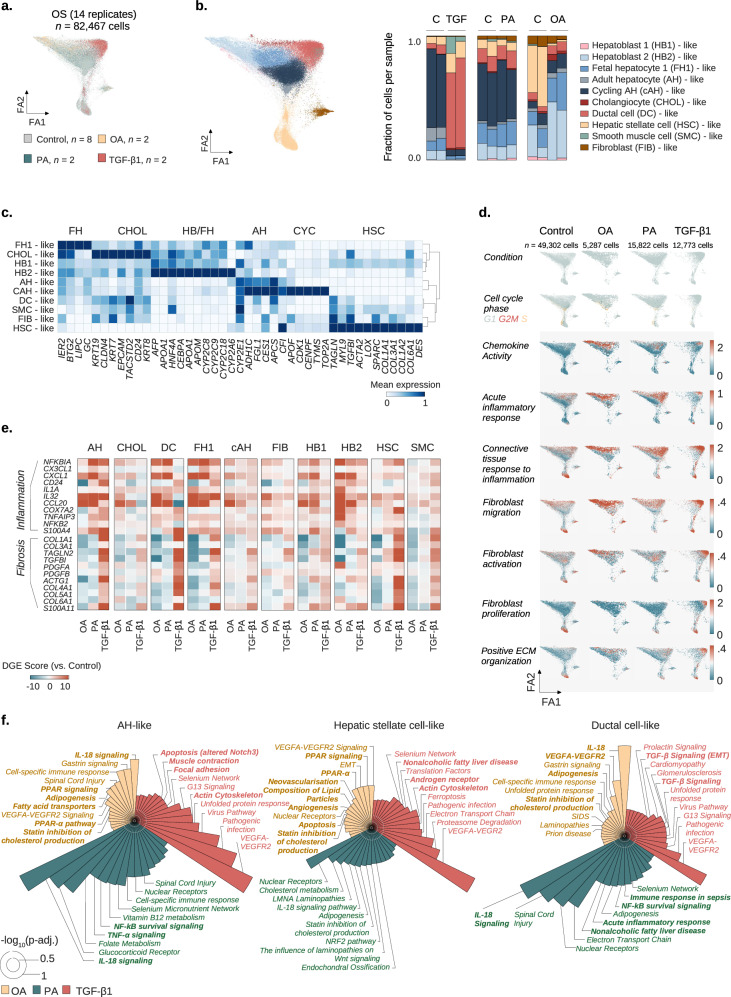
Original.

**The ‘Methods’ section is corrected**.

The Methods section is corrected from:

*Liver injury induction*. Injury media solutions based on Hepatocyte Culture Medium (HCM, Lonza, CC-3198) were prepared to obtain solutions of TGF-β1 (R&D Systems 240-B-002), palmitic acid (PA, Sigma Aldrich P0500), and oleic acid (OA, Sigma Aldrich O1383). HLO media was changed to injury media on day 21. Isolated HLOs were resuspended in the final injury media at the final step of the isolation process (see above) and kept in solution for four days and harvested on day 25. Controls for TGF-β1 were cultured in HCM. PA was dissolved into HCM with 10% BSA and 1% ethanol before dilution to final concentration in HCM. OA was dissolved into DPBS(-/-) with 12.5 mM NaOH and 1.67% BSA at 8 mM before dilution to final concentration in HCM. PA and OA controls were prepared accordingly, omitting the initial step of dissolving the active agent in the carrier solutions.

To: (Changes in bold)

*Liver injury induction*. Injury media solutions based on Hepatocyte Culture Medium (HCM, Lonza, CC-3198) were prepared to obtain solutions of TGF-β1 (R&D Systems 240-B-002), palmitic acid (PA, Sigma Aldrich P0500), and oleic acid (OA, Sigma Aldrich O1383). HLO media was changed to injury media on day 21. Isolated HLOs were resuspended in the final injury media at the final step of the isolation process (see above) and kept in solution for four days and harvested on day 25. Controls for TGF-β1 were cultured in HCM. PA was dissolved **into 100% ethanol at 500 mM and then conjugated** with 10% **BSA in HCM** before dilution to final concentration in HCM. OA **(1.0 M)** was dissolved **in 37.5** **mM NaOH to reach a final concentration of 20** **mM and then conjugated with 5%** BSA **in DPBS** at **5 or** 8 mM before dilution to final concentration in HCM. PA and OA controls were prepared accordingly, omitting the initial step of dissolving the active agent in the carrier solutions.

These changes do not affect the conclusions of the manuscript.

