# Supplementary material for: Single‐cell transcriptomics stratifies organoid models of metabolic dysfunction‐associated steatotic liver disease
Source: EMBO J. 2023 Nov 14;42(24):e113898. doi: 10.15252/embj.2023113898 (PMC10711666; doi:10.15252/embj.2023113898)
Supplement: Supplementary file 1 — Appendix S1 [file EMBJ-42-e113898-s004.pdf]

Appendix

Single cell transcriptomic landscapes of human liver organoids stratify models of metabolic dysfunction-associated steatotic liver disease

Anja Hess[1,#], Stefan D. Gentile[1,3], Amel Ben Saad[1], Raza-Ur Rahman[1], Tim Habboub[1], Daniel S. Pratt[1,5] and Alan C. Mullen[1,2,3,4*]

1. Division of Gastroenterology, Massachusetts General Hospital, Harvard Medical School, Boston, MA, USA
2. Harvard Stem Cell Institute, Cambridge, MA, USA
3. Klarman Cell Observatory, Broad Institute of MIT and Harvard, Cambridge, MA, USA
4. Center for the Study of Inflammatory Bowel Disease, Massachusetts General Hospital, Boston, MA, USA
5. Autoimmune and Cholestatic Liver Center, Massachusetts General Hospital, Boston, MA, USA

# Current Address: Max Planck Institute for Molecular Genetics, Department of Genome Regulation, Berlin, Germany

* Corresponding author. Current address: 304 Lazare Research Building, University of Massachusetts Chan Medical School, Worcester, MA 01605, USA. (508) 856-4005. Email: alan.mullen@umassmed.edu

Abbreviations: HLOs, Human liver organoids; OS, Orbital shaker; ULA, Ultra-low attachment plate; D21, Day 21 of differentiation; D25, Day 25 of differentiation; OA, Oleic acid at 500 µM; PA, Palmitic acid at 500 µM; TGF-β1, TGF-β1 at 10 ng/µl

Table of contents:

Appendix Fig. S1 Representative images of fixed HLOs. Corresponding to Figure 1c……..……….… p. 2

Appendix Fig. S2 Replicate distribution, marker gene expression, annotation for 10X scRNA-seq data of day 21 ULA-HLOs. Corresponding to Fig. 1d…………………………………… p. 4

Appendix Fig. S3 Induction of fibrotic phenotypes (morphology, qPCR, Sirius red staining) in HLOs treated with OA, PA, and TGF-β1. Corresponding to Fig. 3……………………………. p. 6

Appendix Fig. S4 Marker gene expression and replicate distribution in cells from control and injured HLOs. Corresponding to Fig. 4b…………………………………………………………… p. 8

Appendix Fig. S5 Pathway enrichment analysis, cell-type resolved. Corresponding to Fig. 4f……….. p. 11

Appendix Fig. S6 Differential cell-cell interactions in OS-HLOs upon OA, PA, and TGF-β1 treatment. Corresponding to Fig. 5……………………………………………………………………….. p. 14

Appendix Fig. S7 Comparison of normalized expression and imputed expression for two major terminal states in OS-HLOs. Corresponding to Fig. 6g……………………………………. p. 18

Appendix Fig. S8 Cell type-resolved expression of genes predicting fibrosis stages. Corresponding to Fig. 7c……………………………………………………………………………………… p. 20

References ……………………………………………………………………………………………. p. 22

Fig. S1

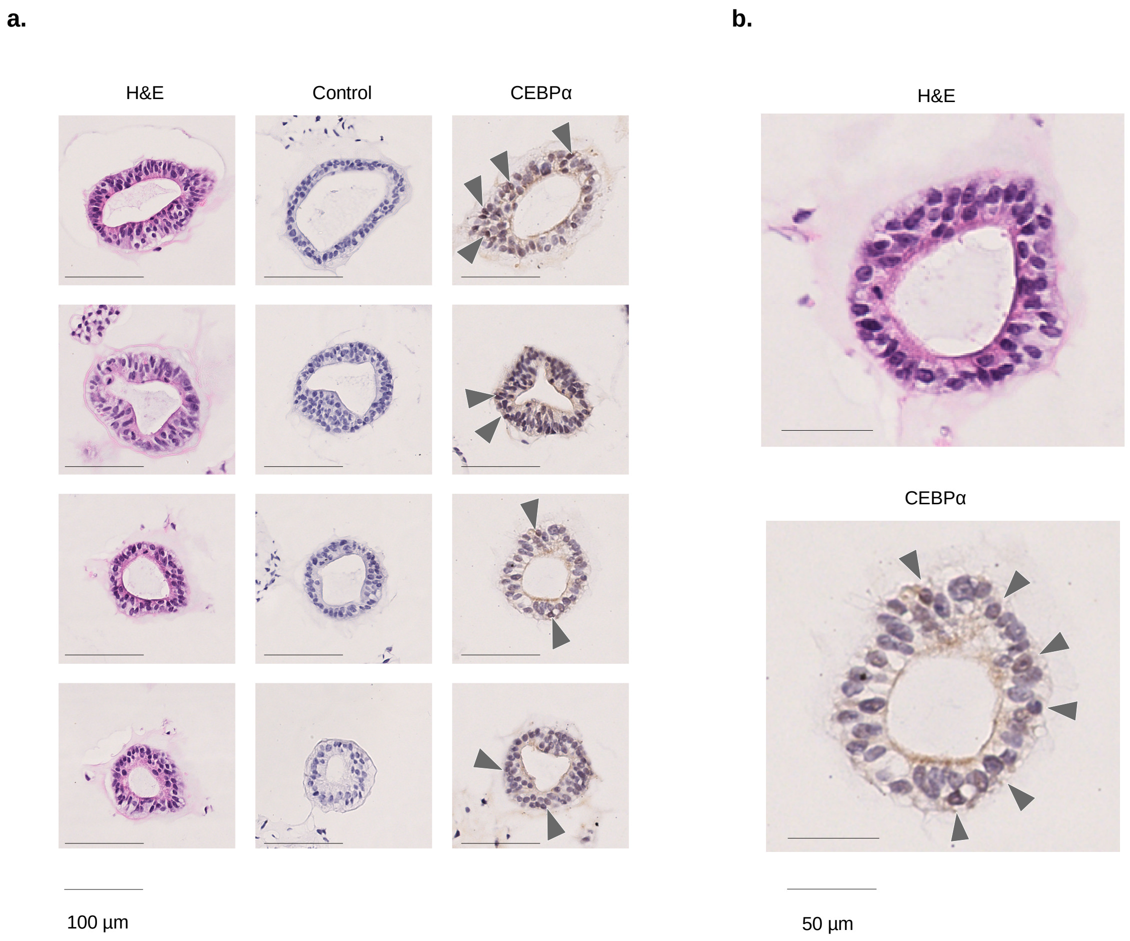

Appendix Figure S1. Representative images of fixed HLOs. Corresponding to Fig. 1c.

a. Panels showing individual HLOs from left to right: H&E, secondary antibody only (Control), anti-CEBPα (nuclear staining indicated by arrowheads). Scale bars 100 µm.

b. Two HLOs from a., increased magnification for representative indication of nuclear CEBPα staining (arrowheads). Scale bars 50 µm.

Fig. S2

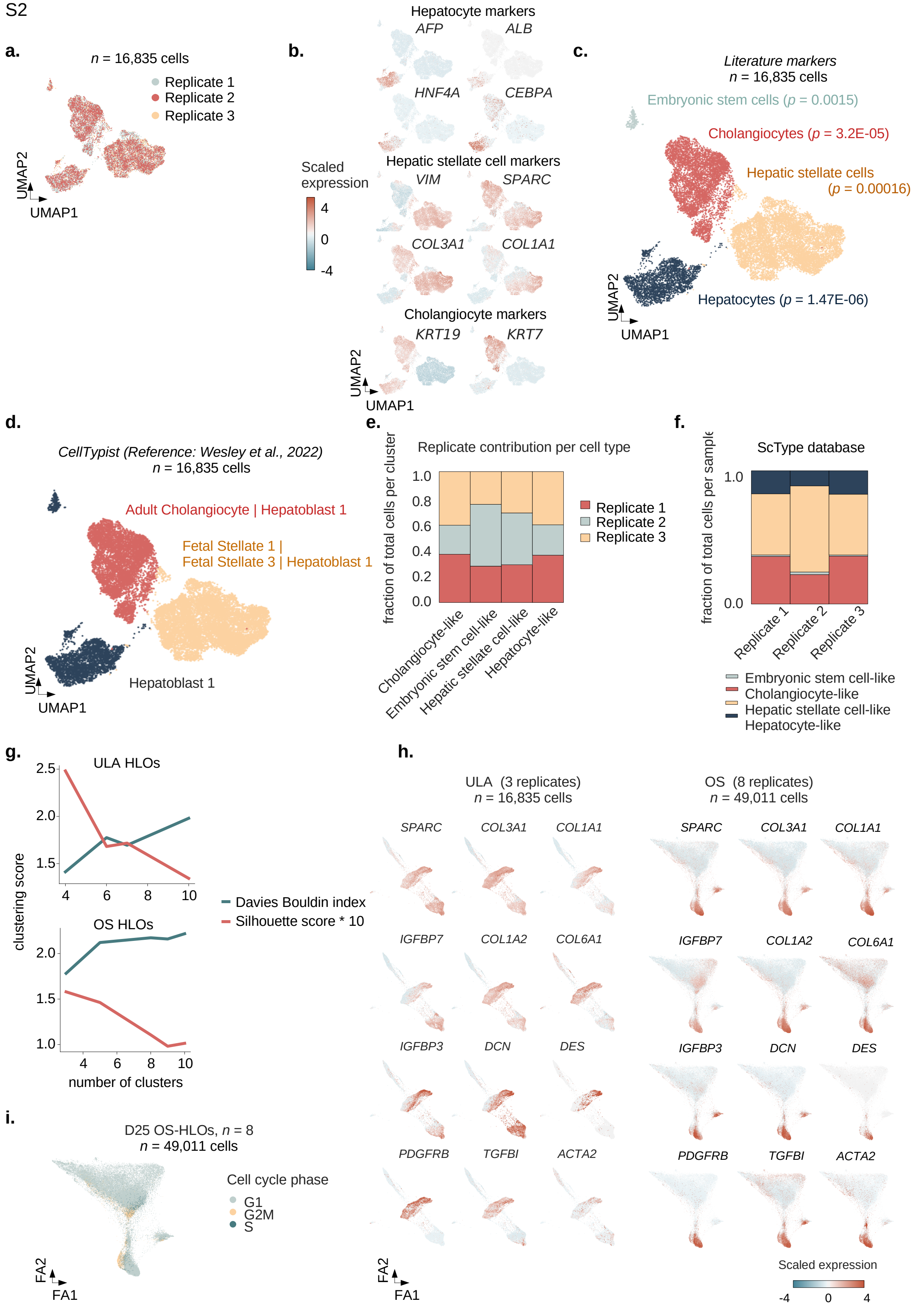
a.
n = 16,835 cells
Replicate 1
Replicate 2
Replicate 3
UMAP2
UMAP1
b.
Hepatocyte markers
AFP
ALB
HNF4A
CEBPA
Hepatic stellate cell markers
VIM
SPARC
COL3A1
COL1A1
Cholangiocyte markers
KRT19
KRT7
Scaled expression
4
0
-4
UMAP2
UMAP1
c.
Literature markers
n = 16,835 cells
Embryonic stem cells (p = 0.0015)
Cholangiocytes (p = 3.2E-05)
Hepatic stellate cells (p = 0.00016)
Hepatocytes (p = 1.47E-06)
UMAP2
UMAP1
d.
CellTypist (Reference: Wesley et al., 2022)
n = 16,835 cells
Adult Cholangiocyte | Hepatoblast 1
Fetal Stellate 1 |
Fetal Stellate 3 | Hepatoblast 1
Hepatoblast 1
UMAP2
UMAP1
e.
Replicate contribution per cell type
fraction of total cells per cluster
1.0
0.8
0.6
0.4
0.2
0.0
Cholangiocyte-like
Embryonic stem cell-like
Hepatic stellate cell-like
Hepatocyte-like
Replicate 1
Replicate 2
Replicate 3
f.
ScType database
fraction of total cells per sample
1.0
0.0
Replicate 1
Replicate 2
Replicate 3
Embryonic stem cell-like
Cholangiocyte-like
Hepatic stellate cell-like
Hepatocyte-like
g.
ULA HLOs
2.5
2.0
1.5
4
6
8
10
OS HLOs
2.0
1.5
1.0
4
6
8
10
clustering score
number of clusters
Davies Bouldin index
Silhouette score * 10
h.
ULA (3 replicates)
n = 16,835 cells
OS (8 replicates)
n = 49,011 cells
SPARC
COL3A1
COL1A1
IGFBP7
COL1A2
COL6A1
IGFBP3
DCN
DES
PDGFRB
TGFBI
ACTA2
FA2
FA1
Scaled expression
-4
0
4
i.
D25 OS-HLOs, n = 8
n = 49,011 cells
Cell cycle phase
G1
G2M
S
FA2
FA1

Appendix Figure S2. Replicate distribution, marker gene expression, and additional annotation for 10X scRNA-seq data of day 21 ULA-HLOs. Corresponding to Fig. 1d and 2d.

a. UMAP plot mapping single cells from day 21 ULA-HLOs, colored by replicate.

b. UMAP plot from a., showing the scaled expression of liver cell type marker genes.

c. UMAP plot from a., showing the cell type annotations generated by literature-based annotation (Methods).

d. UMAP plot from a., showing the cell type annotations generated by the CellTypist[1] classifier. CellTypist was trained on cells from human liver scRNA-seq data[2]. Most probable matches for each cell type are displayed in the legend, if more than one cell type matches the query sample, alternate labels are separated by the "|" symbol.

e. Barplot indicating the replicate distribution as the proportion of total cells per cluster calculated with the Leiden algorithm at resolution 0.1 and ScType[3] database.

f. Barplots showing cell type distributions across replicates as the proportion of each cell type per total cells per sample. Annotation performed with ScType[3] database followed by statistical enrichment with GSEA-py enrichr[4].

g. Lineplots showing cluster robustness metrics (Davies Bouldin index and Silhoutte score) in dependence to number of clusters generated through increasing Leiden resolutions (0.1 – 1) for OS- and ULA-HLO scRNAseq data. Leiden resolution 0.1 generating four clusters in each context was chosen for subsequent analysis. Silhouette score multiplied by 10 for joint visualization.

h. ForceAtlas2 representations of the cells from OS- and ULA-HLOs, colored by the mean scaled expression of HSC marker genes, corresponding to Fig. 2d.

i. ForceAtlas2 plot mapping cells from OS-HLOs (n = 8) colored by cell cycle phase.

Fig. S3

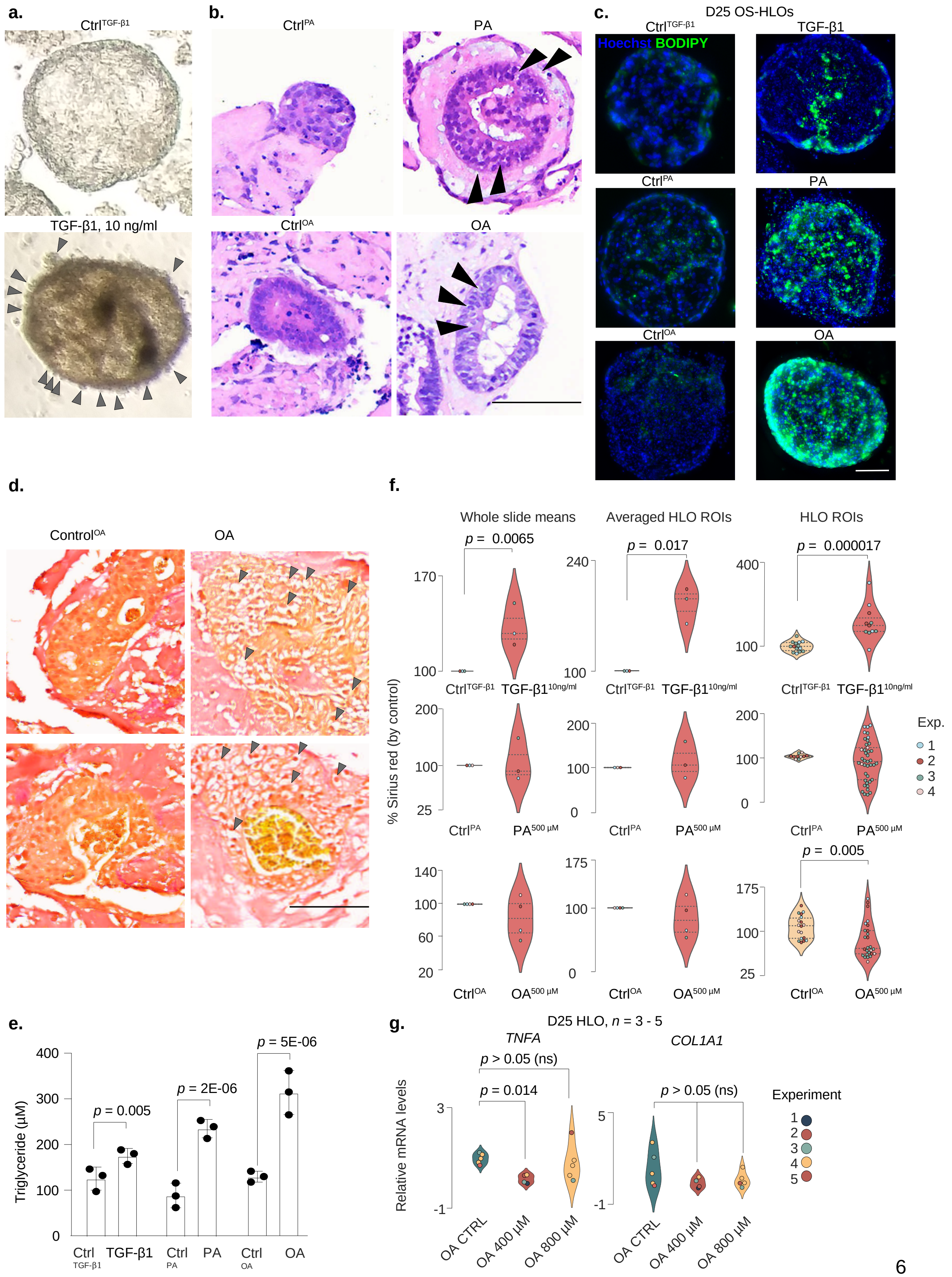

a.
Ctrl TGF-β1
TGF-β1, 10 ng/ml
b.
Ctrl PA
PA
Ctrl OA
OA
c.
D25 OS-HLOs
Ctrl TGF-β1
TGF-β1
Hoechst BODIPY
Ctrl PA
PA
Ctrl OA
OA
d.
Control OA
OA
f.
Whole slide means
Averaged HLO ROIs
HLO ROIs
p = 0.0065
p = 0.017
p = 0.000017
% Sirius red (by control)
Ctrl TGF-β1
TGF-β1 10ng/ml
Ctrl PA
PA 500 μM
Ctrl OA
OA 500 μM
p = 0.005
Exp.
1
2
3
4
e.
Triglyceride (μM)
p = 0.005
p = 2E-06
p = 5E-06
Ctrl TGF-β1
TGF-β1
Ctrl PA
PA
Ctrl OA
OA
g.
D25 HLO, n = 3 - 5
TNFA
COL1A1
p > 0.05 (ns)
p = 0.014
Relative mRNA levels
OA CTRL
OA 400 μM
OA 800 μM
Experiment
1
2
3
4
5

Appendix Figure S3. Induction of fibrotic phenotypes in OS-cultured HLOs. Corresponding to Fig. 3.

a. Enlarged representative brightfield images from Fig. 3, showing day 25 OS-HLOs (control, top) and treated with TGF-β1 (10 ng/ml, bottom). Arrowheads indicate areas of surface roughening.

b. OS-HLOs acquire intracellular vacuoles consistent with steatosis after the exposure to FFAs. H&E stainings of day 25 OS-HLOs treated with PA or OA for four days. Arrowheads indicate vacuoles. Scale bar, 100 µm.

c. Representative BODIPY immunofluorescence images of day 25 OS-HLOs derived from an additional PSC line (iPSC4) treated TGF-β1, PA, and OA (right) or the control conditions(left). BODIPY staining (green), nuclear staining with Hoechst (blue). Scale bar, 50 µm. Images are representative of two experiments performed with HLOs differentiated from iPSC4 cells.

d. Sirius red stainings of day 25 OS-HLOs treated with the OA-Control (left) and OA (right), showing collagen staining reduction and vacuole deposition in OA-treated HLOs. Arrowheads indicate vacuoles. Scale bar 100 µm. *N* = 3 experiments.

e. Triglyceride (TAG) content is increased in OS-HLOs treated with TGF-β1, PA, and OA for four days derived from iPSC4 cells. Bar plots display the total TAG concentration in µM for a representative experiment where each dot represents one well of HLOs. Kruskal-Wallis test (two-tailed) followed by a post hoc Conover's test. *P*-values as indicated in the figure. Data are shown for one experiment and are representative of four experiments performed on HLOs differentiated from iPSC4 cells.

f. Sirius red quantification in HLOs treated with TGF-β1, PA, and OA. Shown is the percentage of Sirius red positive tissue by total tissue (normalized by the mean of the respective control HLOs in each experiment). Quantification based on the whole slide Sirius red percentage mean (left), mean of averaged 200 x 200 µm regions of interest (ROIs) around single HLOs (center), or Sirius red percentage per tissue for each HLO ROI (right). Kruskal-Wallis test (two-tailed) followed by a post hoc Conover's test with Bonferroni correction. *N* ≥ 4 HLOs from one individual experiment (organoid ROIs), *n* = 3-4 individual experiments (all other plots).

g. qRT-PCR results for *COL1A1* and *TNFA* in day 25 OS-HLOs treated with 400 and 800 µM OA. Shown are relative mRNA levels normalized to *GAPDH* (2^{-ddCt}). Kruskal Wallis test (two-tailed) followed by Conover's post hoc test with Bonferroni correction. Ns, not significant. *N* = 3-4 individual experiments.

Fig. S4

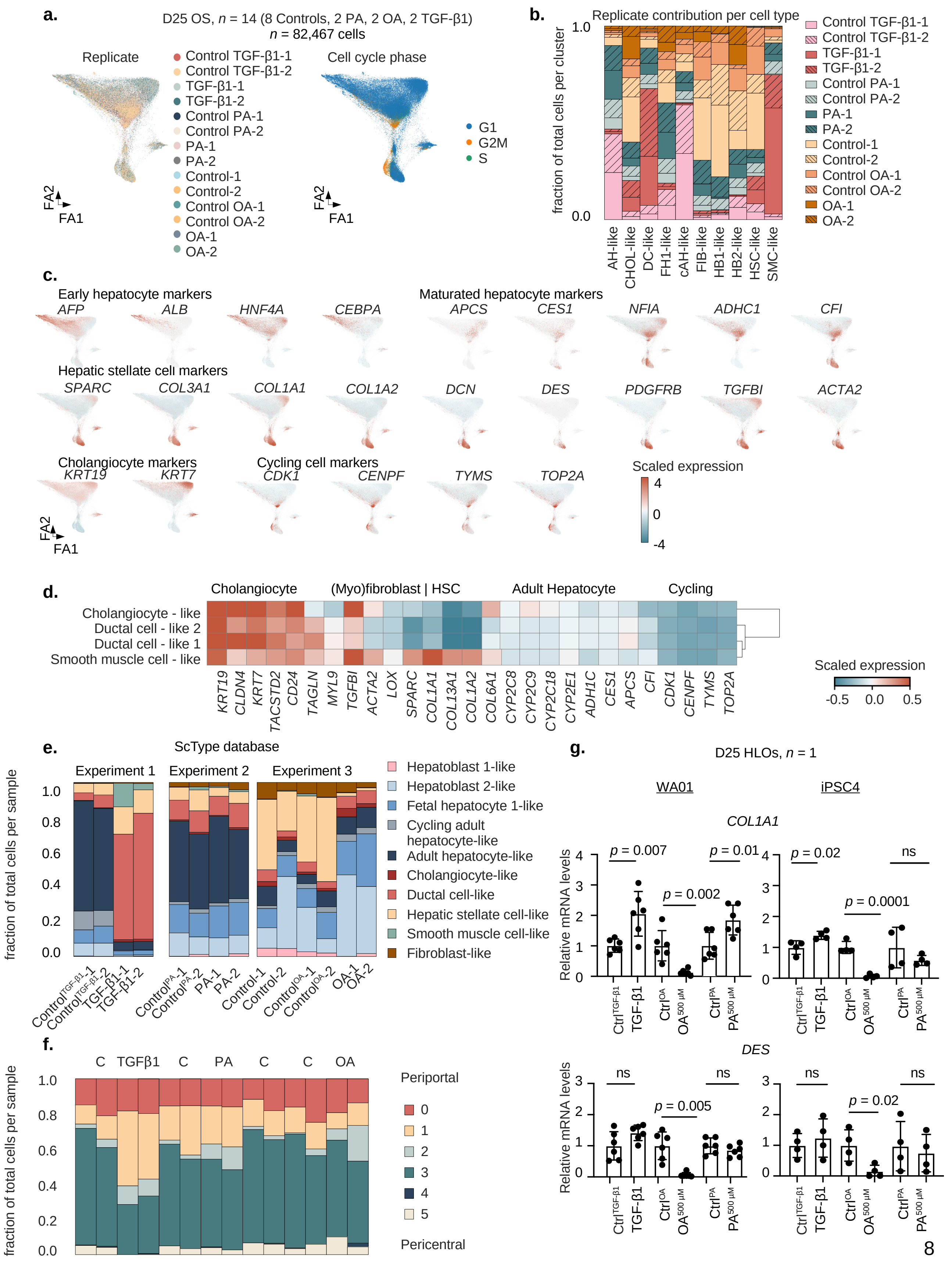

a.
D25 OS, n = 14 (8 Controls, 2 PA, 2 OA, 2 TGF-β1)
n = 82,467 cells
Replicate
Cell cycle phase
Control TGF-β1-1
Control TGF-β1-2
TGF-β1-1
TGF-β1-2
Control PA-1
Control PA-2
PA-1
PA-2
Control-1
Control-2
Control OA-1
Control OA-2
OA-1
OA-2
G1
G2M
S
FA2
FA1
b.
Replicate contribution per cell type
fraction of total cells per cluster
AH-like
CHOL-like
DC-like
FH1-like
cAH-like
FIB-like
HB1-like
HB2-like
HSC-like
SMC-like
c.
Early hepatocyte markers
AFP
ALB
HNF4A
CEBPA
Matured hepatocyte markers
APCS
CES1
NFIA
ADHC1
CFI
Hepatic stellate cell markers
SPARC
COL3A1
COL1A1
COL1A2
DCN
DES
PDGFRB
TGFBI
ACTA2
Cholangiocyte markers
KRT19
KRT7
Cycling cell markers
CDK1
CENPF
TYMS
TOP2A
Scaled expression
d.
Cholangiocyte
(Myo)fibroblast | HSC
Adult Hepatocyte
Cycling
Cholangiocyte - like
Ductal cell - like 2
Ductal cell - like 1
Smooth muscle cell - like
KRT19
CLDN4
KRT7
TACSTD2
CD24
TAGLN
MYL9
TGFBI
ACTA2
LOX
SPARC
COL1A1
COL13A1
COL1A2
COL6A1
CYP2C8
CYP2C9
CYP2C18
CYP2E1
ADH1C
CES1
APCS
CFI
CDK1
CENPF
TYMS
TOP2A
Scaled expression
e.
ScType database
Experiment 1
Experiment 2
Experiment 3
fraction of total cells per sample
Hepatoblast 1-like
Hepatoblast 2-like
Fetal hepatocyte 1-like
Cycling adult hepatocyte-like
Adult hepatocyte-like
Cholangiocyte-like
Ductal cell-like
Hepatic stellate cell-like
Smooth muscle cell-like
Fibroblast-like
f.
C TGFβ1 C PA C C OA
Periportal
Pericentral
g.
D25 HLOs, n = 1
WA01
iPSC4
COL1A1
DES
Relative mRNA levels
p = 0.007
p = 0.01
p = 0.002
p = 0.02
p = 0.0001
p = 0.005
ns

h.

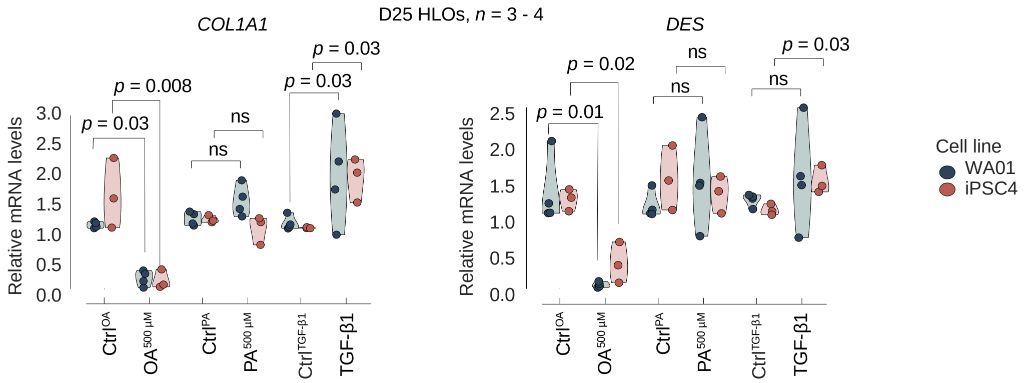

Appendix Figure S4. Marker gene expression and replicate distribution in cells from control and injured HLOs. Corresponding to Fig. 4.

a. ForceAtlas2 plots mapping cells from OS-HLOs treated with OA (500 µM, *n* = 2), PA (500 µM, *n* = 2), TGF-β1 (10 ng/ml, *n* = 2), and their respective controls (*n* = 8) colored by replicate (left) and cell cycle phase (right).

b. Barplot indicating the replicate distribution as the proportion of total cells per cluster for each cluster. Cell clusters are colored in the order of their appearance. AH - adult hepatocyte-like, CHOL – cholangiocyte-like, DCs – ductal cell-like, FH1 – fetal hepatocyte 1-like, cAH – cycling adult hepatocyte-like, FIB – fibroblast-like, HB1 – hepatoblast 1-like, HB2 – hepatoblast 2-like, HSCs – hepatic stellate cell-like, SMCs – smooth muscle cell-like.

c. ForceAtlas2 plots from a., showing the scaled expression of canonical marker genes.

d. Matrixplot shows the scaled mean expression for marker genes in cholangiocyte sub-clusters. Canonical marker genes (bottom) are sorted by cell type (top). Hierarchical clustering is represented by the dendrogram on the right.

e. Barplots showing cell type distributions across replicates as the proportion of each cell type per total cells per sample. Cell clusters are colored in the order of their appearance.

f. Barplots showing zonal distributions (Methods) across replicates as the proportion of each cell type per total cells per sample for adult-hepatocyte like cells. Zonation categories are colored in the order of their appearance.

g. Representative qRT-PCR results for *COL1A1* and *DES* in two PSC lines. Shown are relative mRNA levels normalized to *ACTB* (2^{-ddCt}). T-test (two-tailed), *p*-values as indicated in the figure, ns, not significant. Data are shown for one experiment where each dot represents one well of HLOs. Data are representative for 3 - 4 experiments performed on HLOs differentiated from each PSC line.

h. Summary of all qRT-PCR experiments for *COL1A1* and *DES* in two PSC lines. Shown are relative mRNA levels normalized to *ACTB* (2^{-ddCt}). Kruskal Wallis test (two-tailed) followed by Conover's post hoc test. $P < 0.05$ (*), $p < 0.01$ (**), $p < 0.001$ (***), ns, not significant. $N = 3 - 4$ experiments.

Fig. S5

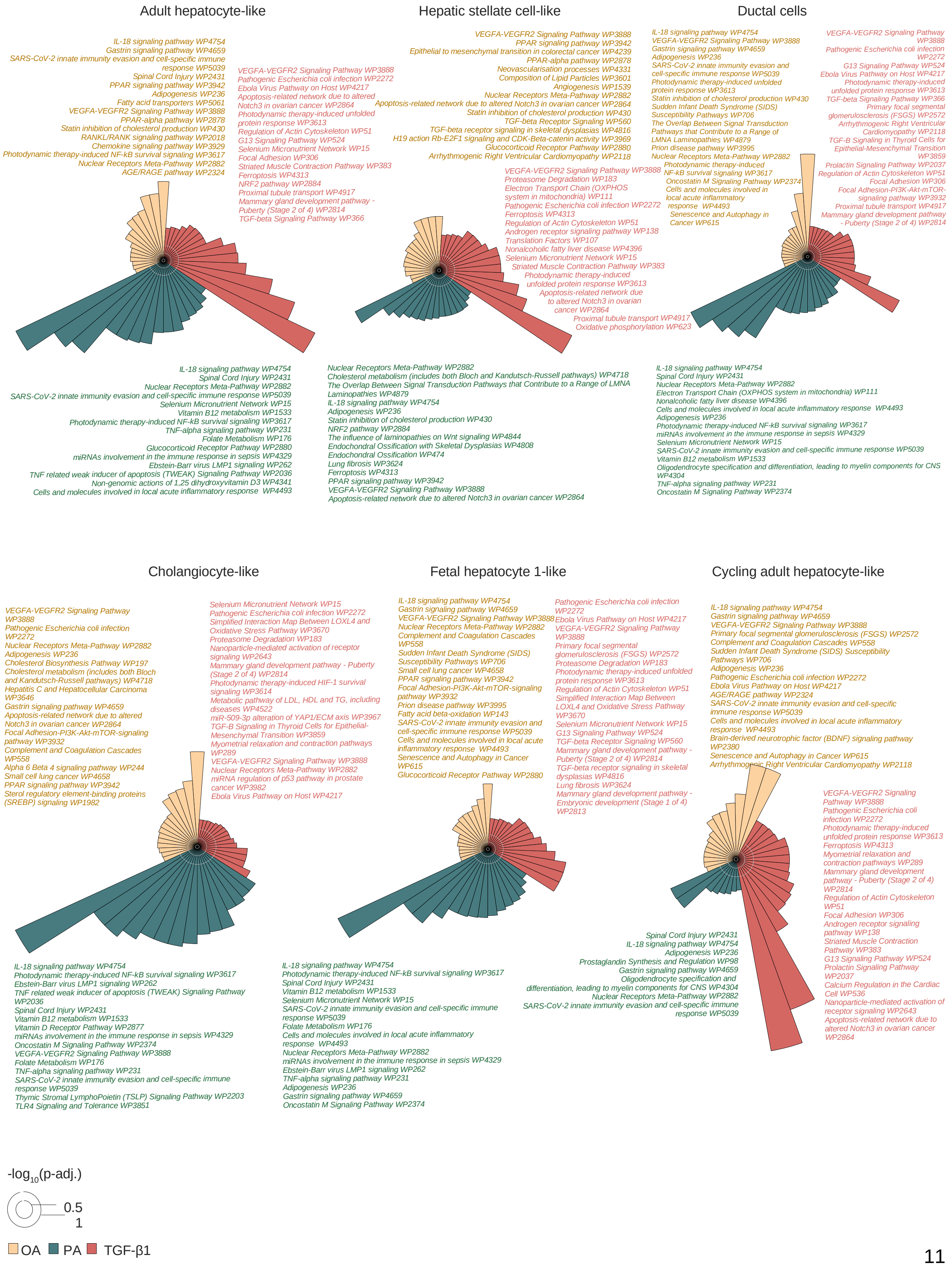
Adult hepatocyte-like
IL-18 signaling pathway WP4754
Gastrin signaling pathway WP4659
SARS-CoV-2 innate immunity evasion and cell-specific immune response WP5039
Spinal Cord Injury WP2431
PPAR signaling pathway WP3942
Adipogenesis WP236
Fatty acid transporters WP5061
VEGFA-VEGFR2 Signaling Pathway WP3888
PPAR-alpha pathway WP2878
Statin inhibition of cholesterol production WP430
RANKL/RANK signaling pathway WP2018
Chemokine signaling pathway WP3929
Photodynamic therapy-induced NF-kB survival signaling WP3617
Nuclear Receptors Meta-Pathway WP2882
AGE/RAGE pathway WP2324
VEGFA-VEGFR2 Signaling Pathway WP3888
Pathogenic Escherichia coli infection WP2272
Ebola Virus Pathway on Host WP4217
Apoptosis-related network due to altered Notch3 in ovarian cancer WP2864
Photodynamic therapy-induced unfolded protein response WP3613
Regulation of Actin Cytoskeleton WP51
G13 Signaling Pathway WP524
Selenium Micronutrient Network WP15
Focal Adhesion WP306
Striated Muscle Contraction Pathway WP383
Ferroptosis WP4313
NRF2 pathway WP2884
Proximal tubule transport WP4917
Mammary gland development pathway - Puberty (Stage 2 of 4) WP2814
TGF-beta Signaling Pathway WP366
IL-18 signaling pathway WP4754
Spinal Cord Injury WP2431
Nuclear Receptors Meta-Pathway WP2882
SARS-CoV-2 innate immunity evasion and cell-specific immune response WP5039
Selenium Micronutrient Network WP15
Vitamin B12 metabolism WP1533
Photodynamic therapy-induced NF-kB survival signaling WP3617
TNF-alpha signaling pathway WP231
Folate Metabolism WP176
Glucocorticoid Receptor Pathway WP2880
miRNAs involvement in the immune response in sepsis WP4329
Ebstein-Barr virus LMP1 signaling WP262
TNF related weak inducer of apoptosis (TWEAK) Signaling Pathway WP2036
Non-genomic actions of 1,25 dihydroxyvitamin D3 WP4341
Cells and molecules involved in local acute inflammatory response WP4493
Hepatic stellate cell-like
VEGFA-VEGFR2 Signaling Pathway WP3888
PPAR signaling pathway WP3942
Epithelial to mesenchymal transition in colorectal cancer WP4239
PPAR-alpha pathway WP2878
Neovascularisation processes WP4331
Composition of Lipid Particles WP3601
Angiogenesis WP1539
Nuclear Receptors Meta-Pathway WP2882
Apoptosis-related network due to altered Notch3 in ovarian cancer WP2864
Statin inhibition of cholesterol production WP430
TGF-beta Receptor Signaling WP560
TGF-beta receptor signaling in skeletal dysplasias WP4816
H19 action Rb-E2F1 signaling and CDK-Beta-catenin activity WP3969
Glucocorticoid Receptor Pathway WP2880
Arrhythmogenic Right Ventricular Cardiomyopathy WP2118
VEGFA-VEGFR2 Signaling Pathway WP3888
Proteasome Degradation WP183
Electron Transport Chain (OXPHOS system in mitochondria) WP111
Pathogenic Escherichia coli infection WP2272
Ferroptosis WP4313
Regulation of Actin Cytoskeleton WP51
Androgen receptor signaling pathway WP138
Translation Factors WP107
Nonalcoholic fatty liver disease WP4396
Selenium Micronutrient Network WP15
Striated Muscle Contraction Pathway WP383
Photodynamic therapy-induced unfolded protein response WP3613
Apoptosis-related network due to altered Notch3 in ovarian cancer WP2864
Proximal tubule transport WP4917
Oxidative phosphorylation WP623
Nuclear Receptors Meta-Pathway WP2882
Cholesterol metabolism (includes both Bloch and Kandutsch-Russell pathways) WP4718
The Overlap Between Signal Transduction Pathways that Contribute to a Range of LMNA Laminopathies WP4879
IL-18 signaling pathway WP4754
Adipogenesis WP236
Statin inhibition of cholesterol production WP430
NRF2 pathway WP2884
The influence of laminopathies on Wnt signaling WP4844
Endochondral Ossification with Skeletal Dysplasias WP4808
Endochondral Ossification WP474
Lung fibrosis WP3624
Ferroptosis WP4313
PPAR signaling pathway WP3942
VEGFA-VEGFR2 Signaling Pathway WP3888
Apoptosis-related network due to altered Notch3 in ovarian cancer WP2864
Ductal cells
IL-18 signaling pathway WP4754
VEGFA-VEGFR2 Signaling Pathway WP3888
Gastrin signaling pathway WP4659
Adipogenesis WP236
SARS-CoV-2 innate immunity evasion and cell-specific immune response WP5039
Photodynamic therapy-induced unfolded protein response WP3613
Statin inhibition of cholesterol production WP430
Sudden Infant Death Syndrome (SIDS) Susceptibility Pathways WP706
The Overlap Between Signal Transduction Pathways that Contribute to a Range of LMNA Laminopathies WP4879
Prion disease pathway WP3995
Nuclear Receptors Meta-Pathway WP2882
Photodynamic therapy-induced NF-kB survival signaling WP3617
Oncostatin M Signaling Pathway WP2374
Cells and molecules involved in local acute inflammatory response WP4493
Senescence and Autophagy in Cancer WP615
VEGFA-VEGFR2 Signaling Pathway WP3888
Pathogenic Escherichia coli infection WP2272
G13 Signaling Pathway WP524
Ebola Virus Pathway on Host WP4217
Photodynamic therapy-induced unfolded protein response WP3613
TGF-beta Signaling Pathway WP366
Primary focal segmental glomerulosclerosis (FSGS) WP2572
Arrhythmogenic Right Ventricular Cardiomyopathy WP2118
TGF-B Signaling in Thyroid Cells for Epithelial-Mesenchymal Transition WP3859
Prolactin Signaling Pathway WP2037
Regulation of Actin Cytoskeleton WP51
Focal Adhesion WP306
Focal Adhesion-PI3K-Akt-mTOR-signaling pathway WP3932
Proximal tubule transport WP4917
Mammary gland development pathway - Puberty (Stage 2 of 4) WP2814
IL-18 signaling pathway WP4754
Spinal Cord Injury WP2431
Nuclear Receptors Meta-Pathway WP2882
Electron Transport Chain (OXPHOS system in mitochondria) WP111
Nonalcoholic fatty liver disease WP4396
Cells and molecules involved in local acute inflammatory response WP4493
Adipogenesis WP236
Photodynamic therapy-induced NF-kB survival signaling WP3617
miRNAs involvement in the immune response in sepsis WP4329
Selenium Micronutrient Network WP15
SARS-CoV-2 innate immunity evasion and cell-specific immune response WP5039
Vitamin B12 metabolism WP1533
Oligodendrocyte specification and differentiation, leading to myelin components for CNS WP4304
TNF-alpha signaling pathway WP231
Oncostatin M Signaling Pathway WP2374
Cholangiocyte-like
VEGFA-VEGFR2 Signaling Pathway WP3888
Pathogenic Escherichia coli infection WP2272
Nuclear Receptors Meta-Pathway WP2882
Adipogenesis WP236
Cholesterol Biosynthesis Pathway WP197
Cholesterol metabolism (includes both Bloch and Kandutsch-Russell pathways) WP4718
Hepatitis C and Hepatocellular Carcinoma WP3646
Gastrin signaling pathway WP4659
Apoptosis-related network due to altered Notch3 in ovarian cancer WP2864
Focal Adhesion-PI3K-Akt-mTOR-signaling pathway WP3932
Complement and Coagulation Cascades WP558
Alpha 6 Beta 4 signaling pathway WP244
Small cell lung cancer WP4658
PPAR signaling pathway WP3942
Sterol regulatory element-binding proteins (SREBP) signaling WP1982
Selenium Micronutrient Network WP15
Pathogenic Escherichia coli infection WP2272
Simplified Interaction Map Between LOXL4 and Oxidative Stress Pathway WP3670
Proteasome Degradation WP183
Nanoparticle-mediated activation of receptor signaling WP2643
Mammary gland development pathway - Puberty (Stage 2 of 4) WP2814
Photodynamic therapy-induced HIF-1 survival signaling WP3614
Metabolic pathway of LDL, HDL and TG, including diseases WP4522
miR-509-3p alteration of YAP1/ECM axis WP3967
TGF-B Signaling in Thyroid Cells for Epithelial-Mesenchymal Transition WP3859
Myometrial relaxation and contraction pathways WP289
VEGFA-VEGFR2 Signaling Pathway WP3888
Nuclear Receptors Meta-Pathway WP2882
miRNA regulation of p53 pathway in prostate cancer WP3982
Ebola Virus Pathway on Host WP4217
IL-18 signaling pathway WP4754
Photodynamic therapy-induced NF-kB survival signaling WP3617
Ebstein-Barr virus LMP1 signaling WP262
TNF related weak inducer of apoptosis (TWEAK) Signaling Pathway WP2036
Spinal Cord Injury WP2431
Vitamin B12 metabolism WP1533
Vitamin D Receptor Pathway WP2877
miRNAs involvement in the immune response in sepsis WP4329
Oncostatin M Signaling Pathway WP2374
VEGFA-VEGFR2 Signaling Pathway WP3888
Folate Metabolism WP176
TNF-alpha signaling pathway WP231
SARS-CoV-2 innate immunity evasion and cell-specific immune response WP5039
Thymic Stromal LymphoPoietin (TSLP) Signaling Pathway WP2203
TLR4 Signaling and Tolerance WP3851
Fetal hepatocyte 1-like
IL-18 signaling pathway WP4754
Gastrin signaling pathway WP4659
VEGFA-VEGFR2 Signaling Pathway WP3888
Nuclear Receptors Meta-Pathway WP2882
Complement and Coagulation Cascades WP558
Sudden Infant Death Syndrome (SIDS) Susceptibility Pathways WP706
Small cell lung cancer WP4658
PPAR signaling pathway WP3942
Focal Adhesion-PI3K-Akt-mTOR-signaling pathway WP3932
Prion disease pathway WP3995
Fatty acid beta-oxidation WP143
SARS-CoV-2 innate immunity evasion and cell-specific immune response WP5039
Cells and molecules involved in local acute inflammatory response WP4493
Senescence and Autophagy in Cancer WP615
Glucocorticoid Receptor Pathway WP2880
Pathogenic Escherichia coli infection WP2272
Ebola Virus Pathway on Host WP4217
VEGFA-VEGFR2 Signaling Pathway WP3888
Primary focal segmental glomerulosclerosis (FSGS) WP2572
Proteasome Degradation WP183
Photodynamic therapy-induced unfolded protein response WP3613
Regulation of Actin Cytoskeleton WP51
Simplified Interaction Map Between LOXL4 and Oxidative Stress Pathway WP3670
Selenium Micronutrient Network WP15
G13 Signaling Pathway WP524
TGF-beta Receptor Signaling WP560
Mammary gland development pathway - Puberty (Stage 2 of 4) WP2814
TGF-beta receptor signaling in skeletal dysplasias WP4816
Lung fibrosis WP3624
Mammary gland development pathway - Embryonic development (Stage 1 of 4) WP2813
IL-18 signaling pathway WP4754
Photodynamic therapy-induced NF-kB survival signaling WP3617
Spinal Cord Injury WP2431
Vitamin B12 metabolism WP1533
Selenium Micronutrient Network WP15
SARS-CoV-2 innate immunity evasion and cell-specific immune response WP5039
Folate Metabolism WP176
Cells and molecules involved in local acute inflammatory response WP4493
Nuclear Receptors Meta-Pathway WP2882
miRNAs involvement in the immune response in sepsis WP4329
Ebstein-Barr virus LMP1 signaling WP262
TNF-alpha signaling pathway WP231
Adipogenesis WP236
Gastrin signaling pathway WP4659
Oncostatin M Signaling Pathway WP2374
Cycling adult hepatocyte-like
IL-18 signaling pathway WP4754
Gastrin signaling pathway WP4659
VEGFA-VEGFR2 Signaling Pathway WP3888
Primary focal segmental glomerulosclerosis (FSGS) WP2572
Complement and Coagulation Cascades WP558
Sudden Infant Death Syndrome (SIDS) Susceptibility Pathways WP706
Adipogenesis WP236
Pathogenic Escherichia coli infection WP2272
Ebola Virus Pathway on Host WP4217
AGE/RAGE pathway WP2324
SARS-CoV-2 innate immunity evasion and cell-specific immune response WP5039
Cells and molecules involved in local acute inflammatory response WP4493
Brain-derived neurotrophic factor (BDNF) signaling pathway WP2380
Senescence and Autophagy in Cancer WP615
Arrhythmogenic Right Ventricular Cardiomyopathy WP2118
VEGFA-VEGFR2 Signaling Pathway WP3888
Pathogenic Escherichia coli infection WP2272
Photodynamic therapy-induced unfolded protein response WP3613
Ferroptosis WP4313
Myometrial relaxation and contraction pathways WP289
Mammary gland development pathway - Puberty (Stage 2 of 4) WP2814
Regulation of Actin Cytoskeleton WP51
Focal Adhesion WP306
Androgen receptor signaling pathway WP138
Striated Muscle Contraction Pathway WP383
G13 Signaling Pathway WP524
Prolactin Signaling Pathway WP2037
Calcium Regulation in the Cardiac Cell WP536
Nanoparticle-mediated activation of receptor signaling WP2643
Apoptosis-related network due to altered Notch3 in ovarian cancer WP2864
Spinal Cord Injury WP2431
IL-18 signaling pathway WP4754
Adipogenesis WP236
Prostaglandin Synthesis and Regulation WP98
Gastrin signaling pathway WP4659
Oligodendrocyte specification and differentiation, leading to myelin components for CNS WP4304
Nuclear Receptors Meta-Pathway WP2882
SARS-CoV-2 innate immunity evasion and cell-specific immune response WP5039
-log10(p-adj.)
0.5
1
OA
PA
TGF-β1

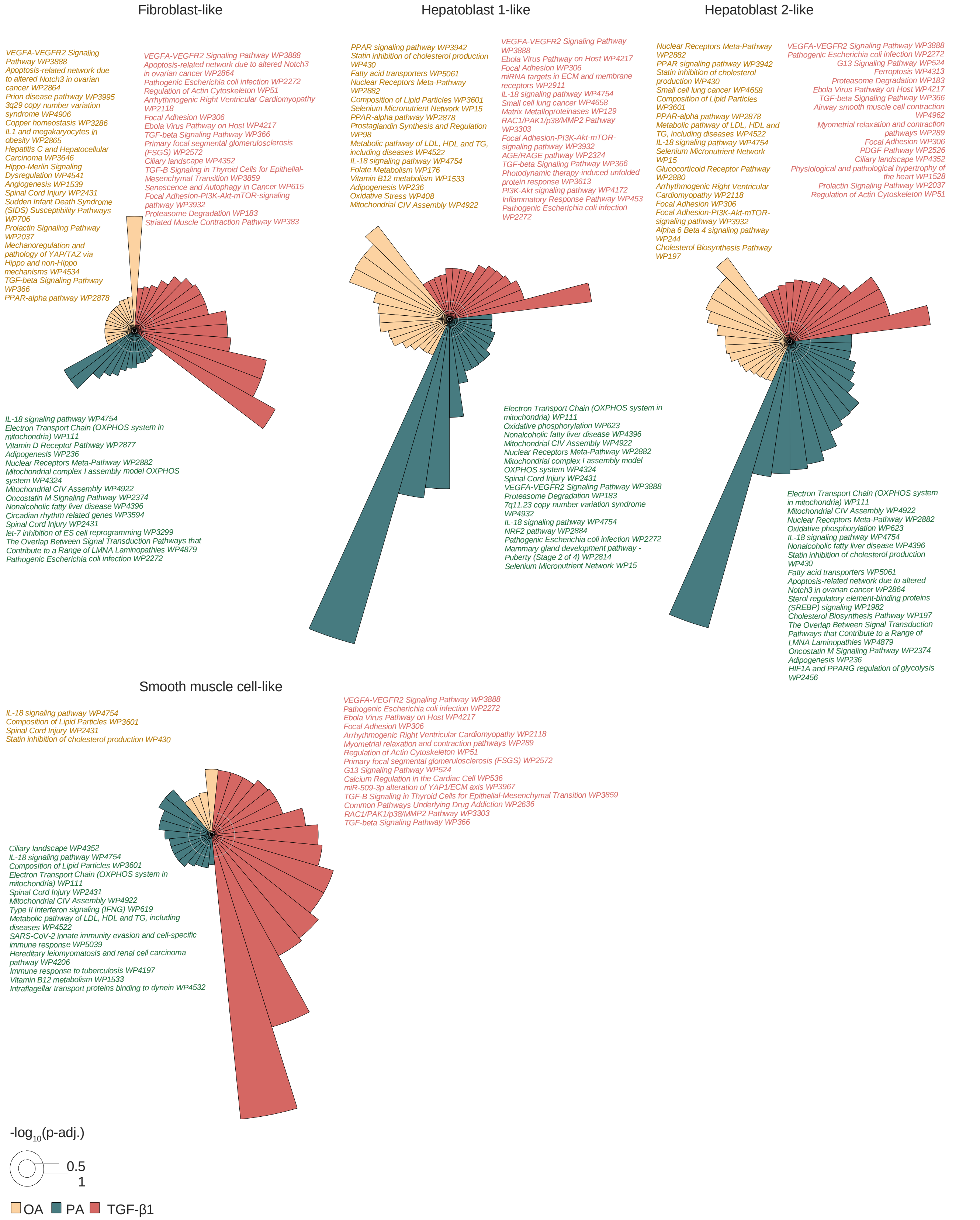
Fibroblast-like
VEGFA-VEGFR2 Signaling Pathway WP3888
Apoptosis-related network due to altered Notch3 in ovarian cancer WP2864
Prion disease pathway WP3995
3q29 copy number variation syndrome WP4906
Copper homeostasis WP3286
IL1 and megakaryocytes in obesity WP2865
Hepatitis C and Hepatocellular Carcinoma WP3646
Hippo-Merlin Signaling Dysregulation WP4541
Angiogenesis WP1539
Spinal Cord Injury WP2431
Sudden Infant Death Syndrome (SIDS) Susceptibility Pathways WP706
Prolactin Signaling Pathway WP2037
Mechanoregulation and pathology of YAP/TAZ via Hippo and non-Hippo mechanisms WP4534
TGF-beta Signaling Pathway WP366
PPAR-alpha pathway WP2878
VEGFA-VEGFR2 Signaling Pathway WP3888
Apoptosis-related network due to altered Notch3 in ovarian cancer WP2864
Pathogenic Escherichia coli infection WP2272
Regulation of Actin Cytoskeleton WP51
Arrhythmogenic Right Ventricular Cardiomyopathy WP2118
Focal Adhesion WP306
Ebola Virus Pathway on Host WP4217
TGF-beta Signaling Pathway WP366
Primary focal segmental glomerulosclerosis (FSGS) WP2572
Ciliary landscape WP4352
TGF-B Signaling in Thyroid Cells for Epithelial-Mesenchymal Transition WP3859
Senescence and Autophagy in Cancer WP615
Focal Adhesion-PI3K-Akt-mTOR-signaling pathway WP3932
Proteasome Degradation WP183
Striated Muscle Contraction Pathway WP383
IL-18 signaling pathway WP4754
Electron Transport Chain (OXPHOS system in mitochondria) WP111
Vitamin D Receptor Pathway WP2877
Adipogenesis WP236
Nuclear Receptors Meta-Pathway WP2882
Mitochondrial complex I assembly model OXPHOS system WP4324
Mitochondrial CIV Assembly WP4922
Oncostatin M Signaling Pathway WP2374
Nonalcoholic fatty liver disease WP4396
Circadian rhythm related genes WP3594
Spinal Cord Injury WP2431
let-7 inhibition of ES cell reprogramming WP3299
The Overlap Between Signal Transduction Pathways that Contribute to a Range of LMNA Laminopathies WP4879
Pathogenic Escherichia coli infection WP2272
Hepatoblast 1-like
PPAR signaling pathway WP3942
Statin inhibition of cholesterol production WP430
Fatty acid transporters WP5061
Nuclear Receptors Meta-Pathway WP2882
Composition of Lipid Particles WP3601
Selenium Micronutrient Network WP15
PPAR-alpha pathway WP2878
Prostaglandin Synthesis and Regulation WP98
Metabolic pathway of LDL, HDL and TG, including diseases WP4522
IL-18 signaling pathway WP4754
Folate Metabolism WP176
Vitamin B12 metabolism WP1533
Adipogenesis WP236
Oxidative Stress WP408
Mitochondrial CIV Assembly WP4922
VEGFA-VEGFR2 Signaling Pathway WP3888
Ebola Virus Pathway on Host WP4217
Focal Adhesion WP306
miRNA targets in ECM and membrane receptors WP2911
IL-18 signaling pathway WP4754
Small cell lung cancer WP4658
Matrix Metalloproteinases WP129
RAC1/PAK1/p38/MMP2 Pathway WP3303
Focal Adhesion-PI3K-Akt-mTOR-signaling pathway WP3932
AGE/RAGE pathway WP2324
TGF-beta Signaling Pathway WP366
Photodynamic therapy-induced unfolded protein response WP3613
PI3K-Akt signaling pathway WP4172
Inflammatory Response Pathway WP453
Pathogenic Escherichia coli infection WP2272
Electron Transport Chain (OXPHOS system in mitochondria) WP111
Oxidative phosphorylation WP623
Nonalcoholic fatty liver disease WP4396
Mitochondrial CIV Assembly WP4922
Nuclear Receptors Meta-Pathway WP2882
Mitochondrial complex I assembly model OXPHOS system WP4324
Spinal Cord Injury WP2431
VEGFA-VEGFR2 Signaling Pathway WP3888
Proteasome Degradation WP183
7q11.23 copy number variation syndrome WP4932
IL-18 signaling pathway WP4754
NRF2 pathway WP2884
Pathogenic Escherichia coli infection WP2272
Mammary gland development pathway - Puberty (Stage 2 of 4) WP2814
Selenium Micronutrient Network WP15
Hepatoblast 2-like
Nuclear Receptors Meta-Pathway WP2882
PPAR signaling pathway WP3942
Statin inhibition of cholesterol production WP430
Small cell lung cancer WP4658
Composition of Lipid Particles WP3601
PPAR-alpha pathway WP2878
Metabolic pathway of LDL, HDL and TG, including diseases WP4522
IL-18 signaling pathway WP4754
Selenium Micronutrient Network WP15
Glucocorticoid Receptor Pathway WP2880
Arrhythmogenic Right Ventricular Cardiomyopathy WP2118
Focal Adhesion WP306
Focal Adhesion-PI3K-Akt-mTOR-signaling pathway WP3932
Alpha 6 Beta 4 signaling pathway WP244
Cholesterol Biosynthesis Pathway WP197
VEGFA-VEGFR2 Signaling Pathway WP3888
Pathogenic Escherichia coli infection WP2272
G13 Signaling Pathway WP524
Ferroptosis WP4313
Proteasome Degradation WP183
Ebola Virus Pathway on Host WP4217
TGF-beta Signaling Pathway WP366
Airway smooth muscle cell contraction WP4962
Myometrial relaxation and contraction pathways WP289
Focal Adhesion WP306
PDGF Pathway WP2526
Ciliary landscape WP4352
Physiological and pathological hypertrophy of the heart WP1528
Prolactin Signaling Pathway WP2037
Regulation of Actin Cytoskeleton WP51
Electron Transport Chain (OXPHOS system in mitochondria) WP111
Mitochondrial CIV Assembly WP4922
Nuclear Receptors Meta-Pathway WP2882
Oxidative phosphorylation WP623
IL-18 signaling pathway WP4754
Nonalcoholic fatty liver disease WP4396
Statin inhibition of cholesterol production WP430
Fatty acid transporters WP5061
Apoptosis-related network due to altered Notch3 in ovarian cancer WP2864
Sterol regulatory element-binding proteins (SREBP) signaling WP1982
Cholesterol Biosynthesis Pathway WP197
The Overlap Between Signal Transduction Pathways that Contribute to a Range of LMNA Laminopathies WP4879
Oncostatin M Signaling Pathway WP2374
Adipogenesis WP236
HIF1A and PPARG regulation of glycolysis WP2456
Smooth muscle cell-like
IL-18 signaling pathway WP4754
Composition of Lipid Particles WP3601
Spinal Cord Injury WP2431
Statin inhibition of cholesterol production WP430
VEGFA-VEGFR2 Signaling Pathway WP3888
Pathogenic Escherichia coli infection WP2272
Ebola Virus Pathway on Host WP4217
Focal Adhesion WP306
Arrhythmogenic Right Ventricular Cardiomyopathy WP2118
Myometrial relaxation and contraction pathways WP289
Regulation of Actin Cytoskeleton WP51
Primary focal segmental glomerulosclerosis (FSGS) WP2572
G13 Signaling Pathway WP524
Calcium Regulation in the Cardiac Cell WP536
miR-509-3p alteration of YAP1/ECM axis WP3967
TGF-B Signaling in Thyroid Cells for Epithelial-Mesenchymal Transition WP3859
Common Pathways Underlying Drug Addiction WP2636
RAC1/PAK1/p38/MMP2 Pathway WP3303
TGF-beta Signaling Pathway WP366
Ciliary landscape WP4352
IL-18 signaling pathway WP4754
Composition of Lipid Particles WP3601
Electron Transport Chain (OXPHOS system in mitochondria) WP111
Spinal Cord Injury WP2431
Mitochondrial CIV Assembly WP4922
Type II interferon signaling (IFNG) WP619
Metabolic pathway of LDL, HDL and TG, including diseases WP4522
SARS-CoV-2 innate immunity evasion and cell-specific immune response WP5039
Hereditary leiomyomatosis and renal cell carcinoma pathway WP4206
Immune response to tuberculosis WP4197
Vitamin B12 metabolism WP1533
Intraflagellar transport proteins binding to dynein WP4532
-log10(p-adj.)
0.5
1
OA
PA
TGF-β1

Appendix Figure S5. WikiPathways[5] enrichment analysis across cell clusters. Corresponding to Fig. 4f.

Circular barplots display the negative decadic logarithm of adjusted *p*-values for WikiPathways[5] terms enriched among differential genes for each condition per cell cluster. Cut-off for plots is an adjusted *p*-value below 0.05.

Fig. S6

a.

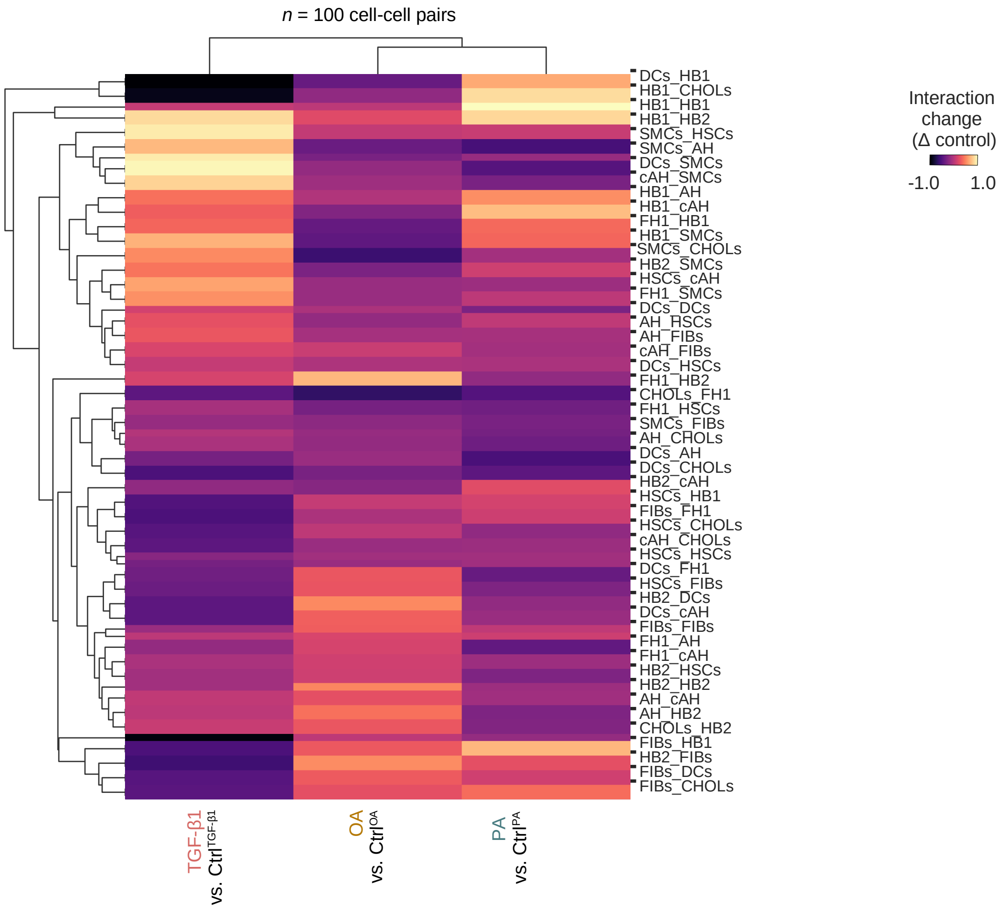

n = 100 cell-cell pairs
DCs_HB1
HB1_CHOLs
HB1_HB1
HB1_HB2
SMCs_HSCs
SMCs_AH
DCs_SMCs
cAH_SMCs
HB1_AH
HB1_cAH
FH1_HB1
HB1_SMCs
SMCs_CHOLs
HB2_SMCs
HSCs_cAH
FH1_SMCs
DCs_DCs
AH_HSCs
AH_FIBs
cAH_FIBs
DCs_HSCs
FH1_HB2
CHOLs_FH1
FH1_HSCs
SMCs_FIBs
AH_CHOLs
DCs_AH
DCs_CHOLs
HB2_cAH
HSCs_HB1
FIBs_FH1
HSCs_CHOLs
cAH_CHOLs
HSCs_HSCs
DCs_FH1
HSCs_FIBs
HB2_DCs
DCs_cAH
FIBs_FIBs
FH1_AH
FH1_cAH
HB2_HSCs
HB2_HB2
AH_cAH
AH_HB2
CHOLs_HB2
FIBs_HB1
HB2_FIBs
FIBs_DCs
FIBs_CHOLs
TGF-β1 vs. Ctrl^TGF-β1
OA vs. Ctrl^OA
PA vs. Ctrl^PA
Interaction change (Δ control)
-1.0
1.0

b.

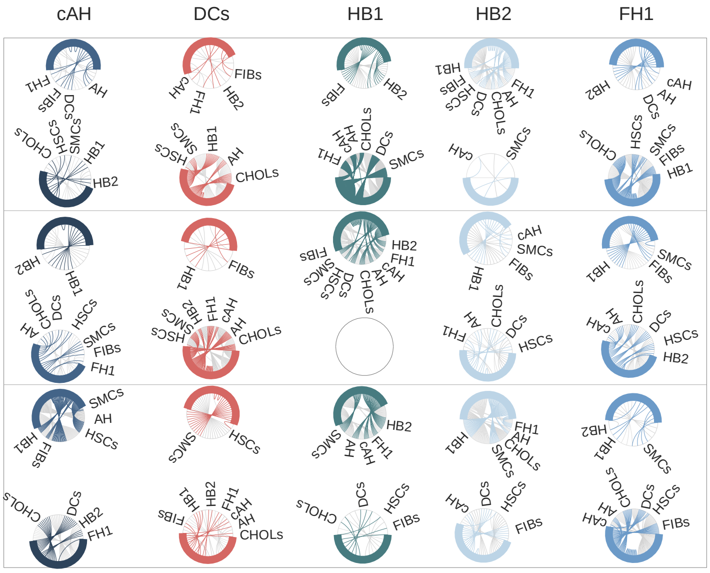

cAH
DCs
HB1
HB2
FH1

Fig. S6 continued

c.

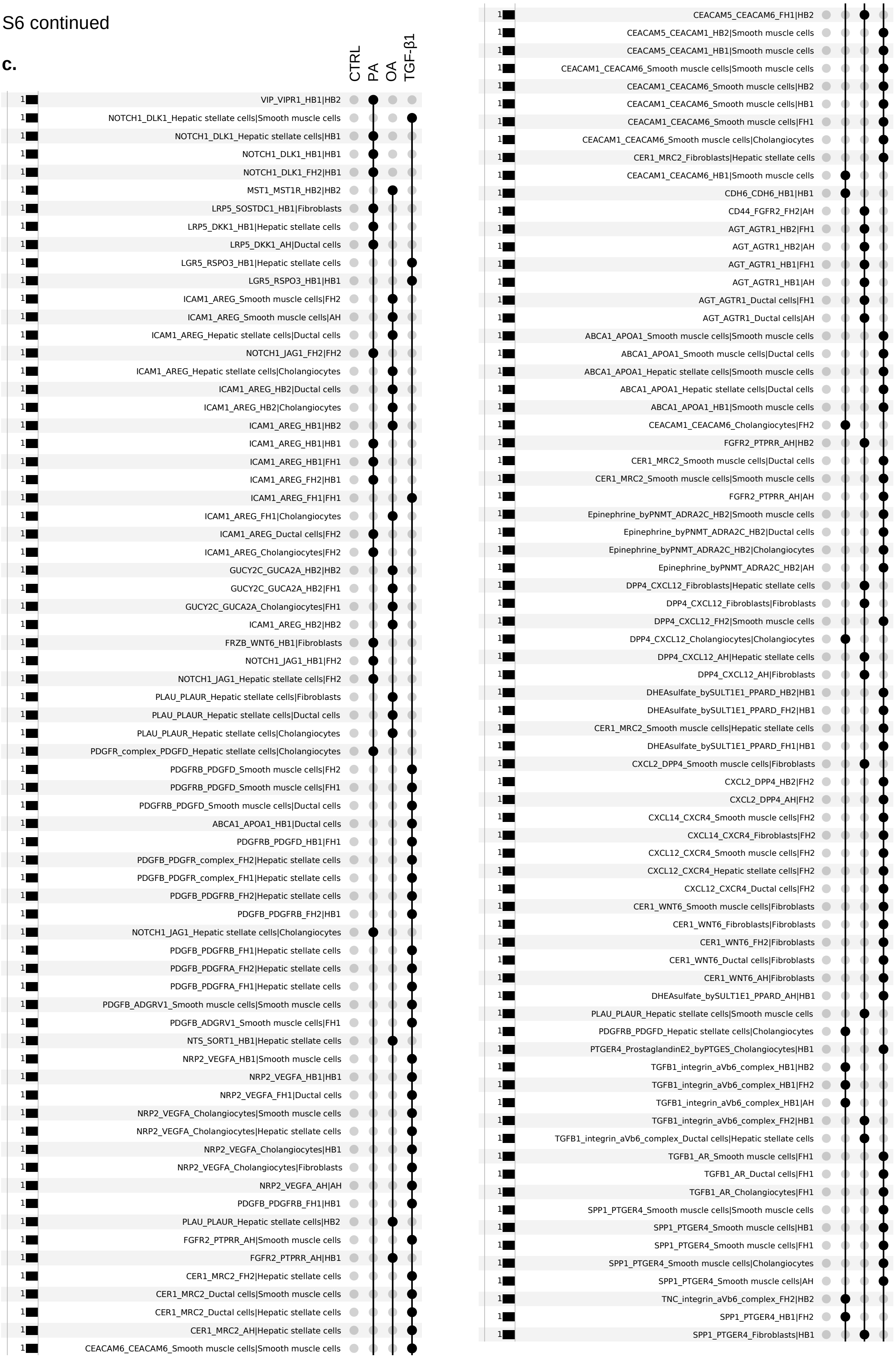

Fig. S6 continued

c.

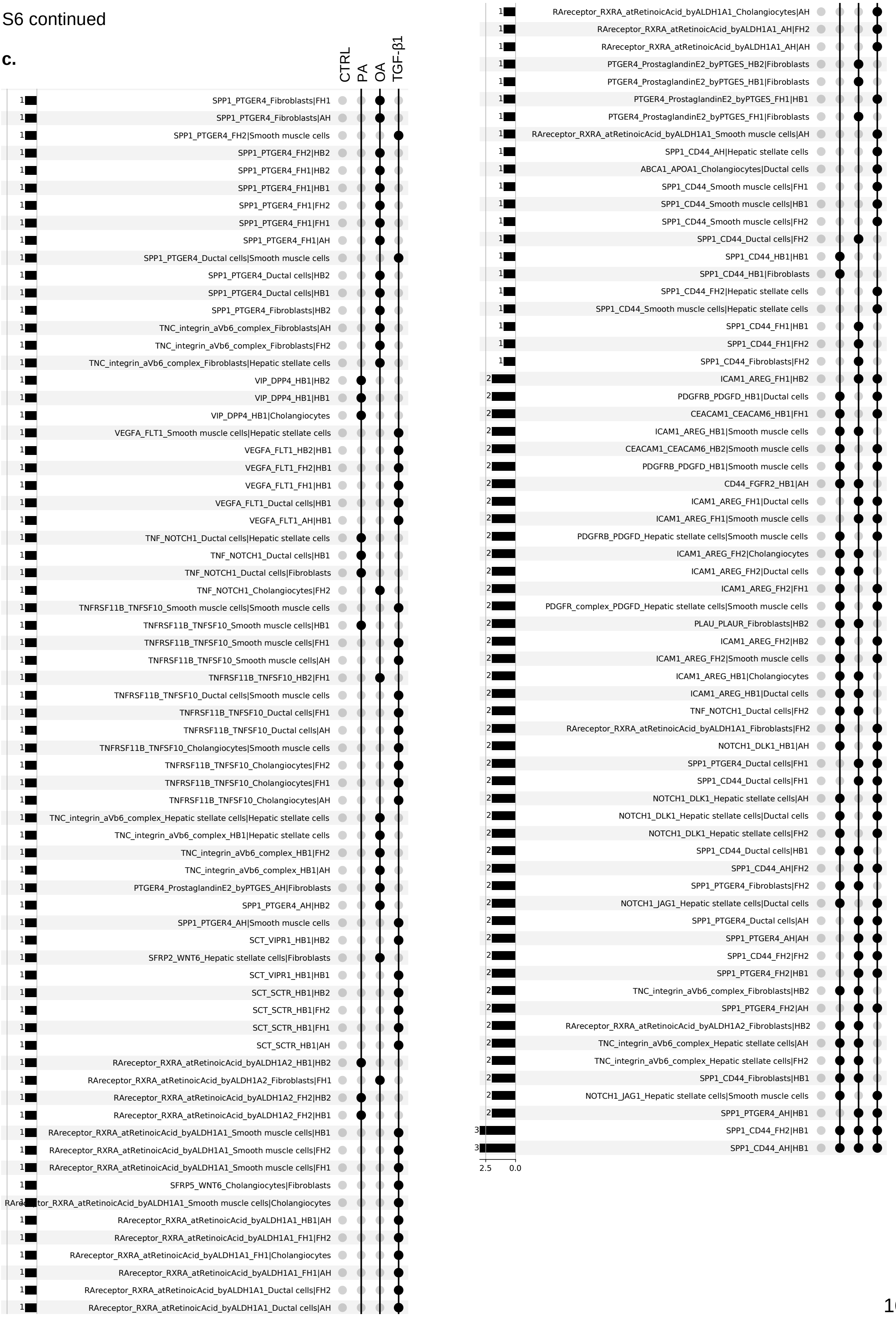

Appendix Figure S6. Overview of differential cell-cell interactions in OS-HLOs upon OA, PA, and TGF-β1 treatment. Corresponding to Fig. 5.

a. Clustered heatmap shows the difference in the fraction of total interactions between each treatment and its control group. Dendrograms show the hierarchical clustering by condition (top) and cell-cell interaction (left, every second interaction is labeled). N = 100 possible cell-cell interactions, n = 2 replicates per condition, n = 6 controls.

b. Chord diagrams showing the fraction of total interactions per source cell with its respective target cells in HLOs treated with OA, PA, or TGF-β1. Chord diagrams are divided by interactions that have i) a higher fraction of total interactions in treatment conditions compared to their corresponding control ("enforced"), and ii) interactions with a lower fraction of total interactions ("reduced"). Colored lines indicate interactions initiated by the source cell in each column, grey lines indicate interactions of other cell types targeting the source cell presented in each chord diagram.

c. Upset plot[6] showing overlapping and specific significant ($p < 0.05$) cell-cell interactions as identified through CellPhoneDB[7] analysis in HLO injury conditions (OA, PA, TGF-β1). Interactions that were also significant in both control and treatment conditions are not displayed. Black dot indicates presence of the significant cell-cell interaction in the condition.

Fig. S7

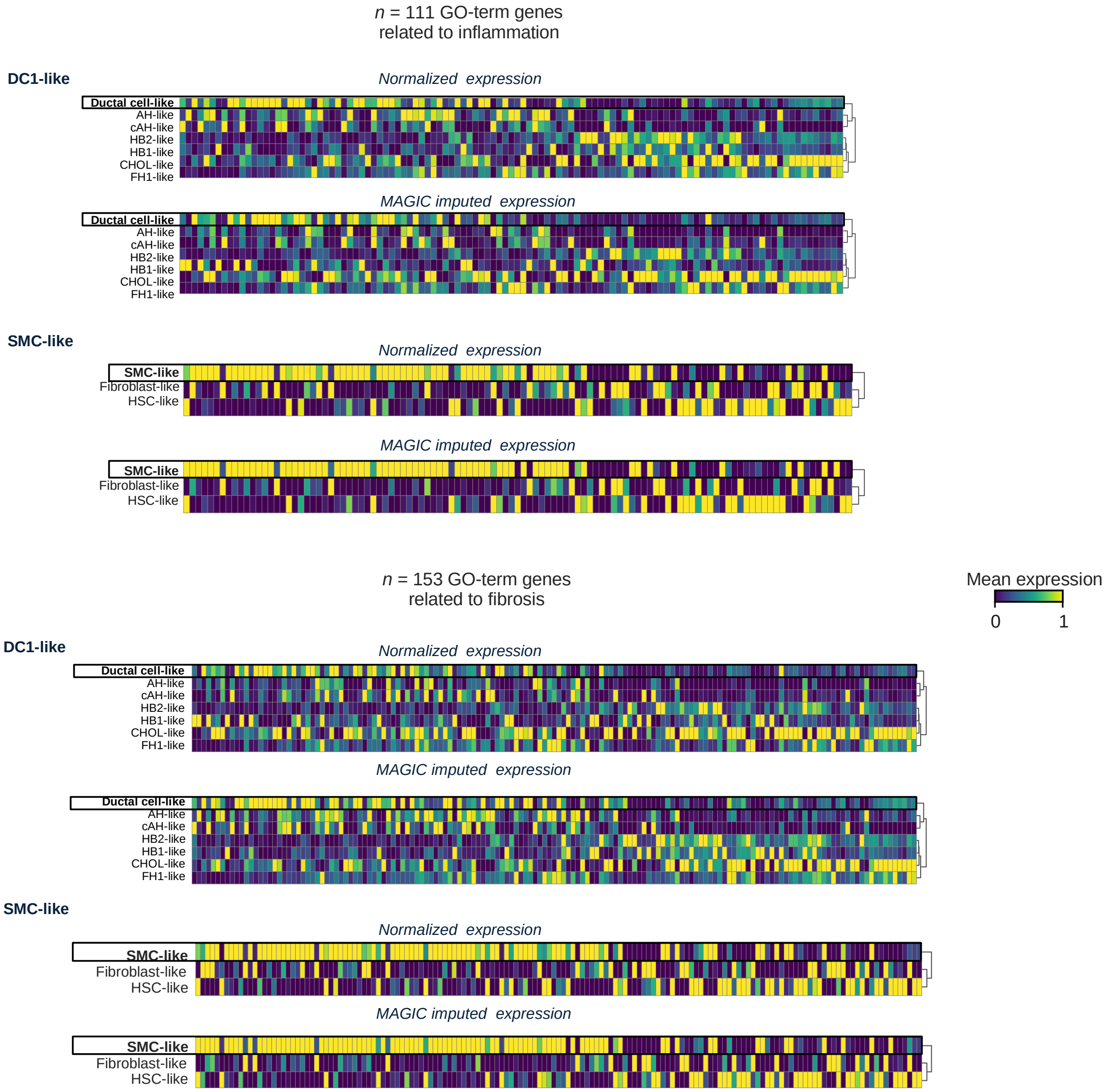
n = 111 GO-term genes
related to inflammation
DC1-like
Normalized expression
Ductal cell-like
AH-like
cAH-like
HB2-like
HB1-like
CHOL-like
FH1-like
MAGIC imputed expression
Ductal cell-like
AH-like
cAH-like
HB2-like
HB1-like
CHOL-like
FH1-like
SMC-like
Normalized expression
SMC-like
Fibroblast-like
HSC-like
MAGIC imputed expression
SMC-like
Fibroblast-like
HSC-like
n = 153 GO-term genes
related to fibrosis
Mean expression
0
1
DC1-like
Normalized expression
Ductal cell-like
AH-like
cAH-like
HB2-like
HB1-like
CHOL-like
FH1-like
MAGIC imputed expression
Ductal cell-like
AH-like
cAH-like
HB2-like
HB1-like
CHOL-like
FH1-like
SMC-like
Normalized expression
SMC-like
Fibroblast-like
HSC-like
MAGIC imputed expression
SMC-like
Fibroblast-like
HSC-like

Appendix Figure S7. Comparison of normalized expression and imputed expression for two major terminal states in OS-HLOs.

a. Projection of the top 100 terminal branch cells (sorted by branch probabilities) for two terminal states identified by Palantir on the ForceAtlas2 representation (top). Corresponding barplots below displaying the relative distribution of treatment conditions among the top 100 terminal branch cells for each terminal state.

b. Heatmaps showing the imputed gene expression over Palantir pseudotime for GO-term-derived inflammation (left) and fibrosis (right)-related genes sorted by their imputed expression level at each terminal state (indicated on top of each heatmap). Imputed expression levels are indicated by color, y-axes correspond to genes, and x-axes display the pseudotime. Ranked gene lists are provided in Dataset EV8.

c. Matrix plots displaying standard normalized mean expression (top) along with imputed gene expression (MAGIC, bottom) for genes identified to enrich towards pseudotime for the DC1- and SMC-like terminal states (genes on the x-axis are the same as in y-axis of b). Categorical ordering by cell type. Dendrogram on the right displaying hierarchical clustering.

Fig. S8

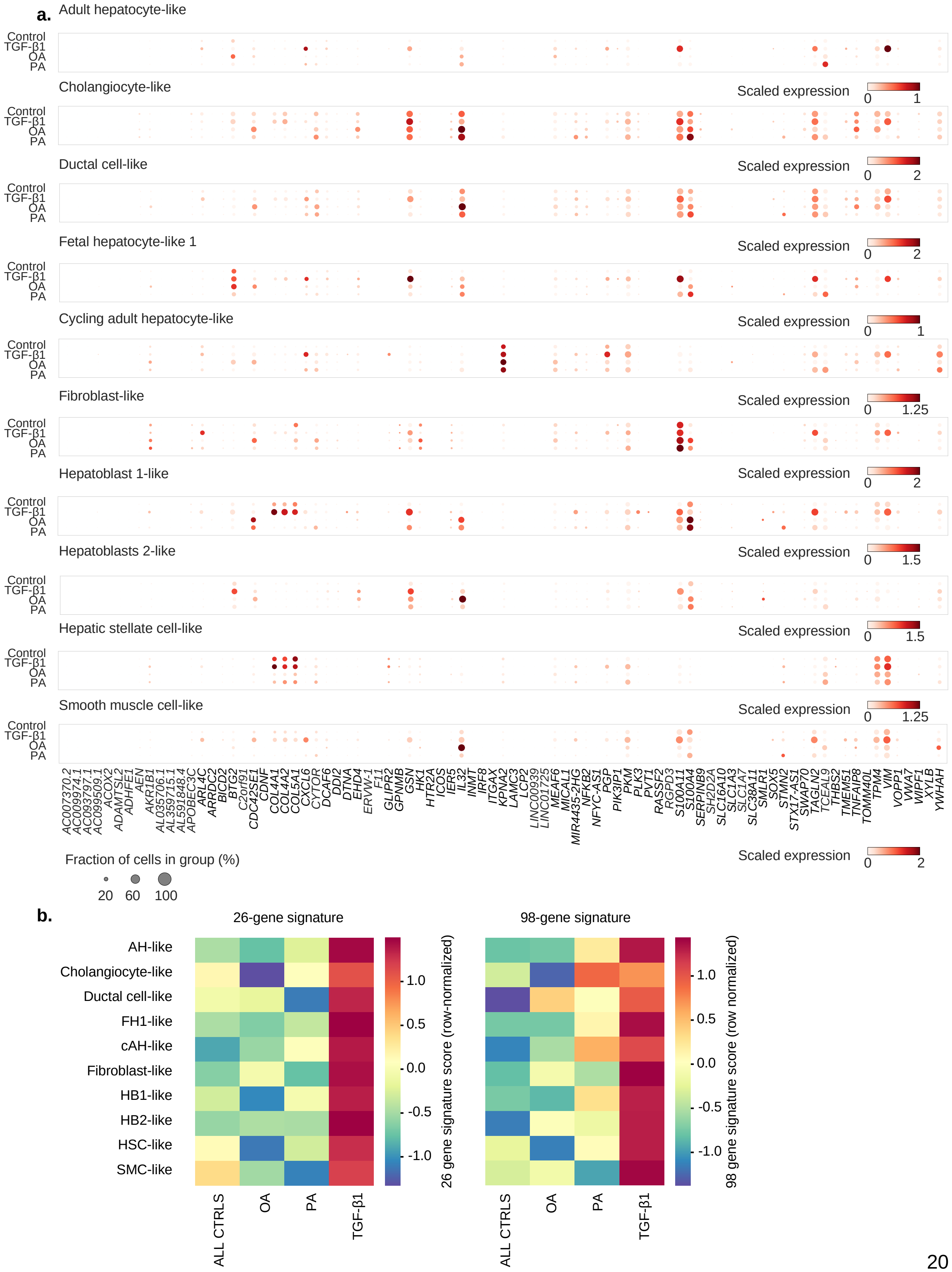
a.
Adult hepatocyte-like
Control
TGF-β1
OA
PA
Cholangiocyte-like
Scaled expression
0
1
Ductal cell-like
Scaled expression
0
2
Fetal hepatocyte-like 1
Scaled expression
0
2
Cycling adult hepatocyte-like
Scaled expression
0
1
Fibroblast-like
Scaled expression
0
1.25
Hepatoblast 1-like
Scaled expression
0
2
Hepatoblasts 2-like
Scaled expression
0
1.5
Hepatic stellate cell-like
Scaled expression
0
1.5
Smooth muscle cell-like
Scaled expression
0
1.25
AC007370.2
AC009974.1
AC093797.1
AC099509.1
ACOX2
ADAMTSL2
ADHFE1
AEN
AKR1B1
AL035706.1
AL359715.1
AL591848.4
APOBEC3C
ARL4C
ARRDC2
BICD2
BTG2
C2orf91
CDC42SE1
CDNF
COL4A1
COL4A2
COL5A1
CXCL6
CYTOR
DCAF6
DDI2
DTNA
EHD4
ERVW-1
F11
GLIPR2
GPNMB
GSN
HK1
HTR2A
ICOS
IER5
IL32
INMT
IRF8
ITGAX
KPNA2
LAMC3
LCP2
LINC00939
LINC01725
MEAF6
MICAL1
MIR4435-2HG
NFKB2
NFYC-AS1
PGP
PIK3IP1
PKM
PLK3
PVT1
RASSF2
RGPD3
S100A11
S100A4
SERPINB9
SH2D2A
SLC16A10
SLC1A3
SLC1A7
SLC38A11
SMLR1
SOX5
STMN2
STX17-AS1
SWAP70
TAGLN2
TCEAL9
THBS2
TMEM51
TNFAIP8
TOMM40L
TPM4
VIM
VOPP1
VWA7
WIPF1
XYLB
YWHAH
Fraction of cells in group (%)
20
60
100
Scaled expression
0
2
b.
26-gene signature
98-gene signature
AH-like
Cholangiocyte-like
Ductal cell-like
FH1-like
cAH-like
Fibroblast-like
HB1-like
HB2-like
HSC-like
SMC-like
1.0
0.5
0.0
-0.5
-1.0
26 gene signature score (row-normalized)
98 gene signature score (row normalized)
ALL CTRLS
OA
PA
TGF-β1

Appendix Figure S8. Expression of cell type- and injury-specific candidate genes composing signatures predicting fibrosis stages in MASLD[8] gradually change from healthy to OA-, to PA-, to TGF-β1-treated HLOs. Corresponding to Fig. 7c.

a. Dot plots showing scaled expression values for genes from the 98-gene signature to predict fibrosis stages in a cohort of 143 patients across different stages of MASLD[8]. Each panel shows an individual cell type. List has been reduced to genes present among all replicates (n = 8 controls, n = 2 OA, n = 2 PA, n = 2 TGF-β1). Dot size corresponds to the fraction of cells in a group expressing the respective gene, and color indicates the scaled expression level.

b. Heatmaps showing the row-normalized 26- and 98-gene signature scores from a. per cell type and treatment condition.

References

1. Domínguez Conde, C. et al. Cross-tissue immune cell analysis reveals tissue-specific features in humans. Science 376, eabl5197 (2022).

2. Wesley, B. T. et al. Single-cell atlas of human liver development reveals pathways directing hepatic cell fates. Nat Cell Biol 1–12 (2022) doi:10.1038/s41556-022-00989-7.

3. Ianevski, A., Giri, A.K., and Aittokallio, T. (2022). Fully-automated and ultra-fast cell-type identification using specific marker combinations from single-cell transcriptomic data. Nat Commun 13, 1246. 10.1038/s41467-022-28803-w.

4. Kuleshov, M.V., Jones, M.R., Rouillard, A.D., Fernandez, N.F., Duan, Q., Wang, Z., Koplev, S., Jenkins, S.L., Jagodnik, K.M., Lachmann, A., et al. (2016). Enrichr: a comprehensive gene set enrichment analysis web server 2016 update. Nucleic acids research 44, W90–W97. 10.1093/nar/gkw377

5. Martens, M., Ammar, A., Riutta, A., Waagmeester, A., Slenter, D.N., Hanspers, K., A. Miller, R., Digles, D., Lopes, E.N., Ehrhart, F., et al. (2021). WikiPathways: connecting communities. Nucleic Acids Research 49, D613–D621. 10.1093/nar/gkaa1024.

6. Lex, A., Gehlenborg, N., Strobelt, H., Vuillemot, R. & Pfister, H. UpSet: Visualization of Intersecting Sets. IEEE Transactions on Visualization and Computer Graphics 20, 1983–1992 (2014).

7. Efremova, M., Vento-Tormo, M., Teichmann, S. A. & Vento-Tormo, R. CellPhoneDB: inferring cell–cell communication from combined expression of multi-subunit ligand–receptor complexes. Nature Protocols 15, 1484–1506 (2020).

8. Pantano, L., Agyapong, G., Shen, Y., Zhuo, Z., Fernandez-Albert, F., Rust, W., Knebel, D., Hill, J., Boustany-Kari, C.M., Doerner, J.F., et al. (2021). Molecular characterization and cell type composition deconvolution of fibrosis in NAFLD. Scientific Reports 11, 1–14. 10.1038/s41598-021-96966-5.
