# Supplementary figures and images for: Single‐cell transcriptomics stratifies organoid models of metabolic dysfunction‐associated steatotic liver disease
Source: EMBO J. 2023 Nov 14;42(24):e113898. doi: 10.15252/embj.2023113898 (PMC10711666; doi:10.15252/embj.2023113898)

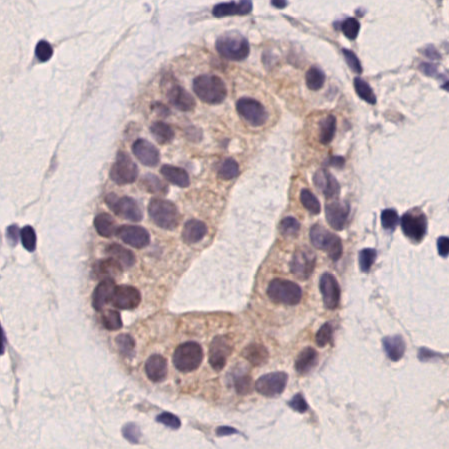

Supplement: Supplementary file 13 — Source Data for Figure 1 [file EMBJ-42-e113898-s009.zip › Figure_1/1C/CEBPA.tif]

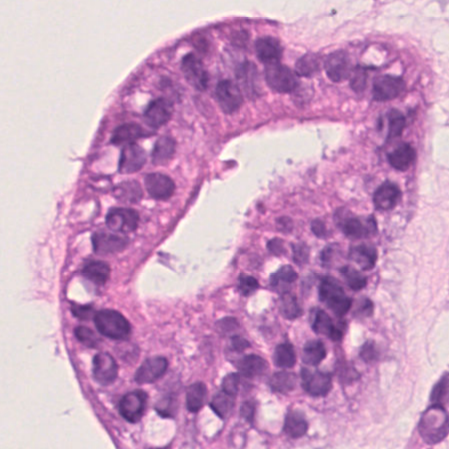

Supplement: Supplementary file 13 — Source Data for Figure 1 [file EMBJ-42-e113898-s009.zip › Figure_1/1C/HE.tif]

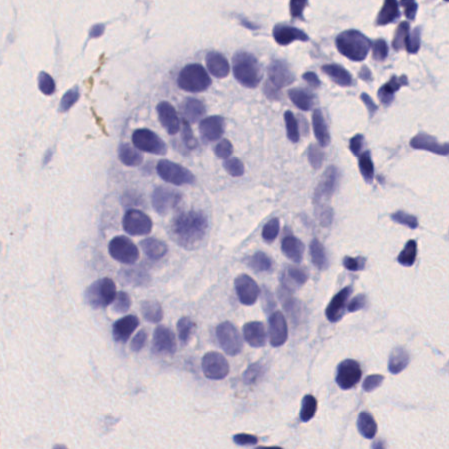

Supplement: Supplementary file 13 — Source Data for Figure 1 [file EMBJ-42-e113898-s009.zip › Figure_1/1C/secAB_negctrl.tif]

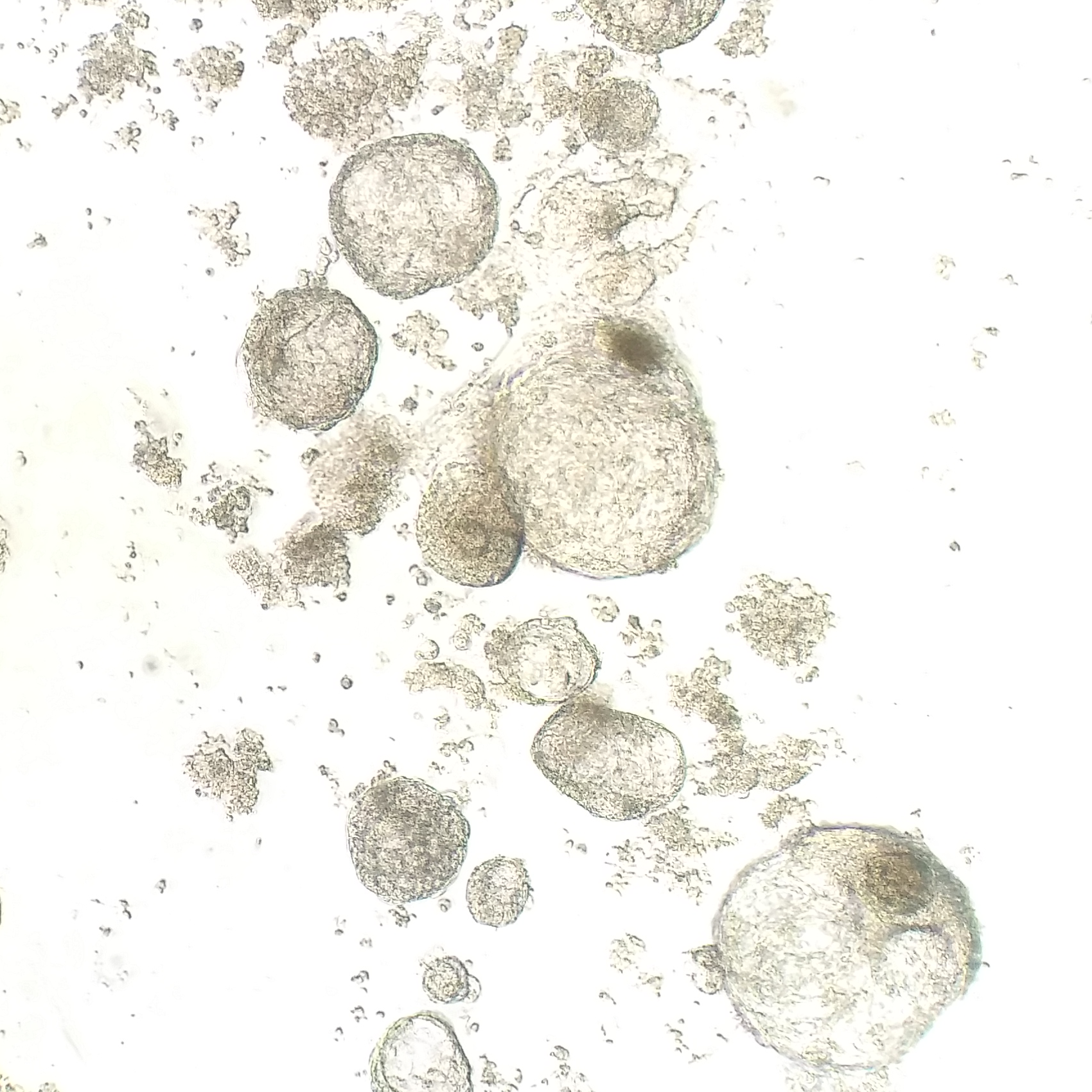

Supplement: Supplementary file 14 — Source Data for Figure 3 [file EMBJ-42-e113898-s007.zip › Figure_3/3B/OS_CTRL.tif]

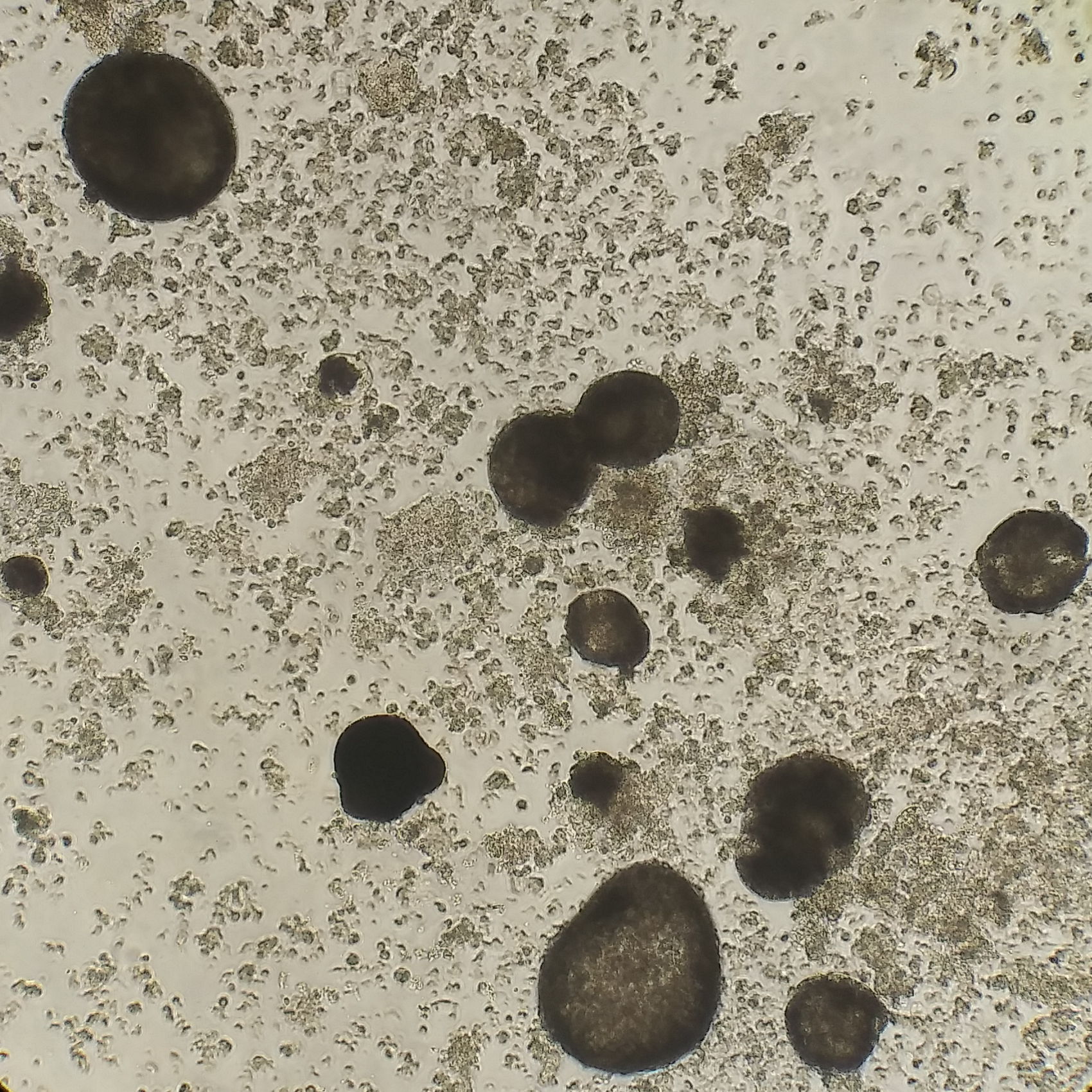

Supplement: Supplementary file 14 — Source Data for Figure 3 [file EMBJ-42-e113898-s007.zip › Figure_3/3B/OS_OA.tif]

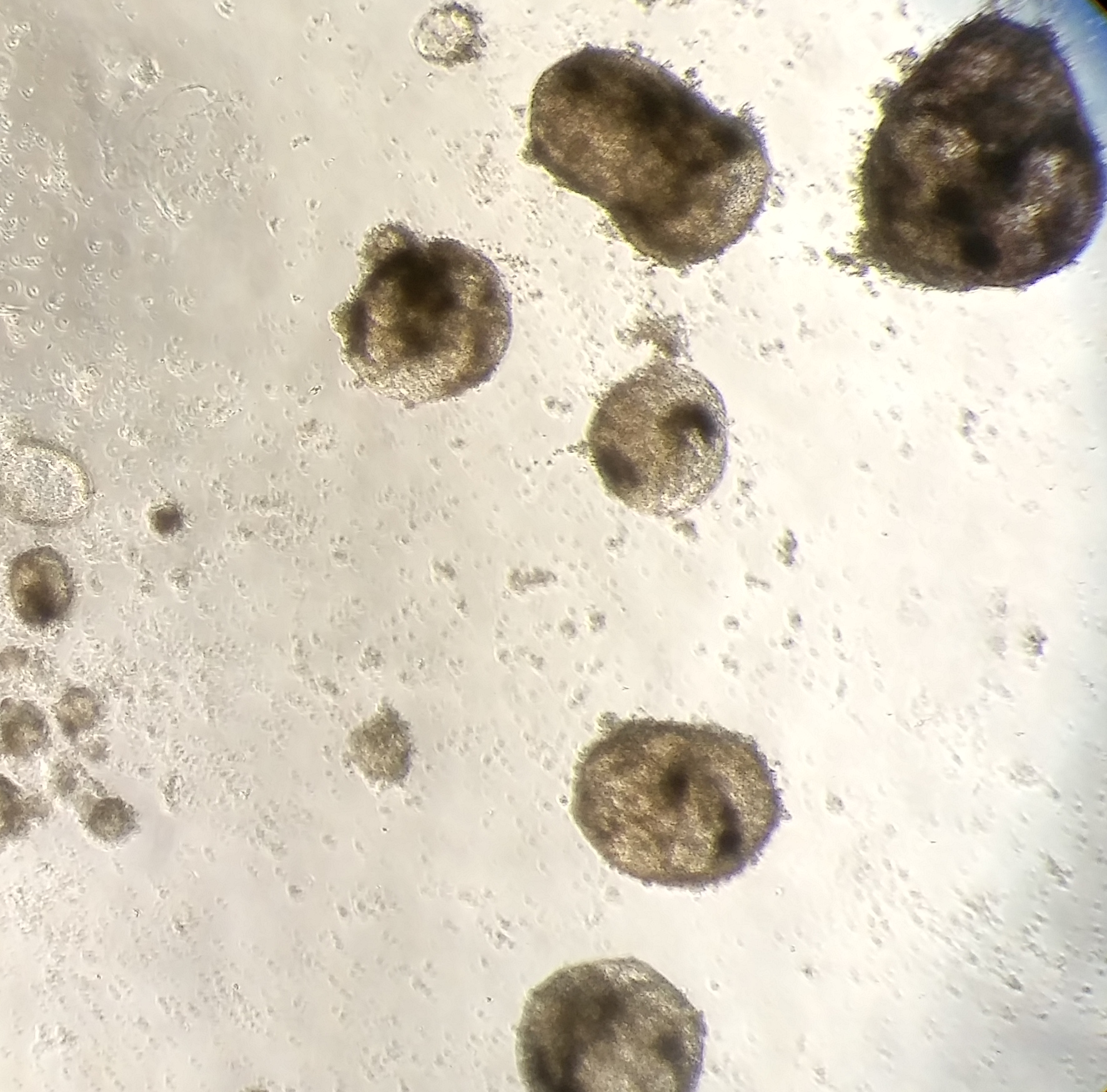

Supplement: Supplementary file 14 — Source Data for Figure 3 [file EMBJ-42-e113898-s007.zip › Figure_3/3B/OS_TGFB1-10ng-ml.tif]

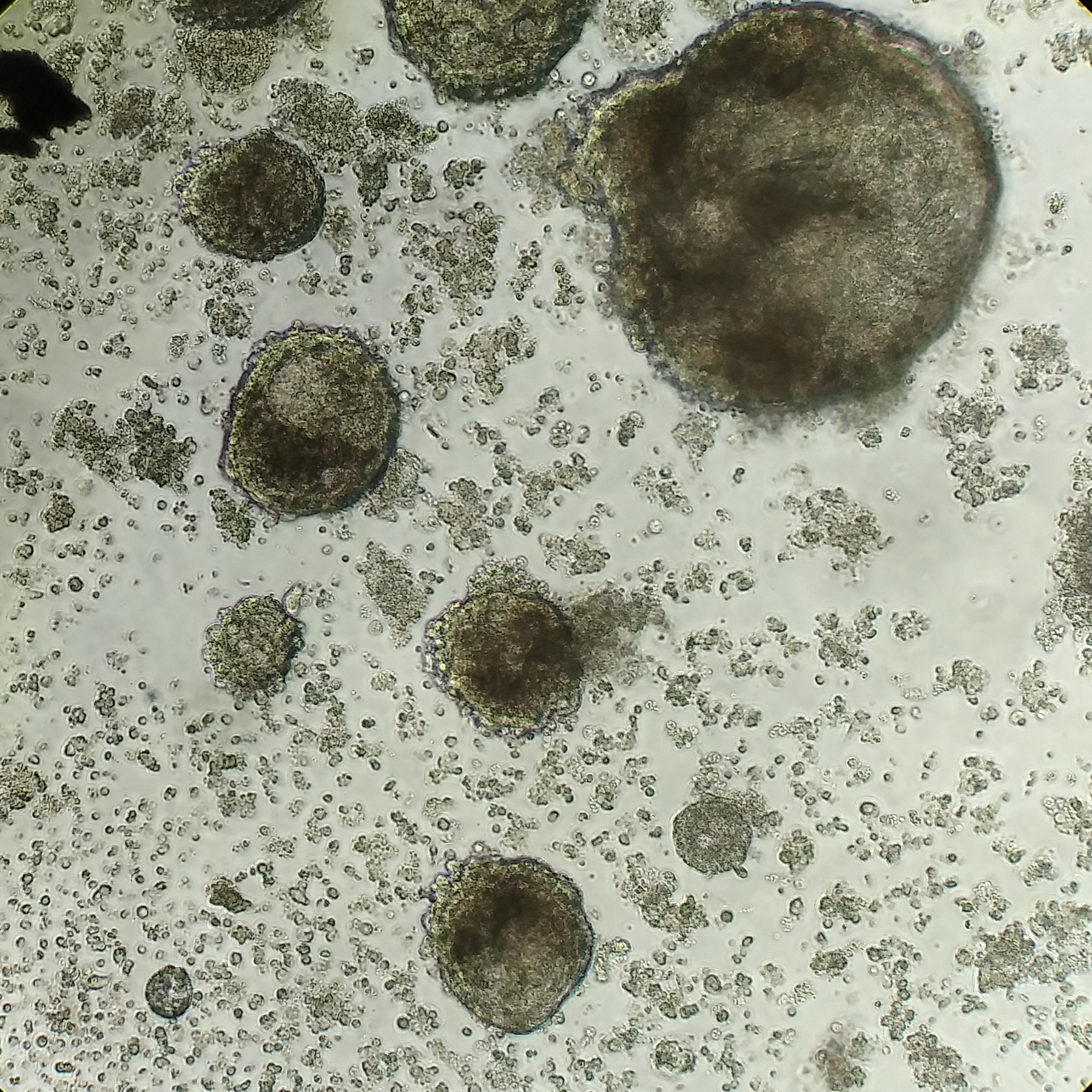

Supplement: Supplementary file 14 — Source Data for Figure 3 [file EMBJ-42-e113898-s007.zip › Figure_3/3B/OS_TGFB1-25ng-ml.tif]

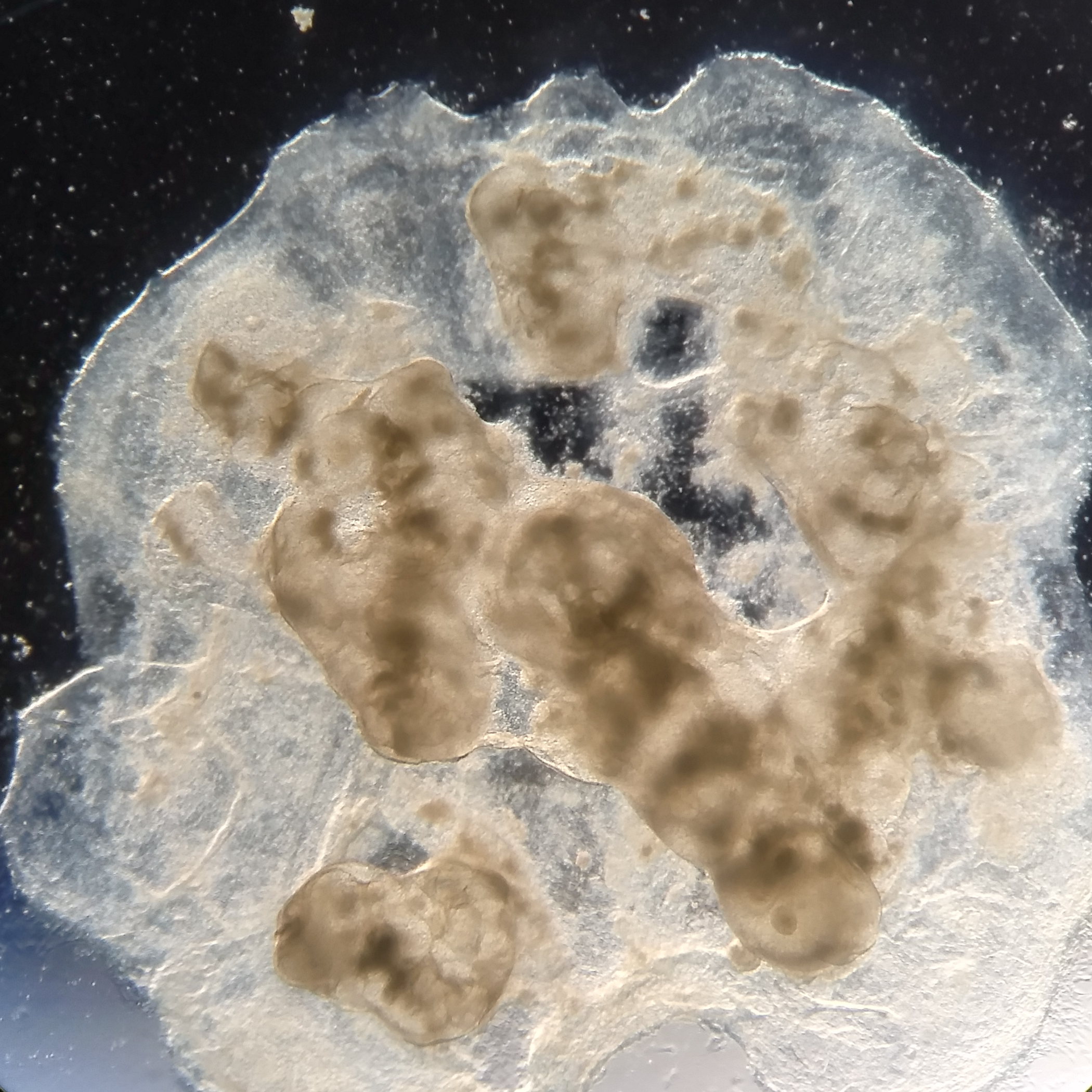

Supplement: Supplementary file 14 — Source Data for Figure 3 [file EMBJ-42-e113898-s007.zip › Figure_3/3B/MG_CTRL.tif]

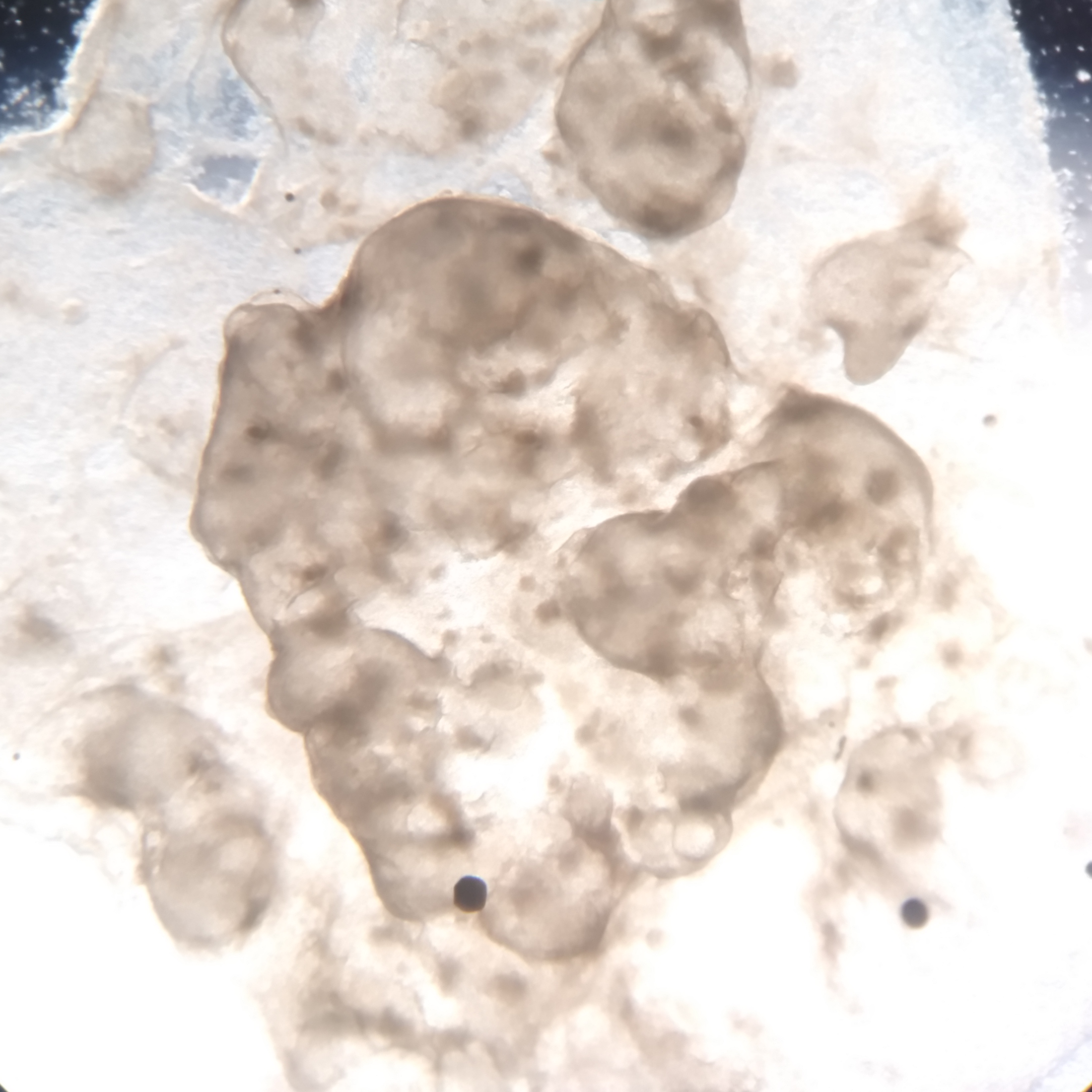

Supplement: Supplementary file 14 — Source Data for Figure 3 [file EMBJ-42-e113898-s007.zip › Figure_3/3B/MG_OA.tif]

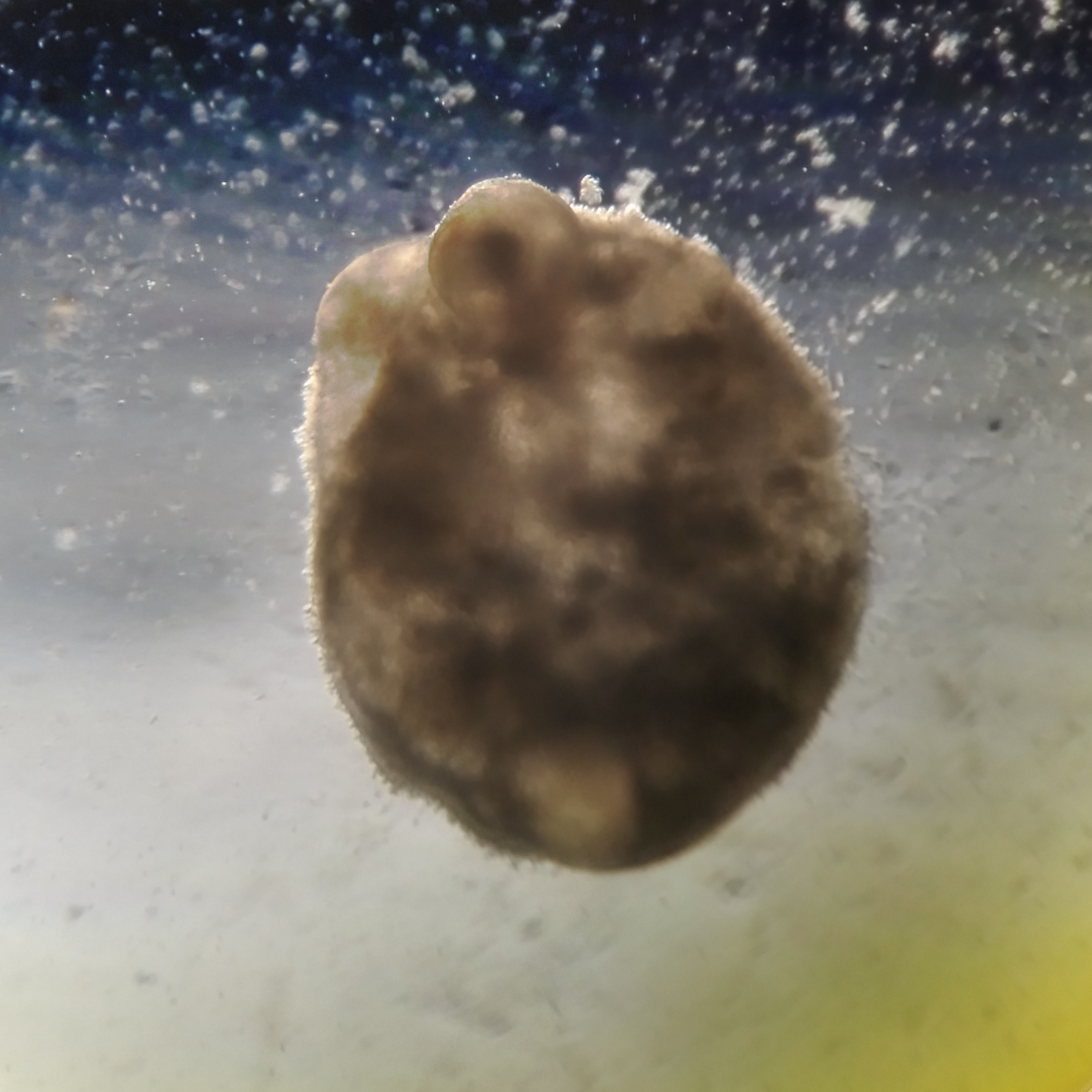

Supplement: Supplementary file 14 — Source Data for Figure 3 [file EMBJ-42-e113898-s007.zip › Figure_3/3B/MG_TGFB1-10ng-ml.tif]

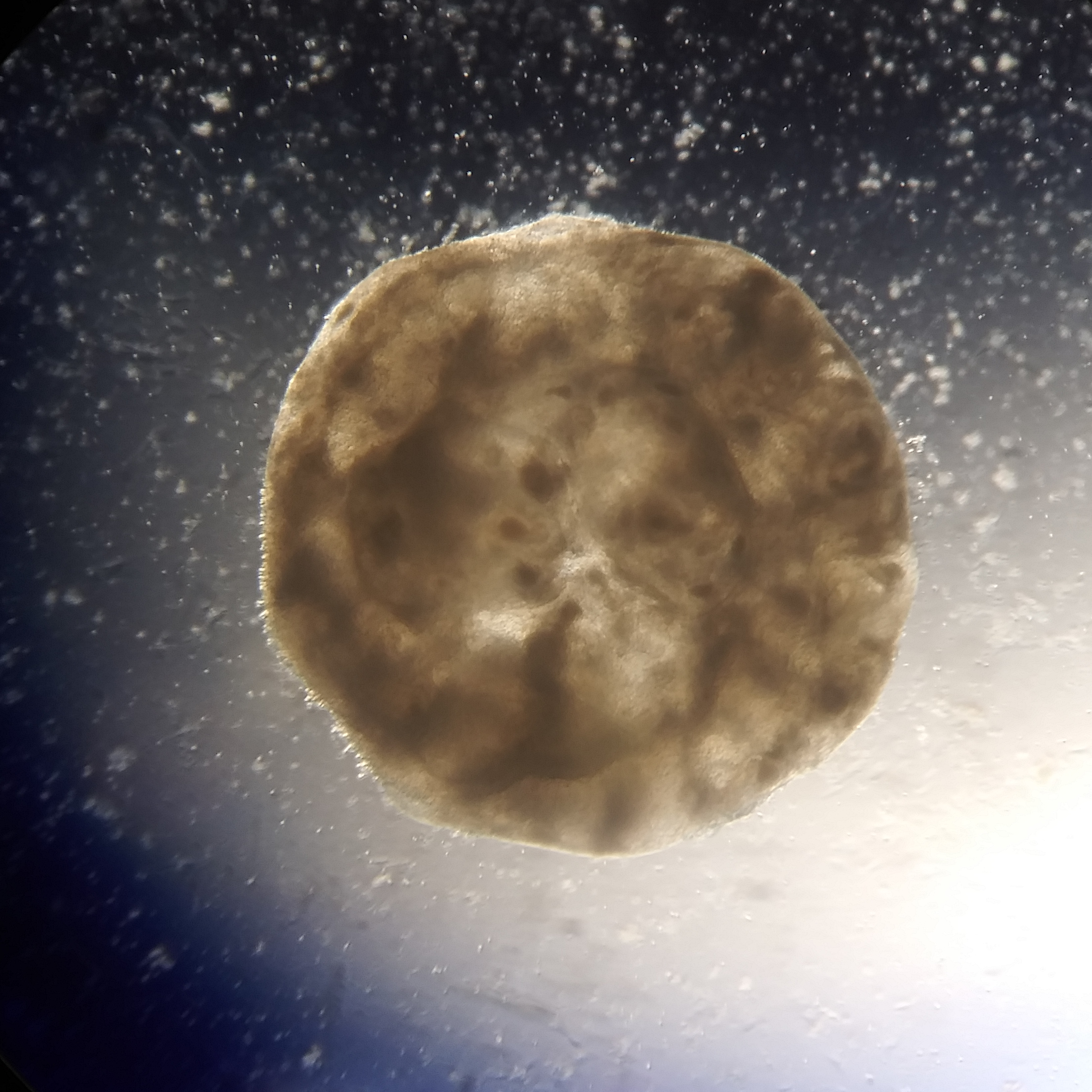

Supplement: Supplementary file 14 — Source Data for Figure 3 [file EMBJ-42-e113898-s007.zip › Figure_3/3B/MG_TGFB1-25ng-ml.tif]

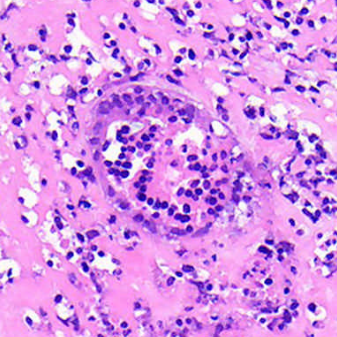

Supplement: Supplementary file 14 — Source Data for Figure 3 [file EMBJ-42-e113898-s007.zip › Figure_3/3D/Matrigel_TGFB10.tif]

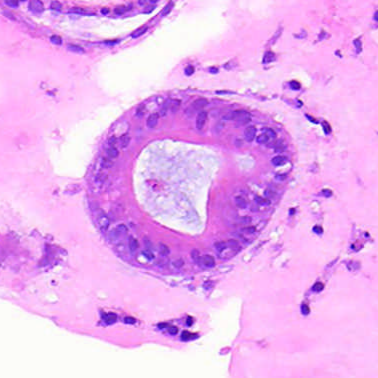

Supplement: Supplementary file 14 — Source Data for Figure 3 [file EMBJ-42-e113898-s007.zip › Figure_3/3D/Matrigel_Control.tif]

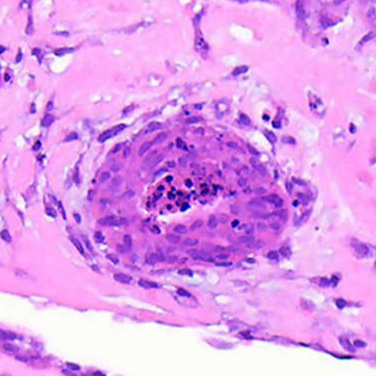

Supplement: Supplementary file 14 — Source Data for Figure 3 [file EMBJ-42-e113898-s007.zip › Figure_3/3D/Matrigel_TGFB25.tif]

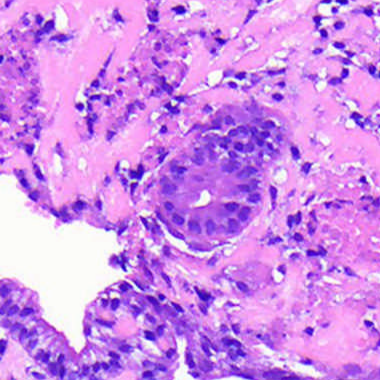

Supplement: Supplementary file 14 — Source Data for Figure 3 [file EMBJ-42-e113898-s007.zip › Figure_3/3D/OS_Control.tif]

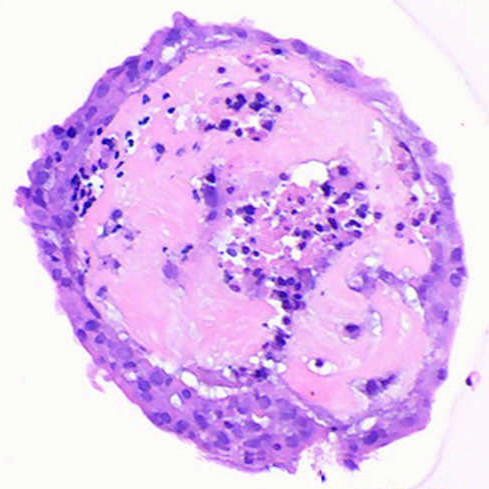

Supplement: Supplementary file 14 — Source Data for Figure 3 [file EMBJ-42-e113898-s007.zip › Figure_3/3D/OS_TGFB25.tif]

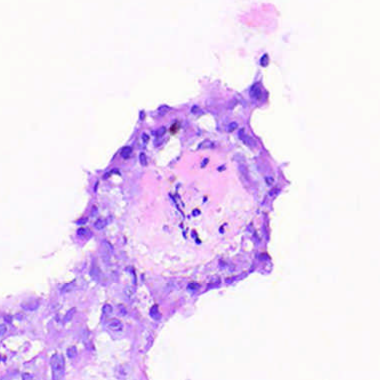

Supplement: Supplementary file 14 — Source Data for Figure 3 [file EMBJ-42-e113898-s007.zip › Figure_3/3D/OS_TGFB10.tif]

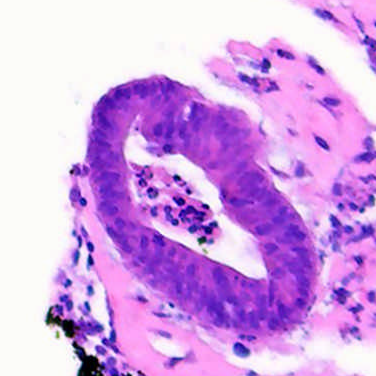

Supplement: Supplementary file 14 — Source Data for Figure 3 [file EMBJ-42-e113898-s007.zip › Figure_3/3D/ULA_Control.tif]

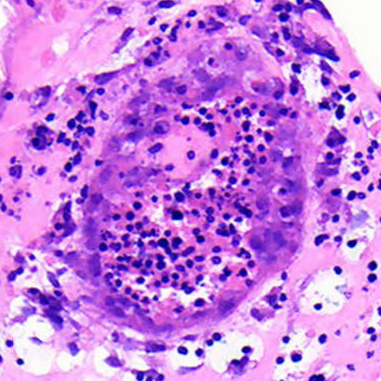

Supplement: Supplementary file 14 — Source Data for Figure 3 [file EMBJ-42-e113898-s007.zip › Figure_3/3D/ULA_TGFB10.tif]

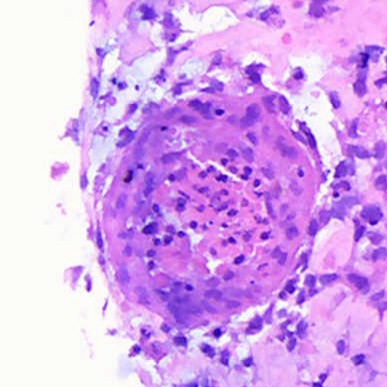

Supplement: Supplementary file 14 — Source Data for Figure 3 [file EMBJ-42-e113898-s007.zip › Figure_3/3D/ULA_TGFB25.tif]

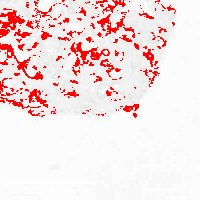

Supplement: Supplementary file 14 — Source Data for Figure 3 [file EMBJ-42-e113898-s007.zip › Figure_3/3E/ULA_TGFB1.tif]

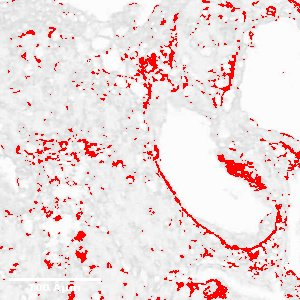

Supplement: Supplementary file 14 — Source Data for Figure 3 [file EMBJ-42-e113898-s007.zip › Figure_3/3E/ULA_Control.tif]

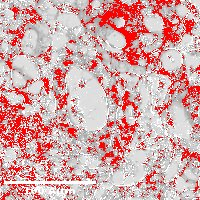

Supplement: Supplementary file 14 — Source Data for Figure 3 [file EMBJ-42-e113898-s007.zip › Figure_3/3E/OS_TGFB1.tif]

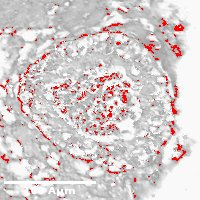

Supplement: Supplementary file 14 — Source Data for Figure 3 [file EMBJ-42-e113898-s007.zip › Figure_3/3E/OS_Control.tif]

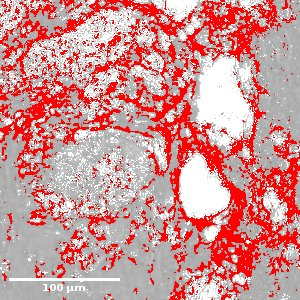

Supplement: Supplementary file 14 — Source Data for Figure 3 [file EMBJ-42-e113898-s007.zip › Figure_3/3E/Matrigel_TGFB1.tif]

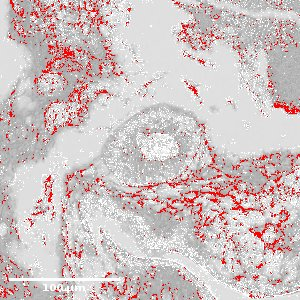

Supplement: Supplementary file 14 — Source Data for Figure 3 [file EMBJ-42-e113898-s007.zip › Figure_3/3E/Matrigel_Control.tif]

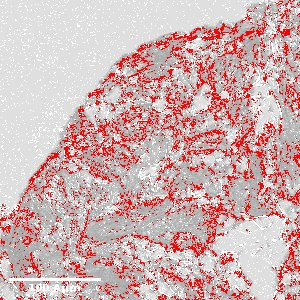

Supplement: Supplementary file 14 — Source Data for Figure 3 [file EMBJ-42-e113898-s007.zip › Figure_3/3E/Agarose_TGFB1.tif]

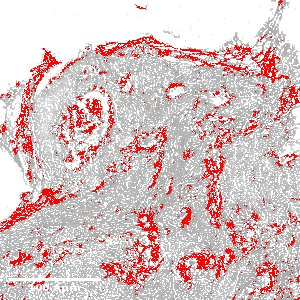

Supplement: Supplementary file 14 — Source Data for Figure 3 [file EMBJ-42-e113898-s007.zip › Figure_3/3E/Agarose_Control.tif]

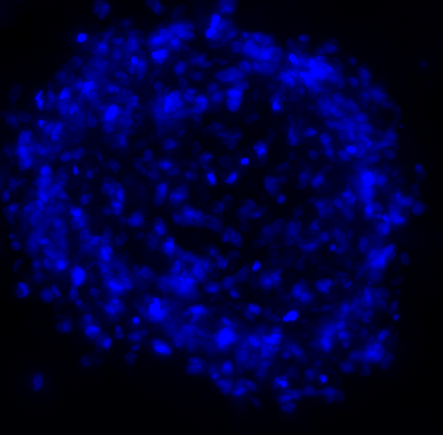

Supplement: Supplementary file 14 — Source Data for Figure 3 [file EMBJ-42-e113898-s007.zip › Figure_3/3H/CT-OA-blue.tif]

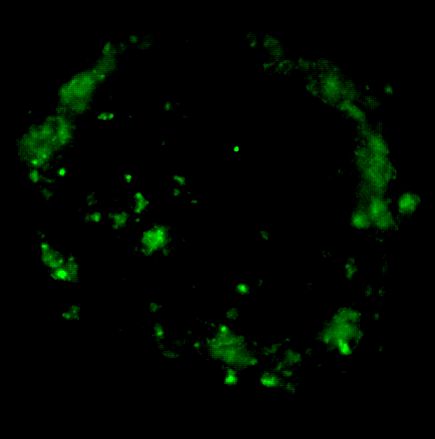

Supplement: Supplementary file 14 — Source Data for Figure 3 [file EMBJ-42-e113898-s007.zip › Figure_3/3H/CT-OA-green.tif]

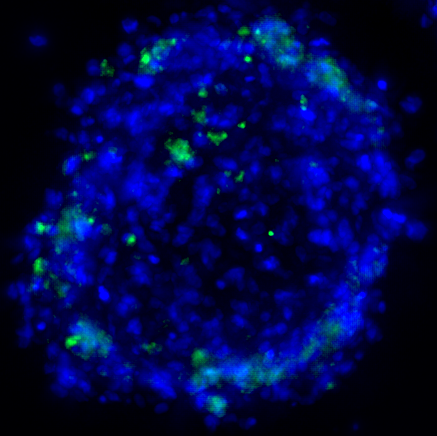

Supplement: Supplementary file 14 — Source Data for Figure 3 [file EMBJ-42-e113898-s007.zip › Figure_3/3H/CT-OA-merge.tif]

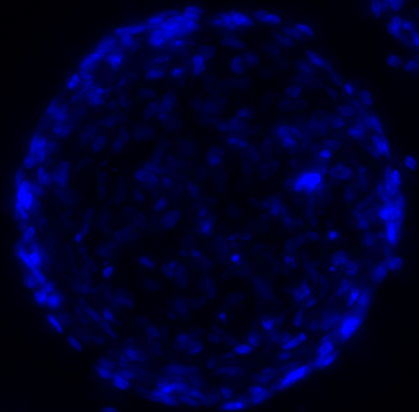

Supplement: Supplementary file 14 — Source Data for Figure 3 [file EMBJ-42-e113898-s007.zip › Figure_3/3H/CT-PA-blue .tif]

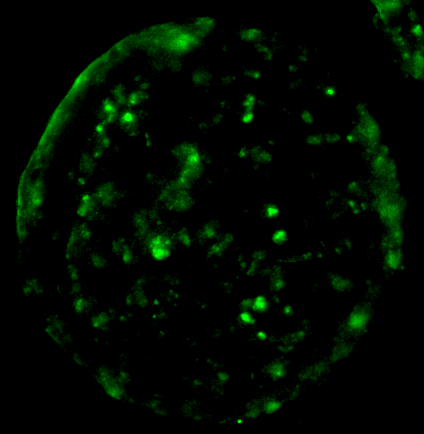

Supplement: Supplementary file 14 — Source Data for Figure 3 [file EMBJ-42-e113898-s007.zip › Figure_3/3H/CT-PA-green.tif]

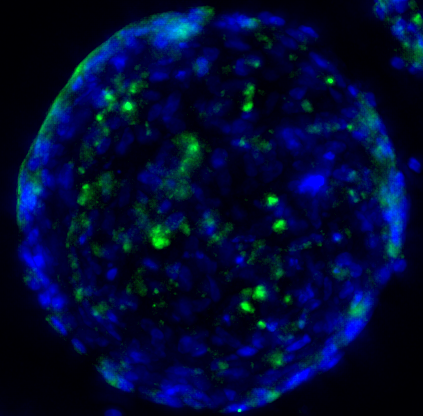

Supplement: Supplementary file 14 — Source Data for Figure 3 [file EMBJ-42-e113898-s007.zip › Figure_3/3H/CT-PA-merge.tif]

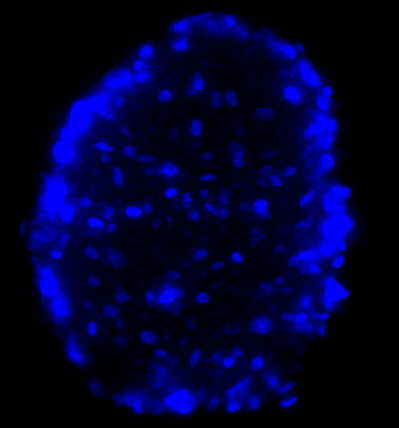

Supplement: Supplementary file 14 — Source Data for Figure 3 [file EMBJ-42-e113898-s007.zip › Figure_3/3H/ct-TGF-blue.tif]

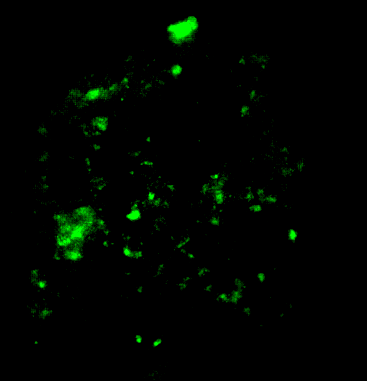

Supplement: Supplementary file 14 — Source Data for Figure 3 [file EMBJ-42-e113898-s007.zip › Figure_3/3H/ct-TGF-green.tif]

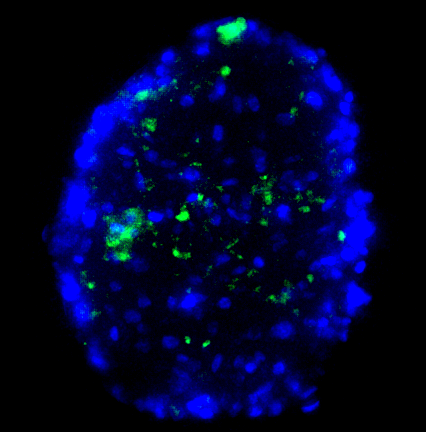

Supplement: Supplementary file 14 — Source Data for Figure 3 [file EMBJ-42-e113898-s007.zip › Figure_3/3H/ct-TGF-merge.tif]

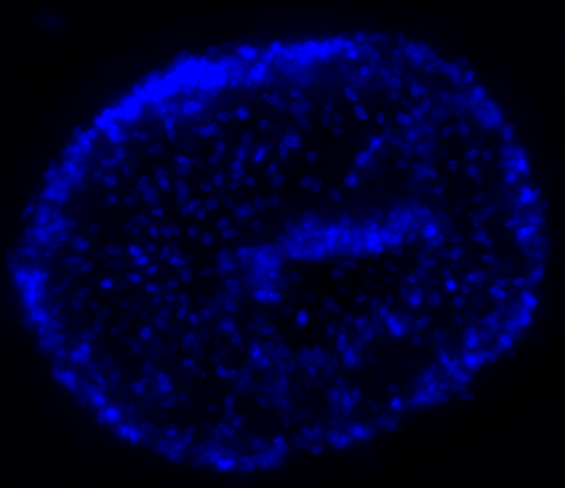

Supplement: Supplementary file 14 — Source Data for Figure 3 [file EMBJ-42-e113898-s007.zip › Figure_3/3H/oa-blue.tif]

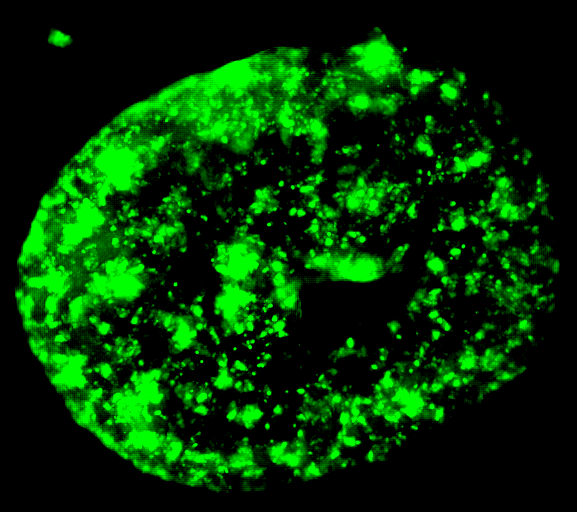

Supplement: Supplementary file 14 — Source Data for Figure 3 [file EMBJ-42-e113898-s007.zip › Figure_3/3H/oa-green.tif]

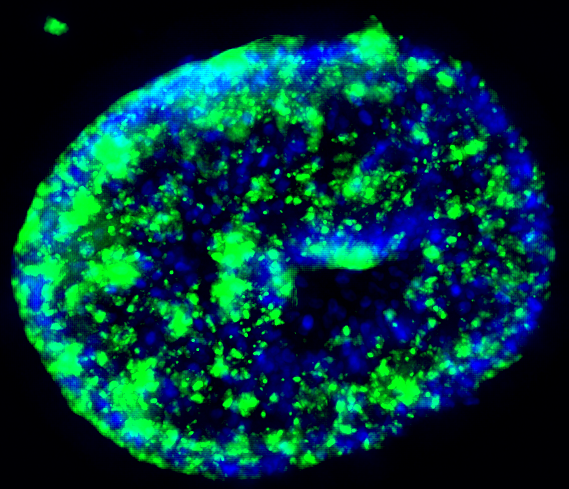

Supplement: Supplementary file 14 — Source Data for Figure 3 [file EMBJ-42-e113898-s007.zip › Figure_3/3H/oa-merge.tif]

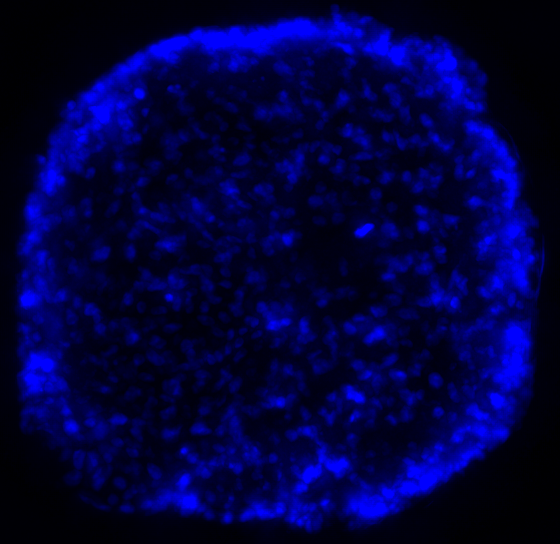

Supplement: Supplementary file 14 — Source Data for Figure 3 [file EMBJ-42-e113898-s007.zip › Figure_3/3H/pa-blue.tif]

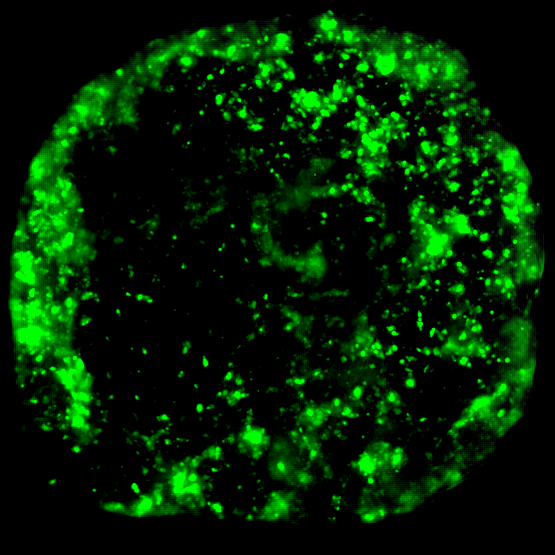

Supplement: Supplementary file 14 — Source Data for Figure 3 [file EMBJ-42-e113898-s007.zip › Figure_3/3H/pa-green.tif]

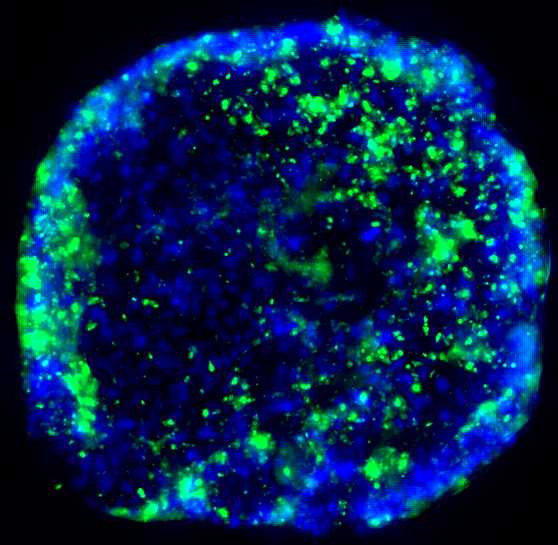

Supplement: Supplementary file 14 — Source Data for Figure 3 [file EMBJ-42-e113898-s007.zip › Figure_3/3H/pa-merge.tif]

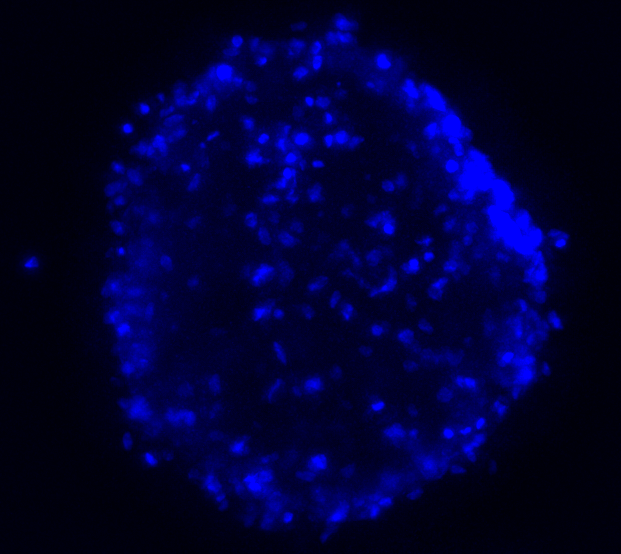

Supplement: Supplementary file 14 — Source Data for Figure 3 [file EMBJ-42-e113898-s007.zip › Figure_3/3H/TGF-blue.tif]

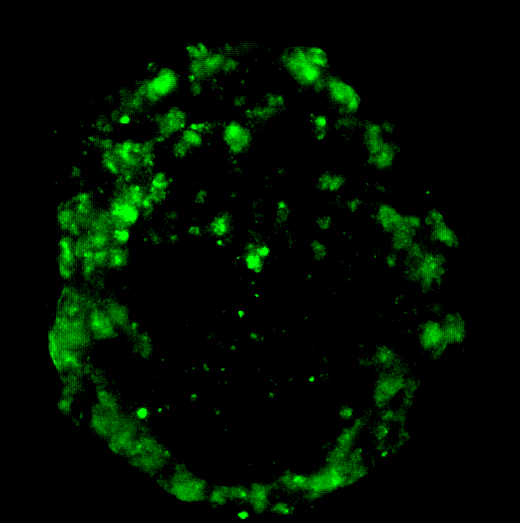

Supplement: Supplementary file 14 — Source Data for Figure 3 [file EMBJ-42-e113898-s007.zip › Figure_3/3H/TGF-green.tif]

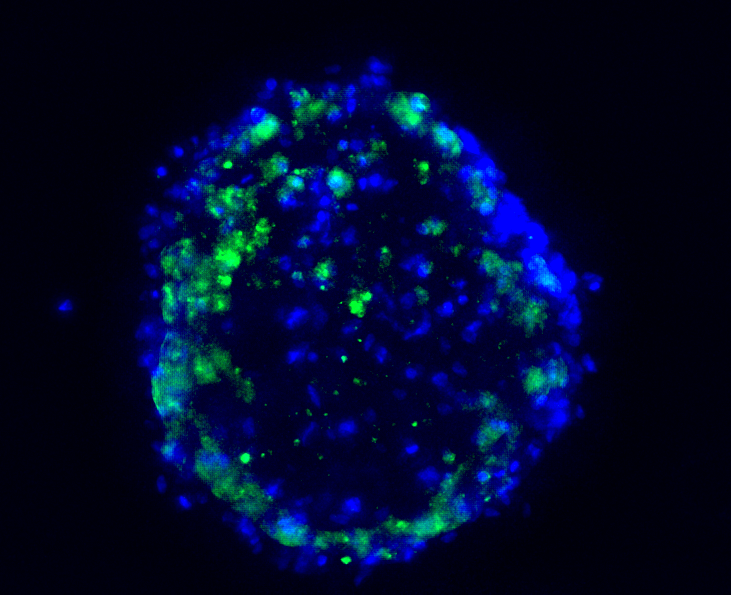

Supplement: Supplementary file 14 — Source Data for Figure 3 [file EMBJ-42-e113898-s007.zip › Figure_3/3H/TGF-merge.tif]
