# Supplementary material for: Single‐cell transcriptomics stratifies organoid models of metabolic dysfunction‐associated steatotic liver disease
Source: EMBO J. 2023 Nov 14;42(24):e113898. doi: 10.15252/embj.2023113898 (PMC10711666; doi:10.15252/embj.2023113898)
Supplement: Supplementary file 17 [file EMBJ-42-e113898-s016.pdf]

THE EMBO JOURNAL

Single-cell transcriptomics stratifies organoid models of metabolic dysfunction-associated steatotic liver disease

Anja Hess, Stefan Gentile, Amel Saad, Raza-Ur Rahman, Tim Habboub, Daniel Pratt, and Alan Mullen
DOI: 10.15252/embj.2023113898

Corresponding authors: Alan Mullen (Alan.Mullen@umassmed.edu) , Anja Hess (ahess@molgen.mpg.de)

Review Timeline:

Submission Date:	28th Feb 23
Editorial Decision:	7th Apr 23
Revision Received:	22nd Jul 23
Editorial Decision:	10th Sep 23
Revision Received:	4th Oct 23
Accepted:	6th Oct 23

Editor: Daniel Klimmeck

Transaction Report:

(Note: With the exception of the correction of typographical or spelling errors that could be a source of ambiguity, letters and reports are not edited. Depending on transfer agreements, referee reports obtained elsewhere may or may not be included in this compilation. Referee reports are anonymous unless the Referee chooses to sign their reports.)

Dear Dr Mullen,

Thank you again for the submission of your manuscript (EMBOJ-2023-113898) to The EMBO Journal. Please accept my sincere apologies for getting back to you with unusual protraction due to delayed referee input, as well as detailed discussion in the editorial team. Your study was assessed by three reviewers with expertise in liver / organoid biology (referees #1, #2) and single-cell analyses (referee #3), whose comments are enclosed below.

As you will see, the experts acknowledge the good quality and originality of the analysis and potential interest of your resource in vitro characterisation of treatment-induced NAFLD phenotypes at single-cell level. However, they also express major concerns, which need careful consideration before they can endorse publication of your study. In more detail, referee #1 states important issues regarding comprehensive characterisation of the FFA induced conditions (ref#1, pt.2,4) and requests additional experiments to increase robustness and generality of the results (ref#1, pts. 3,6). Reviewer #2 asks you to better integrate markers of liver zonation and related concepts into your characterisation (ref#2, pt1). Referee #3 requests clarification of cell type annotation, variability of the cultures as well as additional parameters of the analysis (ref#3, pts 1-5). In addition, the experts raise a number of issues related to methods annotation, data representation and discussion of related literature findings as well as overall structure of the manuscript that would need to be conclusively addressed to achieve the level of robustness and clarity needed for The EMBO Journal.

Given the overall interest stated, we are able to invite you to revise your manuscript experimentally to address the referees' comments. In light of the extensive experimentation requested, I would appreciate if you could contact me during the next weeks for exchange e.g. a video call to discuss your perspective on the comments and potential plan for revisions.

We generally allow three months as standard revision time. As a matter of policy, competing manuscripts published during this period will not negatively impact on our assessment of the conceptual advance presented by your study. However, we request that you contact the editor as soon as possible upon publication of any related work, to discuss how to proceed. Should you foresee a problem in meeting this three-month deadline, please let us know in advance and we may be able to grant an extension.

When submitting your revised manuscript, please carefully review the instructions below.

Please feel free to approach me any time should you have any questions related to this.

Thank you for the opportunity to consider your work for publication.

I look forward to your revision.

Kind regards,

Daniel Klimmeck

Daniel Klimmeck, PhD
Senior Editor
The EMBO Journal

Instruction for the preparation of your revised manuscript:

1) a .docx formatted version of the manuscript text (including legends for main figures, EV figures and tables). Please make sure that the changes are highlighted to be clearly visible.

2) individual production quality figure files as .eps, .tif, .jpg (one file per figure).

3) a .docx formatted letter INCLUDING the reviewers' reports and your detailed point-by-point response to their comments. As part of the EMBO Press transparent editorial process, the point-by-point response is part of the Review Process File (RPF), which will be published alongside your paper.

4) a complete author checklist, which you can download from our author guidelines (https://wol-prod-cdn.literatumonline.com/pb-assets/embo-site/Author Checklist%20-%20EMBO%20J-1561436015657.xlsx). Please insert information in the checklist that is also reflected in the manuscript. The completed author checklist will also be part of the RPF.

5) Please note that all corresponding authors are required to supply an ORCID ID for their name upon submission of a revised manuscript.

6) It is mandatory to include a 'Data Availability' section after the Materials and Methods. Before submitting your revision, primary datasets produced in this study need to be deposited in an appropriate public database, and the accession numbers and database listed under 'Data Availability'. Please remember to provide a reviewer password if the datasets are not yet public (see https://www.embopress.org/page/journal/14602075/authorguide#datadeposition).
In case you have no data that requires deposition in a public database, please state so in this section. Note that the Data Availability Section is restricted to new primary data that are part of this study.
*** Note - All links should resolve to a page where the data can be accessed. ***

7) Our journal encourages inclusion of *data citations in the reference list* to directly cite datasets that were re-used and obtained from public databases. Data citations in the article text are distinct from normal bibliographical citations and should directly link to the database records from which the data can be accessed. In the main text, data citations are formatted as follows: "Data ref: Smith et al, 2001" or "Data ref: NCBI Sequence Read Archive PRJNA342805, 2017". In the Reference list, data citations must be labeled with "[DATASET]". A data reference must provide the database name, accession number/identifiers and a resolvable link to the landing page from which the data can be accessed at the end of the reference. Further instructions are available at .

8) At EMBO Press we ask authors to provide source data for the main and EV figures. Our source data coordinator will contact you to discuss which figure panels we would need source data for and will also provide you with helpful tips on how to upload and organize the files.

Numerical data can be provided as individual .xls or .csv files (including a tab describing the data). For 'blots' or microscopy, uncropped images should be submitted (using a zip archive or a single pdf per main figure if multiple images need to be supplied for one panel). Additional information on source data and instruction on how to label the files are available at .

9) We replaced Supplementary Information with Expanded View (EV) Figures and Tables that are collapsible/expandable online (see examples in https://www.embopress.org/doi/10.15252/embj.201695874). A maximum of 5 EV Figures can be typeset. EV Figures should be cited as 'Figure EV1, Figure EV2" etc. in the text and their respective legends should be included in the main text after the legends of regular figures.

- For the figures that you do NOT wish to display as Expanded View figures, they should be bundled together with their legends in a single PDF file called *Appendix*, which should start with a short Table of Content. Appendix figures should be referred to in the main text as: "Appendix Figure S1, Appendix Figure S2" etc. See detailed instructions regarding expanded view here: .

- Additional Tables/Datasets should be labelled and referred to as Table EV1, Dataset EV1, etc. Legends have to be provided in a separate tab in case of .xls files. Alternatively, the legend can be supplied as a separate text file (README) and zipped together with the Table/Dataset file.

10) When assembling figures, please refer to our figure preparation guideline in order to ensure proper formatting and readability in print as well as on screen:
http://bit.ly/EMBOPressFigurePreparationGuideline

Please remember: Digital image enhancement is acceptable practice, as long as it accurately represents the original data and conforms to community standards. If a figure has been subjected to significant electronic manipulation, this must be noted in the figure legend or in the 'Materials and Methods' section. The editors reserve the right to request original versions of figures and the original images that were used to assemble the figure.

11) For data quantification: please specify the name of the statistical test used to generate error bars and P values, the number (n) of independent experiments (specify technical or biological replicates) underlying each data point and the test used to calculate p-values in each figure legend. The figure legends should contain a basic description of n, P and the test applied. Graphs must include a description of the bars and the error bars (s.d., s.e.m.).

Please remember: Digital image enhancement is acceptable practice, as long as it accurately represents the original data and conforms to community standards. If a figure has been subjected to significant electronic manipulation, this must be noted in the figure legend or in the 'Materials and Methods' section. The editors reserve the right to request original versions of figures and the original images that were used to assemble the figure.

Further information is available in our Guide For Authors: https://www.embopress.org/page/journal/14602075/authorguide

We realize that it is difficult to revise to a specific deadline. In the interest of protecting the conceptual advance provided by the work, we recommend a revision within 3 months (6th Jul 2023). Please discuss the revision progress ahead of this time with the editor if you require more time to complete the revisions. Use the link below to submit your revision:

https://emboj.msubmit.net/cgi-bin/main.plex

Referee #1:

Hess et al. derive PSC-based human liver organoids and perform different injury treatments conjugated to scRNA-sequencing analyses to evaluate how and which of these models best resemble NAFLD phenotypes. The authors find that treatment with TGFB1 induces most strongly an inflammatory phenotype linked to stellate cell expansion. In general, this study adds a significant amount of novel scRNA-seq data on the analysis and characterization of cells from the HLO model under different treatments. However, several major concerns should be addressed to be able to better judge these models in the context of faithful disease modelling of NAFLD.

Major:

1-The original publication of the HLO protocol (Ouchi et al., fig. 1F) reports a rather different distribution/annotation between hepatocyte-like and biliary-like cells as compared to in the current manuscript. What causes this difference in cholangiocyte abundancy, is it purely cell type annotation? Did the authors detect some Kupffer cells in the current HLO? The cholangiocyte population in vivo is much smaller as compared to the hepatocyte population, but in the HLOs this seems to be 50-50. Likewise, the contribution of stellate cells is majorly overrepresented in HLOs as what is found in vivo. The authors should carefully discuss these characteristics of the HLOs and put their data into context knowing these inherent system limitations of the systems, as it can majorly impact and influence the modelled disease phenotypes.

2-The paper is bioinformatically rich which the authors should be commended for. However, the intention is to model NAFLD, yet the analyses solely focus on later-stage fibrotic NASH phenotypes. The only panel focused on lipid phenotypes is presented in a very small image of H&E in Fig. 2h, which is not convincing. In NAFLD, NASH and fibrosis develop through steatosis. A thorough characterization, especially in the FFA conditions, of lipid accumulation (e.g. Nile Red) and other measures of fat accumulation (TAG content, etc.) should be evaluated in the HLOs and be combined with hepatocyte-specific stainings to show selective accumulation of lipids in the hepatocytes, as would expected. This is imperative to judge whether HLOs model faithful NASH or rather non-NAFLD related fibrosis. If not, the scope of the paper should be changed to fibrosis modelling. In general, to name a model that is just exposed by TGFB1 a NAFLD model is by default incorrect.

3-The batch effects between cell type composition are very large (Fig. 3c, e.g. regarding stellate cells) in the orbital shaker. ULA actually seems more stable in cell type composition (although, arguably analyzed at lower n). This makes me question a) whether the orbital shaker can indeed be considered the better method regarding stability, and 2) whether the observation about stellate cell expansion/reduction are not (at least partly) due to unreliable HLO preparation. The n is too low here: n = 2 OA, n = 2 PA, n = 2 TGFB1. The control organoids from Fig. 3c easily range between 5-50% stellate cell content. What is the difference between controls from C-TGF vs the controls of C-OA, where again the controls majorly differ? Is the same effect on stellate cell numbers seen when experiments are performed in the ULA? Finally, these are cell type distribution analyses. Is it absolute cell count that should increase/decrease or is there rather expansion of another cell population (e.g. ductular population) but absolute numbers of stellate cells stay the same. Finally, at the moment, sc-seq is only used to derive all these conclusions on stellate cell numbers by TGFB1, PA, and OA exposure. These claims should be substantiated by stainings with accompanying quantifications for the different cell types in the HLOs (over multiple batches).

4-The authors should provide rationale as to why the specific concentrations of OA and PA were used. The concentration used for PA is very high and not in vivo relevant. Was the viability of the HLOs impacted by these treatments? A dose response curve to evaluate different OA and PA concentrations (e.g. regarding steatosis/fibrosis) phenotypes should be performed to show dose dependency. In addition, why did the authors choose to expose to single FFAs?

5-The manuscript is rather jumpy between disease modelling and evaluating different culture conditions. Fig. 3 presents characterization of different culture conditions on HLO cell type composition. This seems more logical as a follow-up to Fig. 1. Rearranging the manuscript to first focus on the HLO characterization + testing of different culture conditions, and thereafter fibrosis modelling under the different conditions seems more logical.

6-Experiments are performed solely with the H1 ESC line. Some of the major claims and findings of this manuscript should be preferably repeated with another ESC/iPSC line to add robustness to the observations.

7-Images and other stainings of the HLOs are scant and only provided as very small panels of rather low resolution, this makes evaluating the claims based on these images are rather difficult. The size of the images (and resolution) should be improved and multiple examples of stainings can be provided in the supplement to show more robustness of the system. Related to this, do the authors experience batch effects with HLOs?

Minor:
-How is the size difference of HLOs accounted for? Does size impact disease phenotypes?

-Several typos in the manuscript should be corrected (e.g. ForceAtals2)

-What is the meaning of writings like "n = 1-3 experiments"? Please mention the precise n and whether this indicates batches (experiments) or organoids (technical replicates).

Referee #2:

Anja Hess and colleagues provide a very detailed assessment of different human liver organoid (HLO) models, ranging from different culture and treatment conditions to very elegant single cell RNA sequencing. Their findings are timely and important for scientists across different disciplines. The majority of drugs tested in clinical trials in the NASH space have failed despite promising preclinical data derived in mouse high fat diet models. It is therefore critical to advance complex human ex vivo models and this study provides an important toolbox manual and comparison, while also highlighting exciting concepts around the multicellular dynamics in NFALD. The work by Hess and colleagues does not only suggest why olive oil is healthier and others, it also provides explanations for some discrepancies involving different NAFLD pathologies. Overall, I find this manuscript very exciting with beautiful illustrations, rich data, stunning bioinformatics analyses and solid conclusions. There is little to criticize but few points could be addressed to further improve an already excellent manuscript.

1) Many important NASH targets are zonated and impaired lipid metabolism and derailed metabolic zonation is a hallmark of NAFLD and NASH (recently reviewed in PMID: 36693201). Two major NASH targets, PNPLA3 and HSD17B13, are primarily expressed in periportal hepatocytes and current in vitro models show poor periportal gene expression, thus preventing studying these targets in their physiological environment. The authors should extend the nice zonal marker analysis shown in Figure 3H to the other conditions/models as well. This may guide researcher to pick the best model for their needs.

2) I don't know whether it is possible to obtain a NASH drug that has failed in the clinic. But it would be very interesting to see how this performs in the HLO models and how the models could have predicted lack of efficacy.

3) Many Figure panels (e.g. Figs 1b, 3d, 4d, 5f, 6h) are very small and difficult to read unless zooming into each part closely. When printed it is difficult to see details. Also, the individual data points are barely visible in Fig1b due to the size and color choices. At the same time many Figures have large empty white spaces between the panels. Hopefully, some rearrangements allow increasing size to readable levels.

4) I was recently shown that more HNF4a+ bipotent progenitor cells appear in patients with cirrhosis when compared to those having the same noncirrhotic disease (PMID: 36914834). Could be added to the discussion section where the authors discuss the connection between TGFb and HNF4a+ bipotent progenitor cells. This paper could also be added to the results section where reference 25 is cited.

Referee #3:

Organoids are promising tools in biomedical research as they allow studying development, diseases and drug responses without the issues and complexities of animal experiments. However, organoids often exhibit great variability in cell type composition, and it is not clear how well disease models mimic patient data.
Here, the authors systematically compare models for non-alcoholic fatty liver disease (NAFLD) that they obtain by treatment of human liver organoids with oleic acid (OA), palmitic acid (PA) and TGFß1. They analyze the transcriptomes of ~100k, as well as selective phenotype data, and find that while all three models induce inflammatory signatures, only the treatment with TGFß1 induces collagen production, fibrosis and the expansion of hepatic stellate cells.
The authors furthermore compare different mechanical environments during the growth of the organoids and conclude that culturing of HLO on an orbital shaker best resembles the inflammatory and fibrotic response to TGFß1. Additionally, they compared the inflammatory and fibrotic response of the TGFß1 and fatty acid treatment on a transcriptome level, analyzed ligand-receptor interactions and the differentiation trajectories of hepatic and stellate lineages. Finally, the authors used a set of

marker genes to calculate the NAFLD severity score per condition and found that these progress from OA to PA to TGFß1.

While liver biology is not our area of expertise, we found this work very interesting and overall well-executed. The manuscript is well-written and discusses interesting and important points that need to be considered when establishing organoids as a disease model for NAFLD.
However, we think that there are some concerns that might affect the validity of the authors' conclusions and, therefore, should be addressed.

Major Comments

1. Cell type annotation. The authors use three different methods to perform cell type annotation for the first scRNA-seq dataset they present in Figure 1d. Confirming cell identity is very important in this study, and it is certainly a positive aspect that the authors wanted to verify the robustness of the results with alternative methods. Also, adding "-like" to organoid cell type names is good to emphasize that the transcriptome of organoid cells is generally different from cells found in vivo. However, there are aspects that should be improved or clarified:
a. First, isn't there a large overlap between the markers used in Method 1 and Method 2? If anything, it sounds like Method 2 might be better as it uses a set of validated markers available from a database. Thus, it is unclear what is the benefit of using Method 1.
b. Figure 1d shows that the overlap between the annotations obtained with Method 3 and those obtained with the first two methods is quite bad. For example, apart from the absence of the Embryonic stem cells (which seem to have been allocated to a mix of other cell types), what was "Hepatic Stem Cells" is annotated as "Fibroblast" in method 3, and there is a large variability of labels in both the Hepatocytes and the Bi-Potent clusters. This result is particularly worrying as Method 3 is the most unbiased approach (given that it's not based on a pre-selected list of markers) among those that the authors used and is the only one that works at the single-cell level (rather than at a cluster level). These mismatches might indicate that the reference dataset the authors are using is not appropriate. Or, this might be an indication that the cell-type (transcriptional) identity in the organoid is not compatible with the one found in vivo.
c. The claims about the absence of rare cell types (i.e., the embryonic stem-cell like cluster, see Figure 3b,c) should be verified with algorithms that are specifically designed to detect rare cells, given that standard clustering algorithms can be very inefficient in detecting them.
d. On page 28 in the Methods section, the authors write that when using markers for cell type annotation they perform an enrichment test and assign identity even when the statistical significance criterion they choose is not met. This is dangerous and might lead to wrong cell-type annotations. At the very least, the authors should report: which cluster annotations do not reach statistical significance, what is the most likely annotation (based on the lowest but non-significant p-value), what are the marker genes that are missing, and add clear caveats in the text when they talk about such clusters.
e. Finally, the clustering parameters were fixed in such a way to obtain a number of clusters equal to the expected number of cell types. However, an unbiased criterion (e.g., based on cluster robustness) is much more desirable here, given that the difference in cell type identity and composition across conditions is one of the main points made in the paper.

2. Cell abundance testing. Multiple claims are made about differences in cell abundance between conditions, or along trajectories. The authors should perform statistical tests to verify these claims and provide a measure of statistical significance (taking into account the number of cells, replicates, and giving statistical uncertainty). There are several packages available to do that (e.g., https://www.nature.com/articles/s41467-021-27150-6).

3. Organoid variability. Their analysis shows that there's some inter-organoid variability, as seen, for example, from the cell-type composition of OS organoids shown in Figure 3c. We think there are two important points to consider:
a. it has been observed that organoid variability can affect conclusions on organoid-based perturbation studies (https://www.biorxiv.org/content/10.1101/2022.09.27.509783v1). The authors should discuss if and how organoid variability can affect their claims, especially in cases with fewer replicates (e.g., in Figure 4 where they have N=2 replicates per condition).
b. In the methods section, they say that "Clusters were required to represent all individual replicates". First, it is unclear what this means: what do they do if the algorithm identifies a cluster that does not include cells from all replicates? is it merged with others? If so, how? Second, this approach might lead to an underestimation of inter-organoid variability. The authors should perform clustering without imposing such constraint so that a more unbiased estimation of organoid variability is obtained.

4. Organoid day. Organoids were grown until day 21 in all conditions. Could some of the differences they observed across conditions be due to the fact that the development of the organoid becomes slower/faster in different conditions? And would this impact their conclusions?

5. Communication analysis. The authors show that there are differences in cell communication across different conditions. This is interesting, but they should perform a statistical analysis of these results and list the communication patterns that are statistically significant.

6. Data Imputation. They perform data imputation for the analysis in Figure 6g with MAGIC. Using data-smoothing-based methods (like MAGIC) has been associated with an increase in false positives (https://www.ncbi.nlm.nih.gov/pmc/articles/PMC6415334/). Is data-imputation necessary for this analysis? Can it be avoided?

7. Methods. There are a few points in the Methods section that need clarification or additions.
a. Why putting a max (rather than min) threshold on the number of counts (30k)? The usual approach is to impose a threshold on the minimum number of counts.
b. how do they select highly variable genes?
c. what do they mean by scaling the expression values to a max value of 5? Is it a trimming (i.e., whatever is above 5 gets assigned a value of 5)?
d. The number (and fraction) of cells removed after QC in each dataset should be reported.

8. The authors highlight that "integrating data from each model with that of NAFLD patients across disease progression further demonstrates PA and TGF-β1 more robustly model inflammation and fibrosis" (page 2).
We think that this claim is misleading. What the authors do instead is use a scoring system that is based on 26 and 98 marker genes, respectively, to assess and score the severity of the different NAFLD models. A way to keep the claim of an integration approach would be to actually integrate scRNAseq data from healthy or diseased donors, and compare composition of different cell types. Indeed, if such data were available, this would be very interesting for the reader and a good possibility to directly evaluate how well disease models represent patient data

9. In the data availability statement, the authors write: "The following custom scripts are available upon request and will be made publicly available upon release.". What are the "following custom scripts" that will be made publicly available? Did the authors intend to list the scripts? Moreover, the code should have been made available to Reviewers before publication so that reproducibility could be verified.

Minor comments
- Figure 1:
- Adding axes would greatly improve readability, e.g. Fig. 1b,f, also 2 i-k
- f: Unify legends for all three plots (odds ratio, p-value). Color of dots is not
always as shown in the legend - is this a continuous scale? Please change accordingly.

- Figure 2:
- Size of in-situ images should be increased
- Tilt 2g by 90{degree sign} to make axes the same as in 2 f to improve readability

- Figure 3:
- What do the authors mean by "broader distribution of HSC marker genes in ULA-HLOs" ?
- d: To compare marker gene expression for different conditions, rather use violin plots for cell types and conditions.
- h: Does the portion of zones in the organoid go in accordance with in vivo data?
- How are the conditions OS and ULA transcriptionally different that could explain their observed phenotypes in culture? What is the conclusion of the authors regarding the similarity of these models to NAFLD in patients?

- Figure 4:
- d: Controls show induction of connective tissue response to inflammation as well as fibroblast migration. How do the authors explain that?
- f: Readability can be improved. It's not clear which bars belong to which labels. ---
- Also, it would be interesting to see which of the terms come up in all conditions (overlap), and which are specific to one disease model.

- Figure 5:
Instead of a venn diagram, the use of an upset plot to visualize the overlap between ligand-receptor interactions might improve clarity

- Figure 7:
The way the authors present this analysis could summarize and emphasize the effect of the different models better. How about summarizing the scores in a dotplot or heatmap with cell types and models as axes instead of multiple violin plots?

Response Letter

Date: 2023-JUL-22

Referee #1: Hess et al. derive PSC-based human liver organoids and perform different injury treatments conjugated to scRNA-sequencing analyses to evaluate how and which of these models best resemble NAFLD phenotypes. The authors find that treatment with TGFB1 induces most strongly an inflammatory phenotype linked to stellate cell expansion. In general, this study adds a significant amount of novel scRNA-seq data on the analysis and characterization of cells from the HLO model under different treatments. However, several major concerns should be addressed to be able to better judge these models in the context of faithful disease modelling of NAFLD.

Major:
1.-The original publication of the HLO protocol (Ouchi et al., fig. 1F) reports a rather different distribution/annotation between hepatocyte-like and biliary-like cells as compared to in the current manuscript. What causes this difference in cholangiocyte abundancy, is it purely cell type annotation? Did the authors detect some Kupffer cells in the current HLO? The cholangiocyte population in vivo is much smaller as compared to the hepatocyte population, but in the HLOs this seems to be 50-50. Likewise, the contribution of stellate cells is majorly overrepresented in HLOs as what is found in vivo. The authors should carefully discuss these characteristics of the HLOs and put their data into context knowing these inherent system limitations of the systems, as it can majorly impact and influence the modelled disease phenotypes.

1.1 What causes this difference in cholangiocyte abundancy, is it purely cell type annotation?

Our study describes cell type distributions in HLOs that are different from the original study (Ouchi et al., 2019). This difference is due to evolving annotation strategies in the analysis of Ouchi et al.'s publicly available data (GSE130073), which reproduced large proportions of *KRT19/KRT7/CLDN4/EPCAM* positive cells (figure below, recapitulating the relatively high cholangiocyte fractions). Our study mainly utilizes the ScType database in which these genes are associated with cholangiocytes (Supplementary Table 1: Marker genes), leading to higher fraction of cholangiocyte-like cells detected by our method. We also compared three annotation strategies with similar results (Fig. 1d, Supplementary Fig. 2c-d). In addition, we find that continued culture in an orbital shaker results in an expansion of hepatocyte-like cells and reduction of cholangiocyte-like cells (Fig. 2b and c). We have now pointed out these limitations in the discussion more clearly.

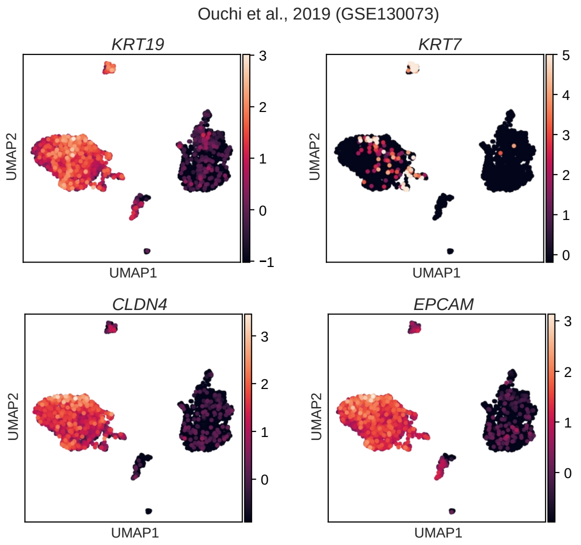

Fig. R1: Cholangiocyte marker gene expression and re-annotation of scRNA-seq data GSE130073.

1.2 Did the authors detect some Kupffer cells in the current HLO?

We do not confidently reproduce the presence of Kupffer cells in the HLO system. First, our RT-qPCR did not show the induction of *CD14* and *CLEC4F* when comparing hESCs and HLOs (not shown). Secondly, we only find inconsistent expression of *TLR4*, and *GPBAR1*, the other markers used by Ouchi et al. in our scRNA-seq data. Lastly, our database annotation strategy did not generate a hit for Kupffer cells. While we currently cannot exclude the presence of a small number of Kupffer cell-like cells, we are not able to identify a clear population. We have now pointed out these limitations in the discussion more clearly.

1.3 The cholangiocyte population in vivo is much smaller as compared to the hepatocyte population, but in the HLOs this seems to be 50-50.

We agree that the cholangiocyte-like populations in ULA-HLOs (Fig. 1d) are high in relative comparison to hepatocyte-like cells. We were partly able to address this limitation with the OS-method where cholangiocyte-like cells ranged from 4-12% in control conditions (Fig. 2b and c). We agree that this fraction however is higher than *in vivo* and added a discussion paragraph to point out these limitations.

1.4 Likewise, the contribution of stellate cells is majorly overrepresented in HLOs as what is found in vivo.

We agree and rewrote the discussion to point out these limitations in greater detail. One of our strategies to address this limitation was to optimize the HLO culture system, and we achieved an overall reduction of HSCs in orbital shaker-cultured HLOs (Fig. 2b and c).

2-The paper is bioinformatically rich which the authors should be commended for. However, the intention is to model NAFLD, yet the analyses solely focus on later-stage fibrotic NASH phenotypes. The only panel focused on lipid phenotypes is presented in a very small image of H&E in Fig. 2h, which is not convincing. In NAFLD, NASH and fibrosis develop through steatosis. A thorough characterization, especially in the FFA conditions, of lipid accumulation (e.g. Nile Red) and other measures of fat accumulation (TAG content, etc.) should be evaluated in the HLOs and be combined with hepatocyte-specific stainings to show selective accumulation of lipids in the hepatocytes, as would expected. This is imperative to judge whether HLOs model faithful NASH or rather non-NAFLD related fibrosis. If not, the scope of the paper should be changed to fibrosis modeling. In general, to name a model that is just exposed by TGFB1 a NAFLD model is by default incorrect.

We appreciate these suggestions and agree that the confirmation of lipid accumulation in the FFA conditions is important. We have now performed immunofluorescence BODIPY staining (fluorescent dye of neutral lipids similar to Nile Red) to measure lipid accumulation of orbital shaker-cultured HLOs exposed to OA, PA, and TGF-β1. This was performed in the original WA01 PSCs (**Fig. 3h**). We also performed this analysis in HLOs differentiated from a new iPSC line (**Supplementary Fig. 3c**).

In addition to BODIPY analysis, we also quantified triacylglycerol (TAG) / triglycerides as suggested (**Fig. 3j** and **Supplementary Fig. 3e**). For both PSC lines, we observed the most pronounced induction of lipid accumulation with OA treatment.

We attempted to add hepatocyte-specific staining to this analysis as recommended. We tried antibodies against surface and intracellular proteins, but the process of fixing the full organoids appeared to blunt the BODIPY signaling, and we were not able to obtain images of organoids co-stained with BODIPY and IF.

3-The batch effects between cell type composition are very large (Fig. 3c, e.g. regarding stellate cells) in the orbital shaker. ULA actually seems more stable in cell type composition (although, arguably analyzed at lower n). This makes me question a) whether the orbital shaker can indeed be considered the better method regarding stability, and 2) whether the observation about stellate cell expansion/reduction are not (at least partly) due to unreliable HLO preparation. The n is too low here: n = 2 OA, n = 2 PA, n = 2 TGFB1. The control organoids from Fig. 3c easily range between 5-50% stellate cell content. What is the difference between controls from C-TGF vs the controls of C-OA, where again the controls majorly differ? Is the same effect on stellate cell numbers seen when experiments are performed in the ULA? Finally, these are cell type distribution analyses. Is it absolute cell count that should increase/decrease or is there rather expansion of another cell population (e.g. ductular population) but absolute numbers of stellate cells stay the same. Finally, at the moment, sc-seq is only used to derive all these conclusions on stellate cell numbers by TGFB1, PA, and OA exposure. These claims should be substantiated by stainings with accompanying quantifications for the different cell types in the HLOs (over multiple batches).

We appreciate these comments and recommendations. As the reviewer points out, there is variability between each batch of HLO differentiation, which is why control differentiations are important for each analysis. Since our analysis was focused on orbital shaker conditions, we focused on validating the question of stellate cell numbers in this system over multiple rounds of HLO differentiation in the original PSC line. We also added a new iPSC line to validate these findings. HSCs are defined by expression of Desmin (*DES*) (Fig. 4c), and we quantified the expression of *DES* in control HLOs and those treated with TGF-β1, PA and OA in four separate experiments for each line (**Supplementary Fig. 4g-h**). There was a consistent and dramatic reduction of DES expression with OA treatment consistent with depletion of HSCs. TGF-β1 treatment showed a trend towards increased *DES* expression, but only achieved statistical significance in one line.

4-The authors should provide rationale as to why the specific concentrations of OA and PA were used. The concentration used for PA is very high and not in vivo relevant. Was the viability of the HLOs impacted by these treatments? A dose response curve to evaluate different OA and PA concentrations (e.g. regarding steatosis/fibrosis) phenotypes should be performed to show dose dependency. In addition, why did the authors choose to expose to single FFAs?

We appreciate the opportunity to clarify factors that went into selecting OA and PA concentrations. Ouchi et al. show significant accumulation of fat droplets through quantification of BODIPY 493/503 fluorescent intensity from 400 µM OA onward (Ouchi et al., 2019, https://doi.org/10.1016/j.cmet.2019.05.007, Fig. 2 and S3). We reasoned that a dose-response titration to induce steatosis in HLOs performed by these authors together with the recommendations in their follow-up paper (Thompson and Takebe, 2020,

https://doi.org/10.1016/bs.mcb.2020.03.009) would provide a reasonable basis to initiate experiments with a 500 µM OA dose. Based on the literature, we considered that “Plasma concentrations [...] ranged from 0.3 to 4.1 mmol/L for palmitic acid” (Abdelmagid et al., 2015, 10.1371/journal.pone.0116195) and reasoned that starting at the lower end of this range would be suitable to simulate PA concentrations relevant in vivo. Another study measures total 16:0 FA levels in serum from human subjects in the range from 500 to 3000 µM (Gallego et al., 2019, 10.3390/biom9010007, Fig. 4-5). We have also added a sentence describing the choice of concentrations into the Methods (sub-section Liver injury induction).

5-The manuscript is rather jumpy between disease modelling and evaluating different culture conditions. Fig. 3 presents characterization of different culture conditions on HLO cell type composition. This seems more logical as a follow-up to Fig. 1. Rearranging the manuscript to first focus on the HLO characterization + testing of different culture conditions, and thereafter fibrosis modelling under the different conditions seems more logical.

We thank the reviewer for this suggestion and have restructured the manuscript as suggested.

6-Experiments are performed solely with the H1 ESC line. Some of the major claims and findings of this manuscript should be preferably repeated with another ESC/iPSC line to add robustness to the observations.

We appreciate this suggestion and have now added analysis of a second iPSC line to support key findings in **Supplementary Fig. 3c, 3e, Supplementary Fig. 4g-h**.

7-Images and other stainings of the HLOs are scant and only provided as very small panels of rather low resolution, this makes evaluating the claims based on these images are rather difficult. The size of the images (and resolution) should be improved and multiple examples of stainings can be provided in the supplement to show more robustness of the system. Related to this, do the authors experience batch effects with HLOs?

We thank the reviewer for their comment and have uploaded an uncompressed version of our manuscript with high-resolution images. Multiple examples of high resolution stainings are now included in main **Fig. 3h, Supplementary Fig. 1**, additional images of BODIPY staining are included in **Supplementary Fig. 3c** and we have increased the size of other microscopy images. We also attach data from an additional replicate experiment (**Fig. R2**, not shown in Supplements due to space limitations).

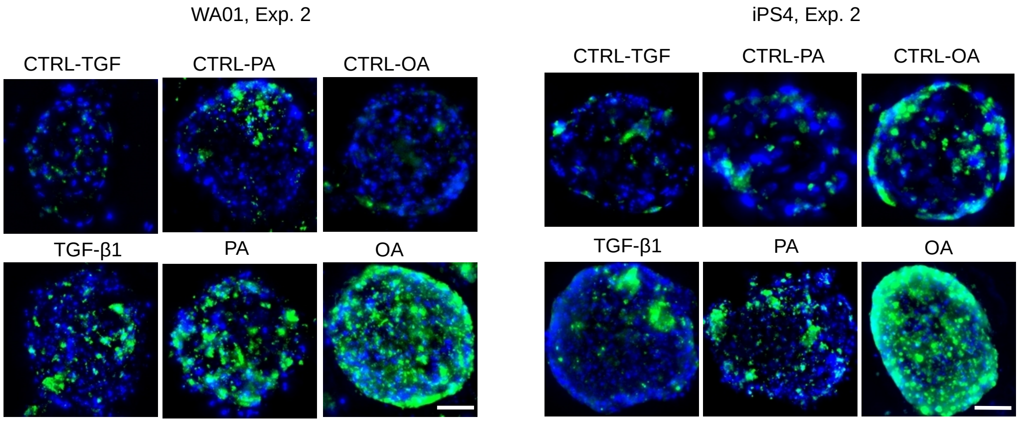

Fig. R2: Additional Hoechst (blue) and BODIPY (green) stainings. Scale bars, 50 µm.

Minor:

-How is the size difference of HLOs accounted for? Does size impact disease phenotypes?

This is an interesting question, the impact on disease phenotype has not been studied in detail. We have added a discussion part referencing our initial observations of size shifts in Matrigel-cultured HLOs and pointing out the potential to associate such changes with transcriptomic phenotypes in future studies.

-Several typos in the manuscript should be corrected (e.g. ForceAtals2)

We thank the reviewer and have carefully reviewed the manuscript to correct typos.

-What is the meaning of writings like "n = 1-3 experiments"? Please mention the precise n and whether this indicates batches (experiments) or organoids (technical replicates).

We thank the reviewer for their comment, “experiments” refer to individual experiments where PSCs are differentiated into organoids. We have specified this now in both the Methods section (Statistics and Reproducibility) and our figure legends, using *n* only for individual experiments and not for single organoids analyzed to avoid confusion.

Referee #2:

Anja Hess and colleagues provide a very detailed assessment of different human liver organoid (HLO) models, ranging from different culture and treatment conditions to very elegant single cell RNA sequencing. Their findings are timely and important for scientists across different disciplines. The majority of drugs tested in clinical trials in the NASH space have failed despite promising preclinical data derived in mouse high fat diet models. It is therefore critical to advance complex human ex vivo models and this study provides an important toolbox manual and comparison, while also highlighting exciting concepts around the multicellular dynamics in NFALD. The work by Hess and colleagues does not only suggest why olive oil is healthier and others, it also provides explanations for some discrepancies involving different NAFLD pathologies. Overall, I find this manuscript very exciting with beautiful illustrations, rich data, stunning bioinformatics analyses and solid conclusions. There is little to criticize but few points could be addressed to further improve an already excellent manuscript.

1) Many important NASH targets are zonated and impaired lipid metabolism and derailed metabolic zonation is a hallmark of NAFLD and NASH (recently reviewed in PMID: 36693201). Two major NASH targets, PNPLA3 and HSD17B13, are primarily expressed in periportal hepatocytes and current in vitro models show poor periportal gene expression, thus preventing studying these targets in their physiological environment. The authors should extend the nice zonal marker analysis shown in Figure 3H to the other conditions/models as well. This may guide researcher to pick the best model for their needs.

We appreciate this suggestion and incorporated the recommended analysis (**Supplementary Fig. 4f**). We find a relative loss of pericentral (score 5) hepatocyte-like cells with TGF-β1. OA and PA treatments are accompanied by a relative expansion of periportal (score 2) hepatocyte-like cells.

2) I don't know whether it is possible to obtain a NASH drug that has failed in the clinic. But it would be very interesting to see how this performs in the HLO models and how the models could have predicted lack of efficacy.

We find this suggestion very interesting and agree that such drug candidates would be suited to test the predictive capacity of NAFLD/NASH organoid models. Indeed, the farnesoid X receptor (FXR) agonist Ocaliva (obeticholic acid) has failed to meet the desired NASH endpoint while improving fibrosis (Younossi et al., 2019, https://doi.org/10.1016/S0140-6736(19)33041-7) and could serve as a drug to evaluate the power of HLO systems to show these differential effects. While the detailed study of such candidates in our opinion is beyond the scope of our paper, which mainly focuses on the systematic establishment and evaluation of NAFLD/NASH model systems with regard to the three major cell types of the liver, we added this as an outlook to the discussion section.

3) Many Figure panels (e.g. Figs 1b, 3d, 4d, 5f, 6h) are very small and difficult to read unless zooming into each part closely. When printed it is difficult to see details. Also, the individual data points are barely visible in Fig1b due to the size and color choices. At the same time many Figures have large empty white spaces between the panels. Hopefully, some rearrangements allow increasing size to readable levels.

We thank the reviewer for their suggestion and have enlarged figures 1b and others whenever possible. We have also worked to reduce white space between panels where possible.

4) I was recently shown that more HNF4a+ bipotent progenitor cells appear in patients with cirrhosis when compared to those having the same noncirrhotic disease (PMID: 36914834). Could be added to the discussion section where the authors discuss the connection between TGFb and HNF4a+ bipotent progenitor cells. This paper could also be added to the results section where reference 25 is cited.

We thank the reviewer for their interesting suggestion and have included the reference to the discussion and results sections.

Referee #3: Organoids are promising tools in biomedical research as they allow studying development, diseases and drug responses without the issues and complexities of animal experiments. However, organoids often exhibit great variability in cell type composition, and it is not clear how well disease models mimic patient data.

Here, the authors systematically compare models for non-alcoholic fatty liver disease (NAFLD) that they obtain by treatment of human liver organoids with oleic acid (OA), palmitic acid (PA) and TGFß1. They analyze the transcriptomes of ~100k, as well as selective phenotype data, and find that while all three models induce inflammatory signatures, only the treatment with TGFß1 induces collagen production, fibrosis and the expansion of hepatic stellate cells.
The authors furthermore compare different mechanical environments during the growth of the organoids and conclude that culturing of HLO on an orbital shaker best resembles the inflammatory and fibrotic response to TGFß1. Additionally, they compared the inflammatory and fibrotic response of the TGFß1 and fatty acid treatment on a transcriptome level, analyzed ligand-receptor interactions and the differentiation trajectories of hepatic and stellate lineages. Finally, the authors used a set of marker genes to calculate the NAFLD severity score per condition and found that these progress from OA to PA to TGFß1.

While liver biology is not our area of expertise, we found this work very interesting and overall well-executed. The manuscript is well-written and discusses interesting and important points that need to be considered when establishing organoids as a disease model for NAFLD.
However, we think that there are some concerns that might affect the validity of the authors' conclusions and, therefore, should be addressed.

Major Comments

1. Cell type annotation. The authors use three different methods to perform cell type annotation for the first scRNA-seq dataset they present in Figure 1d. Confirming cell identity is very important in this study, and it is certainly a positive aspect that the authors wanted to verify the robustness of the results with alternative methods. Also, adding "-like" to organoid cell type names is good to emphasize that the transcriptome of organoid cells is generally different from cells found in vivo. However, there are aspects that should be improved or clarified:

a. First, isn't there a large overlap between the markers used in Method 1 and Method 2? If anything, it sounds like Method 2 might be better as it uses a set of validated markers available from a database. Thus, it is unclear what is the benefit of using Method 1.

We agree that there is a significant overlap between the literature-derived marker genes in Method 1 and the database-derived ones in Method 2. Our intent was to show different approaches commonly applied to labeling clusters in single cell data to understand how these might affect

annotation and to explain why we chose one approach for further analysis. We have therefore now de-emphasized method 1 in the main **Fig. 1** and text and moved to the supplement.

To clarify the initial use of method 1 please find our two rationales below:

1. Most databases are largely based on adult tissue. In the case of organoids that are derived from pluripotent stem cells we were reasoning that the occurrence of specific liver progenitor cell types would not be covered by such standard databases. Thus, Method 1 allowed us to manually include progenitor subtypes into our annotation strategy, such as the hepato-pancreatic progenitor and the biliary tree stem cell. Although our clusters were not attributed to these subtypes, this was not predictable in the beginning.
2. Our strategy at that time was based on the recommendations by Clarke et al. (*Nat Protocols*, 2021) recommending the combination of i) automated and ii) manual annotation strategies (Fig. 2: Cell annotation workflow). Accordingly, database- (Method 2) and SingleCellNet (Method 3 in the original manuscript) fell into the automated strategies, while we attributed the selection of a set of marker genes by our group as manual annotation (Method 1). Therefore, we aimed at integrating strategies from both categories.

b. Figure 1d shows that the overlap between the annotations obtained with Method 3 and those obtained with the first two methods is quite bad. For example, apart from the absence of the Embryonic stem cells (which seem to have been allocated to a mix of other cell types), what was "Hepatic Stem Cells" is annotated as "Fibroblast" in method 3, and there is a large variability of labels in both the Hepatocytes and the Bi-Potent clusters. This result is particularly worrying as Method 3 is the most unbiased approach (given that it's not based on a pre-selected list of markers) among those that the authors used and is the only one that works at the single-cell level (rather than at a cluster level). These mismatches might indicate that the reference dataset the authors are using is not appropriate. Or, this might be an indication that the cell-type (transcriptional) identity in the organoid is not compatible with the one found in vivo.

We appreciate the reviewer's comment. The Sharma et al. 2020 (Sharma et al., 2020, 10.1016/j.cell.2010.02.027) dataset we originally used relied largely on adjacent normal liver tissue from liver cancer patients. A more recent reference os available that captures the developing human liver (Wesley et al., 2022, 10.1038/s41556-022-00989-7) that we think biologically is more suitable to annotate organoids that are derived from pluripotent stem cells. We now also use a logistic regression classifier-based method, CellTypist (Domínguez Conde et al., 2022, 10.1126/science.abl5197), for annotation and have included the results in our **Supplementary Figure 2d**. We find a better general overlap consistent with the presence of cholangiocyte-like cells, stellate cell-like cells and hepatoblast-like cells (hepatocyte precursors) in the ULA-HLOs. We find embryonic stem cell-like cells annotated as hepatoblast-like cells, however, embryonic

stem cells are not present in the reference dataset of developing human liver. We have expanded the discussion now more clearly pointing out the premature features of the HLO cell types as a potential limitation of our study.

c. The claims about the absence of rare cell types (i.e., the embryonic stem-cell like cluster, see Figure 3b,c) should be verified with algorithms that are specifically designed to detect rare cells, given that standard clustering algorithms can be very inefficient in detecting them.

We agree that a verification of the observed absence of embryonic stem cell (ESC)-like cells in OS-cultured HLOs is important and find the option to test for rare cell types exciting. Therefore, we analyzed this dataset with **CIARA** (Lubatti et al., 2022, 10.1101/2022.08.01.501965). We do not find highly localized transcripts voting for stem cell identity in our OS-HLOs (**Fig. R4**). We queried the top 30 genes identified by CIARA with CellMarker Augmented 2021 and did not detect enrichment indicating embryonic-stem cell-like clusters in OS-HLOs. Additionally, we do not detect canonical embryonic stem cell marker genes *NANOG, POU5F1, UTF1* in OS-HLOs, while they are present in ULA-HLOs (**Fig. R5**).

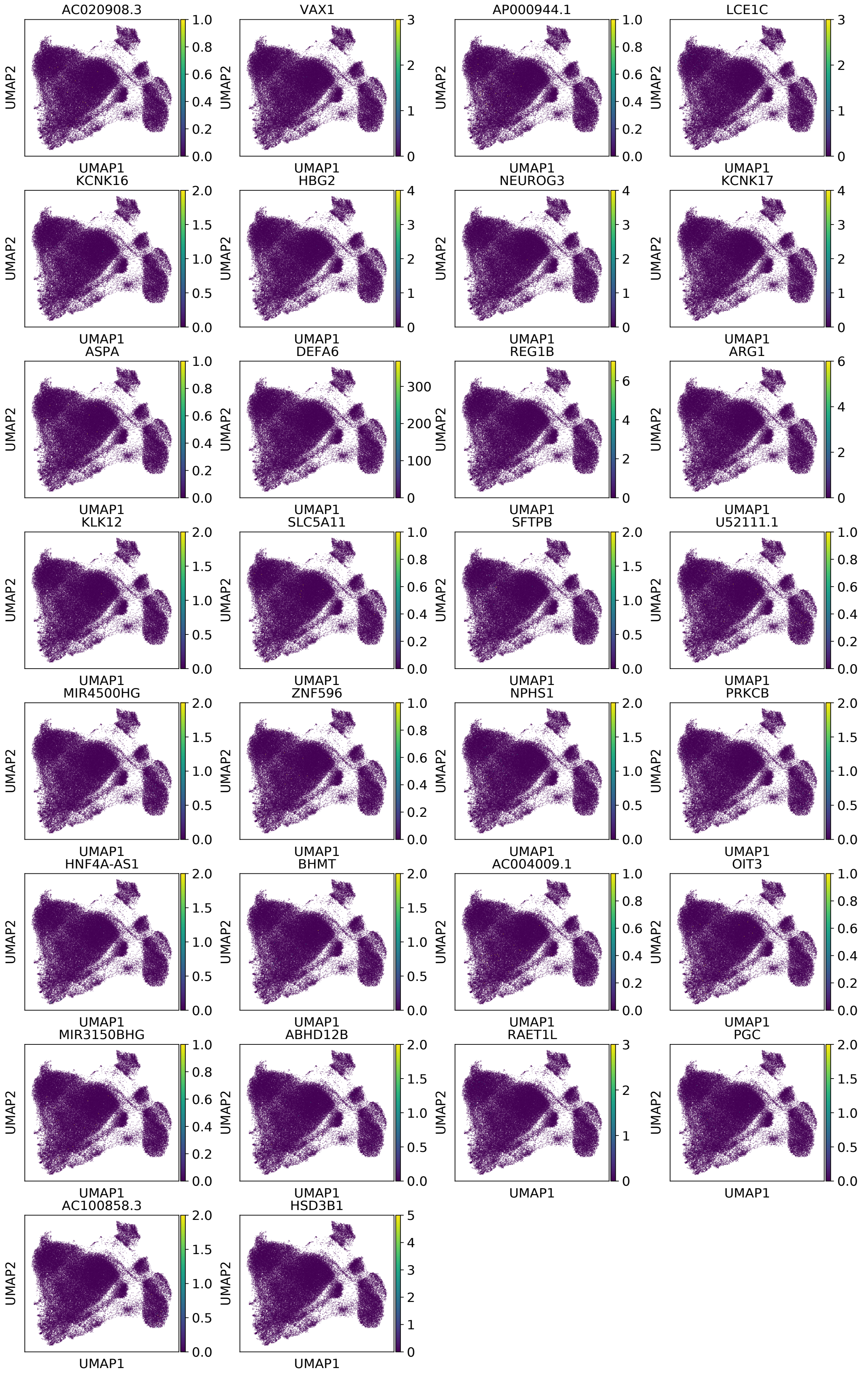

Fig. R4: Top 30 CIARA outputs projected on the UMAP representation of OS-HLOs.

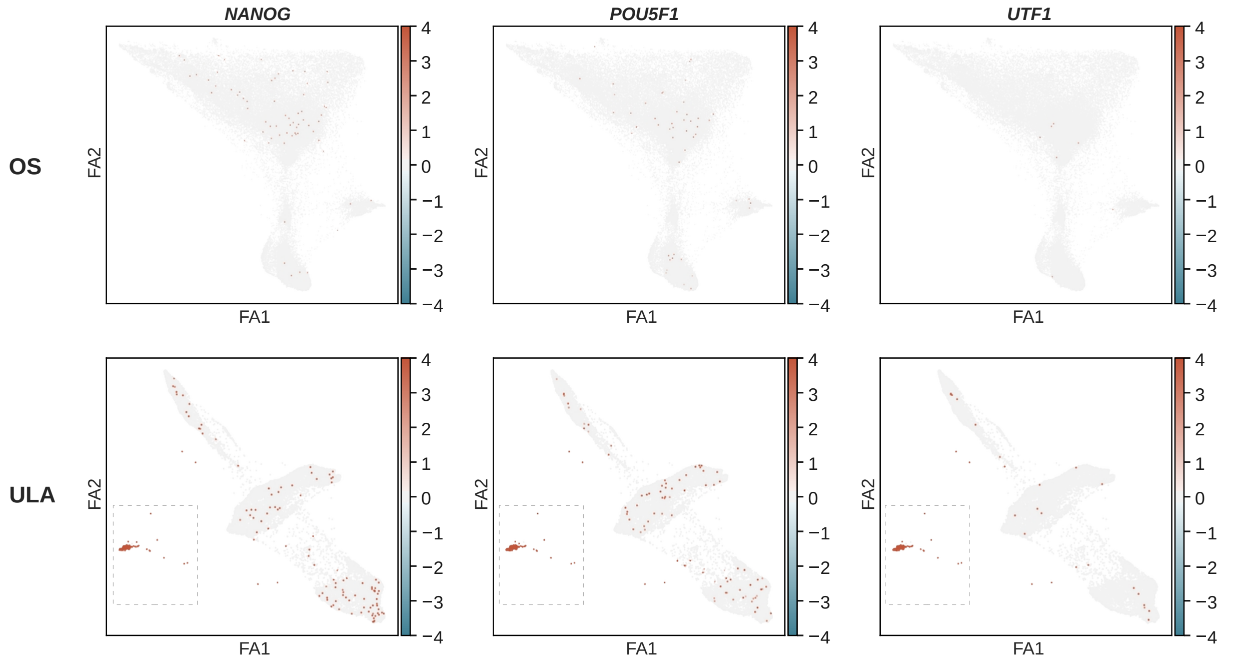

Fig. R5: Mean normalized expression of embryonic stem cell marker genes in OS- and ULA-HLOs.

d. On page 28 in the Methods section, the authors write that when using markers for cell type annotation they perform an enrichment test and assign identity even when the statistical significance criterion they choose is not met. This is dangerous and might lead to wrong cell-type annotations. At the very least, the authors should report: which cluster annotations do not reach statistical significance, what is the most likely annotation (based on the lowest but non-significant p-value), what are the marker genes that are missing, and add clear caveats in the text when they talk about such clusters.

We have now added a full supplementary table (**Supplementary_Table_2_Database-Annotations.ods, Fig. R6**) providing ranked annotations with *p*-values for each dataset. We additionally integrated *p*-values in all main figure legends, and main figure labels whenever possible with regard to space limitations. Additionally, we have changed the text in order to clearly highlight when clusters were assigned based on the most likely annotation. We also utilize a thorough nomenclature indicating cell types as “-like” throughout our manuscript in order to raise awareness for the fact that organoid cell types at their current stage are mimicking but not entirely resembling their vivo counterparts.

Hepatocyte-like					
**Term**	**Term**	**Overlap**	**P-value**	**Adjusted P-value**	**Genes**
**Hepatocytes**	**Hepatocytes**	**18/134**	**4.87999828318687E-06**	**0.000356239874672642**	**BNIP3;AFP;A1CF;TM4SF4;APOA1;FOXA1;GLUL;CDH1;KRT18;KRT8;FGB;SPTBN1;TTR;APOA2;APOB;SERPINA1;AMBP;FGFR4**
Cholangiocytes	Cholangiocytes	9/41	2.51952064242984E-05	0.000919625034486893	KRT19;CLDN4;ONECUT2;ELF3;CD24;SOX9;KRT8;AGR2;EPCAM
Enterocytes	Enterocytes	13/134	0.00251183106962623	0.0611212226942383	CDX2;PPARG;APOA1;GSTM3;ELF3;KLF5;FABP1;VIL1;CDH17;PRR15L;MTTP;PLIN2;APOB
Hepatoblasts	Hepatoblasts	4/17	0.00392229124466793	0.0715818152151897	KRT18;CDH1;AFP;KRT8
**Cholangiocyte-like**					
**Term**	**Term**	**Overlap**	**P-value**	**Adjusted P-value**	**Genes**
**Cholangiocytes**	**Cholangiocytes**	**7/41**	**0.00104113097136469**	**0.054138827765173**	**KRT19;CLDN4;KRT8;SPP1;TACSTD2;EPCAM;KRT7**
**Liver progenitor cell**	**Liver progenitor cell**	**2/2**	**0.00159231846368156**	**0.054138827765173**	**KRT19;KRT7**
Epiblast cells	Epiblast cells	4/16	0.00309509046760875	0.070155383932465	CDH1;CLDN7;EPCAM;CLDN6
Loop of Henle cells	Loop of Henle cells	4/25	0.0162412042334735	0.276100471969049	SPP1;IRX3;IRX2;IRX1
**Hepatic stellate cell-like**					
**Term**	**Term**	**Overlap**	**P-value**	**Adjusted P-value**	**Genes**
**Hepatic stellate cells**	**Hepatic stellate cells**	**10/43**	**5.05983077948034E-06**	**0.000308649677548301**	**RBP1;TPM2;BGN;PDGFRA;IGFBP3;COL1A2;OLFML3;TIMP1;COL3A1;COL1A1**
Mesangial cells	Mesangial cells	6/16	2.17579462324084E-05	0.000663617360088455	THY1;PDGFRB;LGALS1;FN1;SERPINE2;ZEB2
Fibroblasts	Fibroblasts	7/25	3.83680950607089E-05	0.000780151266234414	COL5A2;PTN;PDGFRA;COL3A1;FN1;LUM;COL1A1
Stromal cells	Stromal cells	4/7	7.91092686685066E-05	0.00120641634719473	COL3A1;COL6A3;LGALS1;COL1A2
**Embryonic stem cell-like**					
**Term**	**Term**	**Overlap**	**P-value**	**Adjusted P-value**	**Genes**
**Embryonic stem cells**	**Embryonic stem cells**	**11/77**	**0.000209419647093245**	**0.00617787958925071**	**BNIP3;PROM1;NANOG;SOX15;KIT;CD9;IL6ST;L1TD1;ITGA6;EPCAM;POU5F1**
Cancer stem cells	Cancer stem cells	6/19	6.66401539352373E-05	0.003931769082179	PDPN;PROM1;NANOG;KIT;EPCAM;POU5F1
Epiblast cells	Epiblast cells	3/16	0.0240228502278577	0.472449387814534	NANOG;EPCAM;POU5F1
Cancer cells	Cancer cells	2/8	0.0380207578497038	0.560806178283131	PROM1;EPCAM

Fig. R6: Example for the ULA-HLO annotations. For each cluster the top ranked annotation followed by secondary hits, including overlap, *p*-value, adjusted *p*-value and genes are provided.

e. Finally, the clustering parameters were fixed in such a way to obtain a number of clusters equal to the expected number of cell types. However, an unbiased criterion (e.g., based on cluster robustness) is much more desirable here, given that the difference in cell type identity and composition across conditions is one of the main points made in the paper.

We agree that an unbiased criterion is desirable here. We have evaluated our OS- and ULA-HLO dataset at varying Leiden resolutions utilizing *sklearn* functions *silhouette_score* and *davies_bouldin_score*. Silhouette coefficients are desired to be high (Rousseeuw, 1987, https://doi.org/10.1016/0377-0427(87)90125-7), Davies Bouldin scores to be low (Davies & Bouldin, *IEEE Trans Pattern Anal Mach Intell*, 1979; Yang et al., *BMC Genomics* 2017). In bold we indicate the maximum Silhouette coefficients and minimum Davies Bouldin indices dropping/increasing thereafter, based on which we reason that a number of clusters between 3 and 5 at a resolution of 0.1 is desirable for our datasets (**Table R1**).

OS-HLOs:

**resolution**	**number_of_clusters**	**sil**	**davie_bould**
0.1	3.0	**0.157987**	**1.783252**
0.2	5.0	0.146285	2.121505
0.3	8.0	0.110692	2.173497
0.4	9.0	0.098228	2.160852
0.5	10.0	0.101382	2.218449
0.6	10.0	0.081202	2.806286
0.7	10.0	0.059232	2.685656
0.8	10.0	0.043528	2.827449
0.9	10.0	0.039771	2.841200
1.0	10.0	0.039274	2.786283
1.1	10.0	0.044187	2.865855
1.2	10.0	0.038195	2.764087
1.3	10.0	0.022657	2.634697
1.4	10.0	0.025554	2.743233
1.5	10.0	0.024600	2.739556

ULA HLOs:

resolution	number_of_clusters	sil	davie_bould
0.1	4.0	**0.248157**	**1.413687**
0.2	6.0	0.168204	1.775483
0.3	7.0	0.171748	1.694957
0.4	10.0	0.134452	1.979794
0.5	10.0	0.122700	2.003697
0.6	10.0	0.122285	2.041422
0.7	10.0	0.115831	2.042962
0.8	10.0	0.121401	2.006257
0.9	10.0	0.123157	2.004311
1.0	10.0	0.105311	2.075387
1.1	10.0	0.107451	2.068708
1.2	10.0	0.107771	2.078919
1.3	10.0	0.104997	2.152287
1.4	10.0	0.098598	2.162105
1.5	10.0	0.089793	2.189535

Table R1: Silhouette and Davies-Bouldin scores for different Leiden algorithm resolutions in OS- and ULA-HLOs.

2. Cell abundance testing. Multiple claims are made about differences in cell abundance between conditions, or along trajectories. The authors should perform statistical tests to verify these claims and provide a measure of statistical significance (taking into account the number of cells, replicates, and giving statistical uncertainty). There are several packages available to do that (e.g., https://www.nature.com/articles/s41467-021-27150-6).

We find this suggestion exciting and performed the testing recommended by the reviewer using scCODA (Büttner, Ostner et al., 2021, https://doi.org/10.1038/s41467-021-27150-6). We find credible effects at an **FDR of 0.05** for the following cell types. We conclude that our previous interpretations on HSC-like abundances and the expansion of ductal cells with TGF-β1 remain valid, and this is also the case for the HSC-like cell decrease and FH/HB-like expansion with OA and the absence of those effects with PA. We have appended these results to our **Supplementary_Table_3_Cellshifts-OAPATGFB1.csv** and included the significance status in our results section.

Covariate	Cell Type	FDR<0.05
**controlstatus[T.OA500]**		
	FH1	True
	HB2	True
	Hepatic stellate cells	True
**controlstatus[T.PA]**		
	/	/

controlstatus[T.TGFB1]

Ductal cells	True
Hepatic stellate cells	True
Smooth muscle cells	True

Table R2: scCODA outputs for differential abundance testing in OS-HLOs treated with OA, PA and TGF-β1, and their controls

3. Organoid variability. Their analysis shows that there's some inter-organoid variability, as seen, for example, from the cell-type composition of OS organoids shown in Figure 3c. We think there are two important points to consider: a. It has been observed that organoid variability can affect conclusions on organoid-based perturbation studies (https://www.biorxiv.org/content/10.1101/2022.09.27.509783v1). The authors should discuss if and how organoid variability can affect their claims, especially in cases with fewer replicates (e.g., in Figure 4 where they have N=2 replicates per condition).

We agree that batch variability is a challenge in organoid culture, which is why we always differentiate controls along with the treatment samples in each generation. This approach did reduce the number of replicates that could be analyzed with current resources. We have inserted a separate paragraph in the discussion highlighting limitations of the study and added these points. Please also find additional qRT-PCR results from new differentiation rounds supporting our major claims on cell type shifts from HLOs differentiated from two different PSC lines (**Supplementary Fig. 4g-h**).

b. In the methods section, they say that "Clusters were required to represent all individual replicates". First, it is unclear what this means: what do they do if the algorithm identifies a cluster that does not include cells from all replicates? is it merged with others? If so, how? Second, this approach might lead to an underestimation of inter-organoid variability. The authors should perform clustering without imposing such constraint so that a more unbiased estimation of organoid variability is obtained.

We apologize for not being sufficiently clear on this topic. When analyzing OS-HLOs, the Leiden algorithm identified two clusters of 705 and 12 cells that were not present in all control samples. In this case, the respective cells were ***excluded*** from the analysis which we have now clarified in the **Methods** section and documented in detail in **Supplementary_Table_10_QC-metrics.ods**. This table also contains the **marker genes** characterizing these clusters. We also provide a **UMAP** with all clusters (**Fig. R7**). Our aim was to ensure we report only cell types robust to inter-batch variability (Andrews and Hemberg, *Molec Asp Med*, 2018). However, we agree that inter-batch organoid variability is an issue, a point that we have highlighted in the limitations paragraph of our discussion.

OS-HLOs all clusters			
	Pre-cluster filtering	Post-cluster filtering	
0	50043	50043	
1	16666	16666	
2	13005	13005	
3	2753	2753	
4	705	excluded	
5	12	excluded	
Total cells	83184	82467	
Cluster 4			
sample	culture	treatment	n cells
50	OS	CTRL-TGFB1	21
51	OS	CTRL-TGFB1	14
52	OS	TGFB1	9
53	OS	TGFB1	6
62	OS	CTRL-PA	93
63	OS	CTRL-PA	133
64	OS	PA	236
65	OS	PA	155
SM-L3XWE	OS	CTRL	26
SM-L3XWF	OS	CTRL	3
SM-L3XWG	OS	CTRL-OA	1
SM-L3XWH	OS	CTRL-OA	0
SM-L3XWI	OS	OA	3
SM-L3XWJ	OS	OA	5
Total cells			705
Cluster 5			
sample	culture	treatment	n cells
50	OS	CTRL-TGFB1	0
51	OS	CTRL-TGFB1	0
52	OS	TGFB1	0
53	OS	TGFB1	0
62	OS	CTRL-PA	0
63	OS	CTRL-PA	0
64	OS	PA	0
65	OS	PA	0
SM-L3XWE	OS	CTRL	0
SM-L3XWF	OS	CTRL	0
SM-L3XWG	OS	CTRL-OA	0
SM-L3XWH	OS	CTRL-OA	0
SM-L3XWI	OS	OA	0
SM-L3XWJ	OS	OA	12
Total cells			12

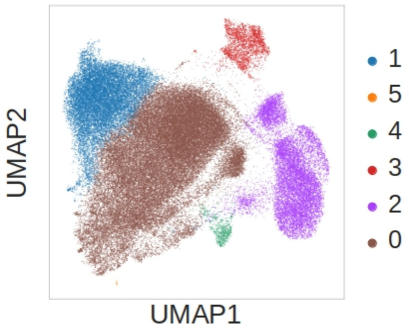

Fig. R7: Information on clusters excluded due to limited recovery of all replicates (left) and UMAP representation of the clusters (right) in OS-HLOs.

4. Organoid day. Organoids were grown until day 21 in all conditions. Could some of the differences they observed across conditions be due to the fact that the development of the organoid becomes slower/faster in different conditions? And would this impact their conclusions?

We understand the reviewers' concern that the treatment effects observed could be due to delays or accelerations in development speed. However, the occurrence of inflammatory signals or the generation of a fibrotic phenotype, including a potential transdifferentiation of hepatoblasts into HSCs or vice versa, is not expected throughout maturation of any of these cell types. Therefore, even if the maturation was accelerated it would most likely not affect our general conclusions on the acquisition of fibrotic and inflammatory gene signatures in the treatment conditions.

5. Communication analysis. The authors show that there are differences in cell communication across different conditions. This is interesting, but they should perform a statistical analysis of these results and list the communication patterns that are statistically significant.

We thank the reviewers for this suggestion and have now extended our analysis. We performed a groupwise Mann-Whitney-U statistics to infer cell-cell pairs with significant changes in relative interaction abundance (taking into account individual replicates). We have listed significantly

changed communication patterns in **Supplementary_Table_11_Interactions_Mann_whitney_u_stats.tsv**. We note that the power of such an analysis is potentially low due to our limited number of replicates per treatment condition, a limitation that we have also highlighted in our discussion.

6. Data Imputation. They perform data imputation for the analysis in Figure 6g with MAGIC. Using data-smoothing-based methods (like MAGIC) has been associated with an increase in false positives (https://www.ncbi.nlm.nih.gov/pmc/articles/PMC6415334/). Is data-imputation necessary for this analysis? Can it be avoided?

We agree that the study by Andrews and Hemberg is important to keep in mind when interpreting the results showing imputed expression values. We utilized Palantir for pseudotime calculation and applied MAGIC imputation in order to stick to the default workflow (https://nbviewer.org/github/dpeerlab/Palantir/blob/master/notebooks/Palantir_sample_notebook.ipynb), and found imputation helpful to visualize the acquisition of inflammatory and fibrotic signatures in HLO subpopulations with individual treatments. To address the concern, we provide expression matrix plots of normalized and imputed gene expression for the genes from Fig. 6g, allowing us to compare both levels. We focus on the DC1- and SMC-like terminal states since they display the most distinct enrichment of inflammation- and fibrosis-related transcripts, and find the imputed expression in line with the normalized expression (**Fig. R8-9**). We have not included this analysis in the manuscript, but we can add it to the supplementary figures if preferred.

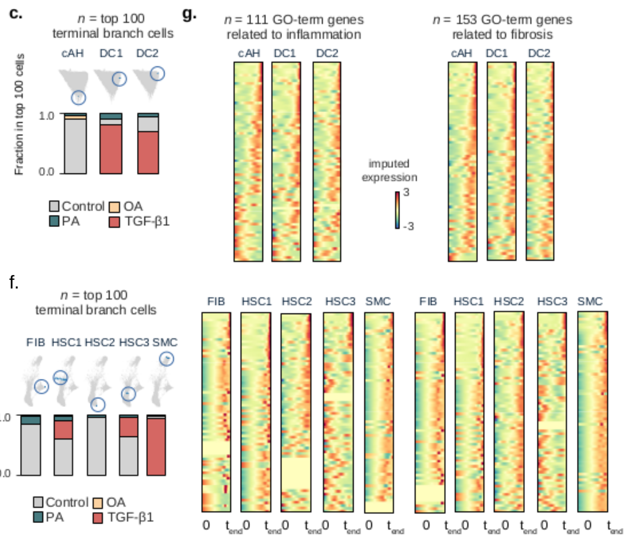

Fig. R8: Snapshot of the original Figure 6c,f,g for reference.

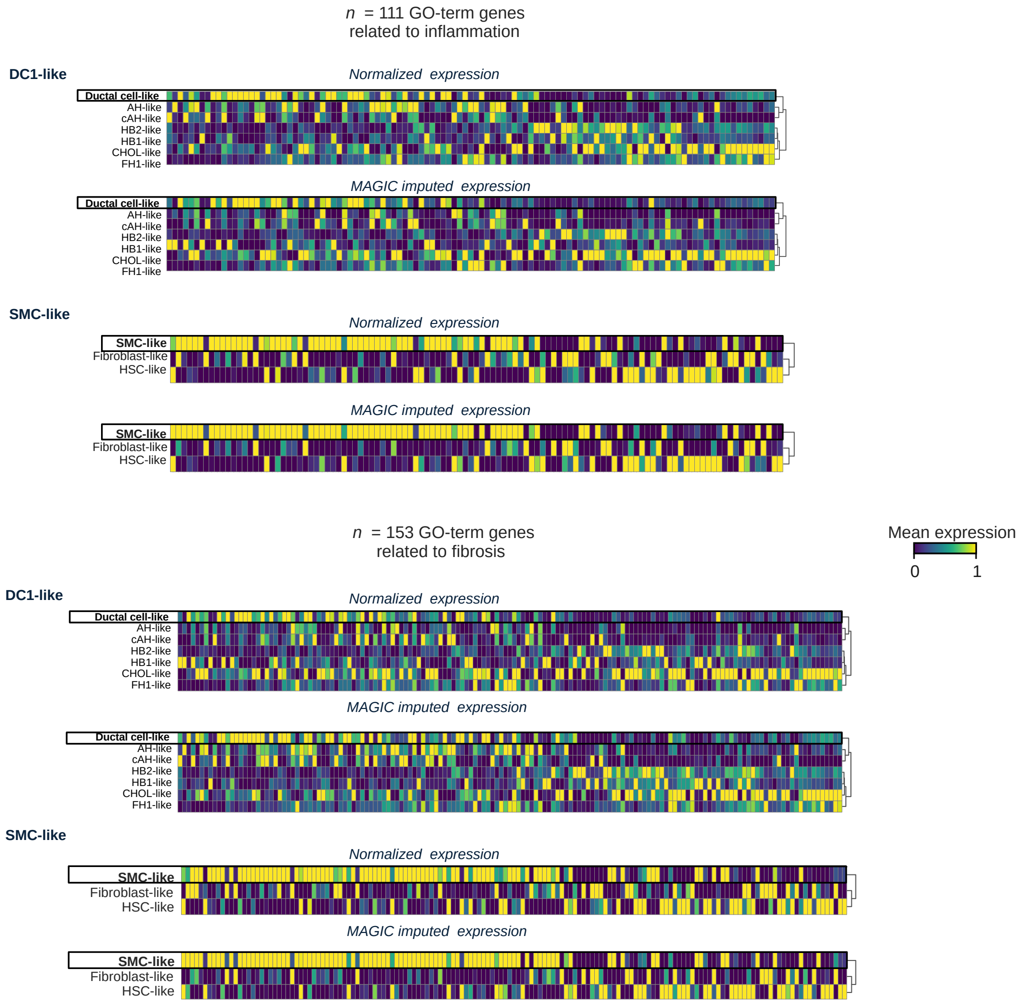

Fig. R9: Comparison of normalized expression and imputed expression for two major terminal states in OS-HLOs. **a.** Projection of the top 100 terminal branch cells (sorted by branch probabilities) for two terminal states identified by Palantir on the ForceAtlas2 representation (top). Corresponding barplots below displaying the relative distribution of treatment conditions among the top 100 terminal branch cells for each terminal state. b. Heatmaps showing the imputed gene expression over Palantir pseudotime for GO-term-derived inflammation (left) and fibrosis (right)-related genes sorted by their imputed expression level at each terminal state (indicated on top of each heatmap). Imputed expression levels are indicated by color, y-axes correspond to genes, and x-axes display the pseudotime. Ranked gene lists are provided in Supplementary Table 8. **c.** Matrix plots displaying standard normalized mean expression (top) along with imputed gene expression (MAGIC, bottom) for genes identified to enrich towards pseudotime for the DC1- and SMC-like terminal states (genes on the x-axis are the same as in y-axis of b). Categorical ordering by cell type. Dendrogram on the right displaying hierarchical clustering.

7. Methods. There are a few points in the Methods section that need clarification or additions. a. Why putting a max (rather than min) threshold on the number of counts (30k)? The usual approach is to impose a threshold on the minimum number of counts.

We apologize for not having mentioned our **minimum of 500 counts**, which we have inserted into the Methods section. We chose a **maximum of 30k counts after manual inspection of QC metrics violin plots** in order keep the cells roughly inside the **1.5*inter-quartile range** of the count distribution (**Fig. R10**).

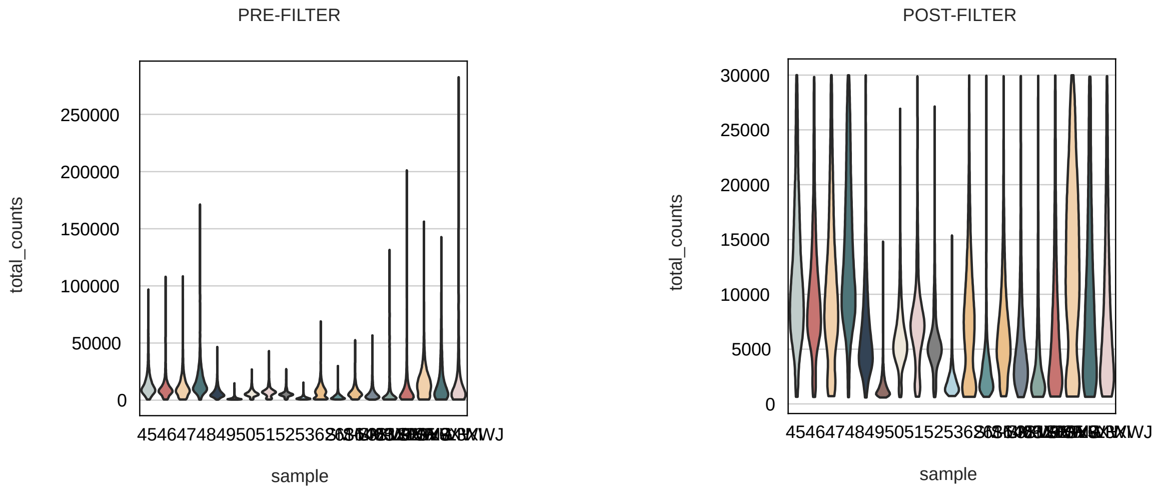

Fig. R10: Pre- and post-QC filtering violin plots utilized for selection of count thresholds.

b. how do they select highly variable genes?

Highly variable genes were selected using the *sc.pp.highly_variable_genes* function with the *n_top_genes* parameter set to 5000. We have now specified this in the Methods section accordingly.

c. what do they mean by scaling the expression values to a max value of 5? Is it a trimming (i.e., whatever is above 5 gets assigned a value of 5)?

Each gene was transformed to unit variance by applying the *sc.pp.scale* function with the *max_value* parameter set to 5, indicating that values exceeding 5 were being clipped. We have now specified this in the Methods section accordingly.

d. The number (and fraction) of cells removed after QC in each dataset should be reported.

We have now added **Supplementary_Table_10_QC-metrics.ods** reporting number and fraction of cells removed after QC in each dataset. Additionally, we have added the summarized numbers to the **Methods** section.

sample	culture	treatment	n_cells_preQC	n_cells_postQC	cells_removed	percentage_removed
45	ULA	CTRL	6583	5472	1111	16.88
46	ULA	CTRL	7362	5330	2032	27.60
47	ULA	CTRL	7122	6033	1089	15.29
50	OS	CTRL-TGFB1	11879	9506	2373	19.98
51	OS	CTRL-TGFB1	9370	8417	953	10.17
52	OS	TGFB1	6729	5919	810	12.04
53	OS	TGFB1	7374	6839	535	7.26
62	OS	CTRL-PA	4051	3093	958	23.65
63	OS	CTRL-PA	6561	5510	1051	16.02
64	OS	PA	8634	7399	1235	14.30
65	OS	PA	9073	8032	1041	11.47
SM-L3XWE	OS	CTRL	10093	9356	737	7.30
SM-L3XWF	OS	CTRL	7905	5514	2391	30.25
SM-L3XWG	OS	CTRL-OA	6857	5011	1846	26.92
SM-L3XWH	OS	CTRL-OA	3525	2604	921	26.13
SM-L3XWI	OS	OA	3438	2514	924	26.88
SM-L3XWJ	OS	OA	3630	2753	877	24.16
	**preQC**	**post QC**	**removed**	**percentage_removed**		
**ULA**	**21067**	**16835**	4232	20.09		
**OS_CTRLS**	**60241**	**49011**	11230	18.64		
**OS_ALL**	**99119**	**82467**	16652	16.80		

Fig. R11: Representative image of Supplementary Table 10 contents reporting QC metrics.

8. The authors highlight that "integrating data from each model with that of NAFLD patients across disease progression further demonstrates PA and TGF-β1 more robustly model inflammation and fibrosis" (page 2). We think that this claim is misleading. What the authors do instead is use a scoring system that is based on 26 and 98 marker genes, respectively, to assess and score the severity of the different NAFLD models. A way to keep the claim of an integration approach would be to actually integrate scRNAseq data from healthy or diseased donors, and compare composition of different cell types. Indeed, if such data were available, this would be very interesting for the reader and a good possibility to directly evaluate how well disease models represent patient data

We thank the reviewer for their comment and have now clarified the claim by correcting our terminology in the section mentioned. There are not yet sufficient scRNAseq datasets available across steatohepatitis and fibrosis in human samples for the integration.

9. In the data availability statement, the authors write: "The following custom scripts are available upon request and will be made publicly available upon release.". What are the "following custom scripts" that will be made publicly available? Did the authors intend to list the scripts? Moreover, the code should have been made available to Reviewers before publication so that reproducibility could be verified.

We thank the reviewer for their comment and attach the repositories as zipped files with this submission. We also provide a Dropbox link to the two repositories for alternative access: https://www.dropbox.com/scl/fo/j6g3xnt01kgcuplchnwle/h?dl=0&rlkey=hit5gi1nhj1ku8ruj0ipxurhc

Minor comments

- Figure 1:

- Adding axes would greatly improve readability, e.g. Fig. 1b,f, also 2 i-k

- f: Unify legends for all three plots (odds ratio, p-value). Color of dots is not always as shown in the legend - is this a continuous scale? Please change accordingly.

We have edited the figures taking into account these suggestions for improved readability.

- Figure 2:

- Size of in-situ images should be increased

- Tilt 2g by 90{degree sign} to make axes the same as in 2 f to improve readability

We appreciate this suggestion and enlarged in-situ images whenever possible, and tilted **Fig. 2g**.

- Figure 3:

- What do the authors mean by "broader distribution of HSC marker genes in ULA-HLOs" ?

We clarified that we find less expression of genes such as *SPARC, COL3A1, COL1A1* in OS-HLOs when compared to ULA-HLOs. We now also refer to **Supplementary Fig. 2g** as described below to clarify this comment.

- d: To compare marker gene expression for different conditions, rather use violin plots for cell types and conditions.

We thank the reviewer for their suggestion and provide the genes from d in violin plot views in **Supplementary Fig. 2g**.

- h: Does the portion of zones in the organoid go in accordance with in vivo data?

Based on analysis of scRNA-seq and FISH in mouse livers (Halpern et al., 2017, 10.1038/nature21065, Extended Data 3), we would anticipate more cells in the interzonal area (region 2-3, Fig. 2h) than either the more periportal (regions 0-1) or pericentral regions (regions 4-5). This is what we observe. We would also anticipate more periportal hepatocytes than pericentral hepatocytes, as we also observe, but the relative number of pericentral hepatocytes is lower than what would be anticipated from Halpern et al. We have added a comment about these relative numbers to the discussion. Pericentral hepatocytes are in a relatively oxygen and nutrient low niche compared to pericentral and interzonal hepatocytes. This gradient of oxygen and nutrients has not been evaluated in HLOs and may not exist to the same degree in HLOs.

- How are the conditions OS and ULA transcriptionally different that could explain their observed phenotypes in culture? What is the conclusion of the authors regarding the similarity of these models to NAFLD in patients?

We have also added a discussion paragraph on cell type distributions in the human liver and organoid models.

- Figure 4:

- d: Controls show induction of connective tissue response to inflammation as well as fibroblast migration. How do the authors explain that?

We thank the reviewer for their comment. We think a baseline activity for “fibroblast migration” and “connective tissue responses” is expected in HSC-like cells, and those show the highest enrichment for this score in control conditions. Additionally, background activity of response pathways may be attributed to stimuli during the preparation of a single-cell suspension (dissociation) prior to scRNA-seq.

- f: Readability can be improved. It's not clear which bars belong to which labels. —

We thank the reviewer for their suggestion and have now added lines connecting labels and bars.

- Also, it would be interesting to see which of the terms come up in all conditions (overlap), and which are specific to one disease model.

We thank the reviewer for their suggestion and have added a sheet to **Supplementary_Table_5_DGE-treatments.ods** containing overlap and specific terms.

- Figure 5:

Instead of a venn diagram, the use of an upset plot to visualize the overlap between ligand-receptor interactions might improve clarity

We appreciate the reviewer’s suggestion and have added an upset plot in **Supplementary Fig. 6c**.

- Figure 7:

The way the authors present this analysis could summarize and emphasize the effect of the different models better. How about summarizing the scores in a dotplot or heatmap with cell types and models as axes instead of multiple violin plots?

We appreciate the reviewer’s suggestion and have added the categorical heatmaps to **Supplementary Fig. 7b**.

1st Revision - Editorial Decision — 10th Sep 2023

Dear Alan,

Thank you for submitting your revised manuscript (EMBOJ-2023-113898R) to The EMBO Journal, as well as for your patience with our response at this time of the year. Your amended study was sent back to the referees for their re-evaluation, and we have received comments from all of them, which I enclose below.

As you will see, the experts stated that the work has been substantially improved by the revisions and they are now broadly in favour of publication, pending minor revision.

Thus, we are pleased to inform you that your manuscript has been accepted in principle for publication in The EMBO Journal.

Please consider the remaining minor points by referee #3 carefully and amend the text and file collection where appropriate.

Also, we now need you to take care of a number of issues related to formatting and data presentation as detailed below, which should be addressed at re-submission.

Please contact me at any time if you have additional questions related to below points.

As you might have noted on our web page, every paper at the EMBO Journal now includes a 'Synopsis', displayed on the html and freely accessible to all readers. The synopsis includes a 'model' figure as well as 2-5 one-short-sentence bullet points that summarize the article. I would appreciate if you could provide this figure and the bullet points.

Thank you for giving us the chance to consider your manuscript for The EMBO Journal. I look forward to your final revision.

Again, please contact me at any time if you need any help or have further questions.

Best regards,

Daniel

Daniel Klimmeck PhD
Senior Editor
The EMBO Journal

****

Formatting changes required for the revised version of the manuscript:

>> Please limit the number of keywords for your study to maximally five.

>> Reduce the abstract length to max. 175 words.

>> Adjust the title of the 'Declaration of Interests' section to 'Disclosure and Competing Interests Statement'.

>> Author Contributions: Please remove the author contributions information from the manuscript text. Note that CRediT has replaced the traditional author contributions section as of now because it offers a systematic machine-readable author contributions format that allows for more effective research assessment. and use the free text boxes beneath each contributing author's name to add specific details on the author's contribution.
More information is available in our guide to authors.
https://www.embopress.org/page/journal/14602075/authorguide

>> Please adjust the reference format to EMBO Journal style, limiting to 10 authors et al. .

>> Manuscript order: main figure legends need to be provided in the main manuscript file, after the References.

>> Appendix file: amend the ToC on the first page with page numbers. Change the nomenclature to Appendix Figure S1-S2... and Appendix Table S1-... and adjust the corresponding callouts in the text.

>> Dataset EV Legends: Suppl. Tables 1-11 should be unzipped and each uploaded as a dataset; the nomenclature is Dataset EV1, etc. callouts need to be updated accordingly; the legends need to be removed from the manuscript and inserted in each Excel file (as a separate sheet).

>> The two zipped files, Code scRNA-seq analysis and Code Sirius red analysis, need to be removed from the manuscript.

>>Please include the financial support and funding information in the Acknowledgements section.

>> Provide Source Data compiled as one file per figure (compare attached source data request summary by my colleague H. Sonntag).

>> Main figure files (without the legends) should be uploaded separately as individual Figure Files; Supplementary figures can be provided in the Appendix file.

>> Data accessibility section: please remove the referee token from the text and make sure privacy is released for the GEO dataset.

>>Complete the author checklist, filling information on human donor information in the 'Materials' and 'Ethics' ethics sections.

>> Consider additional changes and comments from our production team as indicated by the .doc file enclosed and leave changes in track mode.

Further information is available in our Guide For Authors: https://www.embopress.org/page/journal/14602075/authorguide

Please use the link below to submit your revision:

https://emboj.msubmit.net/cgi-bin/main.plex

Referee #1:

Hess et al. have carefully revised their manuscript taking into account the reviewers' comments. My concerns have been satisfactorily addressed. I have no further comments.

Referee #2:

The authors addressed all my points and should be congratulated to this interesting work.

Referee #3:

Overall, we are very satisfied with how the authors addressed our comments, and how they improved the manuscript's clarity. There are only a few remaining points that we recommend addressing, as detailed below (we are keeping the same numbering used in the rebuttal).
Most of them are about adding to the paper some of the additional analyses the authors performed.

1. Cell type annotation:
c: We think it would be helpful for the reader to mention in the text that stem cells were searched also by using an algorithm specifically designed to detect rare cells, and that nothing could be found.
e: Either of the metrics they computed to support the choice of the number of clusters should be plotted and added as a Supp. figure.

6. Data imputation: It would be good to include Fig. R9 to the Supp. Material to show a few examples of how imputed data differ from the original data.

9. Data and code availability: We very much appreciate the inclusion of the python scripts used for the analysis. To make the results even more accessible and reproducible for a wider audience, we recommend publishing alongside the paper a simple

notebook (e.g., a jupyter notebook) to execute all the basic data processing like filtering, normalization, scaling, marker genes identification etc. These are fundamental steps for the analysis, and being able to correctly carry them out would be crucial to anyone who wants to further explore the data.

From the minor comments:
- Figure3: If the authors want to compare marker gene expression between the ULA and OS conditions, violin plots are better suited than the 2-dimensional embedding shown in Figure 2d; hence, we suggest replacing Figure 2d with the violin plots. If the authors want to emphasize the expression of marker genes in specific cell clusters like Hepatic stellate cell-like, they could compare the expression of marker genes just in these cell types.
- Figure 5: Actually, we were suggesting replacing the Venn diagram shown in Figure 5e (and not Figure 5f) with an upset plot, which is typically more convenient to visualize the overlap of more than three classes.

Response Letter #2

Date: 2023-SEPT-27

Referee #1: Hess et al. have carefully revised their manuscript taking into account the reviewers' comments. My concerns have been satisfactorily addressed. I have no further comments.

We thank the reviewer for their support and thoughtful comments.

Referee #2: The authors addressed all my points and should be congratulated to this interesting work.

We thank the reviewer for their support and thoughtful comments.

Referee #3: Overall, we are very satisfied with how the authors addressed our comments, and how they improved the manuscript's clarity. There are only a few remaining points that we recommend addressing, as detailed below (we are keeping the same numbering used in the rebuttal). Most of them are about adding to the paper some of the additional analyses the authors performed.

We thank the reviewer for their support and thoughtful comments and will refer to the individual points below.

1. Cell type annotation:

c: We think it would be helpful for the reader to mention in the text that stem cells were searched also by using an algorithm specifically designed to detect rare cells, and that nothing could be found.

We have inserted a sentence into the main text.

e: Either of the metrics they computed to support the choice of the number of clusters should be plotted and added as a Supp. Figure.

We added **Appendix Fig. S2g** displaying both metrics.

6. Data imputation: It would be good to include Fig. R9 to the Supp. Material to show a few examples of how imputed data differ from the original data.

We have included the figure as **Appendix Fig. S7**.

9. Data and code availability: We very much appreciate the inclusion of the python scripts used for the analysis. To make the results even more accessible and reproducible for a wider audience, we recommend publishing alongside the paper a simple notebook (e.g., a jupyter notebook) to execute all the basic data processing like filtering, normalization, scaling, marker genes identification etc.

These are fundamental steps for the analysis, and being able to correctly carry them out would be crucial to anyone who wants to further explore the data.
We have added a **jupyter notebook** to with analysis steps to our Github repository.

From the minor comments:
- Figure3: If the authors want to compare marker gene expression between the ULA and OS conditions, violin plots are better suited than the 2-dimensional embedding shown in Figure 2d; hence, we suggest replacing Figure 2d with the violin plots. If the authors want to emphasize the expression of marker genes in specific cell clusters like Hepatic stellate cell-like, they could compare the expression of marker genes just in these cell types.
We have replaced main **Fig. 2d** with the violin plots as suggested.

- Figure 5: Actually, we were suggesting replacing the Venn diagram shown in Figure 5e (and not Figure 5f) with an upset plot, which is typically more convenient to visualize the overlap of more than three classes.
We have now replaced **Fig. 5e** with an UpSet plot as suggested.

Additional changes in response to requests from the first revision:
- Figure 7: The way the authors present this analysis could summarize and emphasize the effect of the different models better. How about summarizing the scores in a dotplot or heatmap with cell types and models as axes instead of multiple violin plots?
We have now also replaced our main **Fig. 7a** and **Fig. 7c** with dotplots and pairwise comparisons to summarize the model effects better, in addition to the categorical heatmaps in Appendix Fig. S7b added in the previous revision.

2nd Revision - Editorial Decision 6th Oct 2023

Dear Dr Alan Mullen,

Thank you for submitting the revised version of your manuscript. I have now evaluated your amended manuscript and concluded that the remaining minor concerns have been sufficiently addressed.

Thus, I am pleased to inform you that your manuscript has been accepted for publication in the EMBO Journal.

Please note that it is EMBO Journal policy for the transcript of the editorial process (containing referee reports and your response letter) to be published as an online supplement to each paper. I would accordingly like to ask for your consent on keeping the additional referee figures included in this file.

Also, in case you might NOT want the transparent process file published at all, you will also need to inform us via email immediately. More information is available here:
https://www.embopress.org/page/journal/14602075/authorguide#transparentprocess

----

Please note that in order to be able to start the production process, our publisher will need and contact you shortly regarding the page charge authorisation and licence to publish forms.

Authors of accepted peer-reviewed original research articles may choose to pay a fee in order for their published article to be made freely accessible to all online immediately upon publication. The EMBO Open fee is fixed at $6,540 USD / £5,310 GBP / €5,900 EUR (+ VAT where applicable).

Should you be planning a Press Release on your article, please get in contact with embojournal@wiley.com as early as possible, in order to coordinate publication and release dates.

On a different note, I would like to alert you that EMBO Press is currently developing a new format for a video-synopsis of work published with us, which essentially is a short, author-generated film explaining the core findings in hand drawings, and, as we believe, can be very useful to increase visibility of the work. This has proven to offer a nice opportunity for exposure i.p. for the first author(s) of the study. Please see the following link for representative examples and their integration into the article web page:
https://www.embopress.org/video_synopses
https://www.embopress.org/doi/full/10.15252/embj.2019103932

Please let me know, should you be interested to engage in commissioning a similar video synopsis for your work. According operation instructions are available and intuitive.

Finally, we have noted that the submitted version of your article is also posted on the preprint platform bioRxiv. We would appreciate if you could alert bioRxiv on the acceptance of this manuscript at The EMBO Journal in order to allow for an update of the entry status. Thank you in advance!

If you have any questions, please do not hesitate to call or email the Editorial Office.

Thank you again for this contribution to The EMBO Journal and congratulations on a successful publication! Please consider us again in the future for your most exciting work.

Best regards,

Daniel Klimmeck

Daniel Klimmeck, PhD
Senior Editor
The EMBO Journal
EMBO

Postfach 1022-40
Meyerhofstrasse 1
D-69117 Heidelberg
contact@embojournal.org
Submit at: http://emboj.msubmit.net

** Click here to be directed to your login page: https://emboj.msubmit.net

EMBO Press Author Checklist

Corresponding Author Name: Alan C. Mullen
Journal Submitted to: EMBO J.
Manuscript Number: EMBOJ-2023-113898R

USEFUL LINKS FOR COMPLETING THIS FORM
The EMBO Journal - Author Guidelines
EMBO Reports - Author Guidelines
Molecular Systems Biology - Author Guidelines
EMBO Molecular Medicine - Author Guidelines

Reporting Checklist for Life Science Articles (updated January
This checklist is adapted from Materials Design Analysis Reporting (MDAR) Checklist for Authors. MDAR establishes a minimum set of requirements in transparent reporting in the life sciences (see Statement of Task: 10.31222/osf.io/9sm4x). Please follow the journal's guidelines in preparing your
Please note that a copy of this checklist will be published alongside your article.

Abridged guidelines for figures
1. Data
The data shown in figures should satisfy the following conditions:
- the data were obtained and processed according to the field's best practice and are presented to reflect the results of the experiments in an accurate and unbiased manner.
- ideally, figure panels should include only measurements that are directly comparable to each other and obtained with the same assay.
- plots include clearly labeled error bars for independent experiments and sample sizes. Unless justified, error bars should not be shown for technical
- if n<5, the individual data points from each experiment should be plotted. Any statistical test employed should be justified.
- Source Data should be included to report the data underlying figures according to the guidelines set out in the authorship guidelines on Data

2. Captions
Each figure caption should contain the following information, for each panel where they are relevant:
- a specification of the experimental system investigated (eg cell line, species name).
- the assay(s) and method(s) used to carry out the reported observations and measurements.
- an explicit mention of the biological and chemical entity(ies) that are being measured.
- an explicit mention of the biological and chemical entity(ies) that are altered/varied/perturbed in a controlled manner.
- the exact sample size (n) for each experimental group/condition, given as a number, not a range;
- a description of the sample collection allowing the reader to understand whether the samples represent technical or biological replicates (including how many animals, litters, cultures, etc.).
- a statement of how many times the experiment shown was independently replicated in the laboratory.
- definitions of statistical methods and measures:
  - common tests, such as t-test (please specify whether paired vs. unpaired), simple χ2 tests, Wilcoxon and Mann-Whitney tests, can be unambiguously identified by name only, but more complex techniques should be described in the methods section;
  - are tests one-sided or two-sided?
  - are there adjustments for multiple comparisons?
  - exact statistical test results, e.g., P values = x but not P values < x;
  - definition of 'center values' as median or average;
  - definition of error bars as s.d. or s.e.m.

Please complete ALL of the questions below.
Select "Not Applicable" only when the requested information is not relevant for your study.

Materials

Newly Created Materials	Information included in the manuscript?	In which section is the information available? (Reagents and Tools Table, Materials and Methods, Figures, Data Availability Section)
New materials and reagents need to be available; do any restrictions apply?	Not Applicable	

Antibodies	Information included in the manuscript?	In which section is the information available? (Reagents and Tools Table, Materials and Methods, Figures, Data Availability Section)
For **antibodies** provide the following information:<br>- Commercial antibodies: RRID (if possible) or supplier name, catalogue number and or/clone number<br>- Non-commercial: RRID or citation	Yes	Materials and Methods

DNA and RNA sequences	Information included in the manuscript?	In which section is the information available? (Reagents and Tools Table, Materials and Methods, Figures, Data Availability Section)
**Short novel DNA or RNA including primers, probes**: provide the sequences.	Yes	Materials and Methods

Cell materials	Information included in the manuscript?	In which section is the information available? (Reagents and Tools Table, Materials and Methods, Figures, Data Availability Section)
**Cell lines:** Provide species information, strain. Provide accession number in repository **OR** supplier name, catalog number, clone number, and/**OR** RRID.	Yes	Materials and Methods
**Primary cultures:** Provide species, strain, sex of origin, genetic modification status.	Not Applicable	
Report if the cell lines were recently **authenticated** (e.g., by STR profiling) and tested for mycoplasma contamination.	Yes	Materials and Methods

Experimental animals	Information included in the manuscript?	In which section is the information available? (Reagents and Tools Table, Materials and Methods, Figures, Data Availability Section)
**Laboratory animals or Model organisms:** Provide species, strain, sex, age, genetic modification status. Provide accession number in repository **OR** supplier name, catalog number, clone number, **OR** RRID.	Not Applicable	
**Animal observed in or captured from the field:** Provide species, sex, and age where possible.	Not Applicable	
Please detail **housing and husbandry conditions**.	Not Applicable	

Plants and microbes	Information included in the manuscript?	In which section is the information available? (Reagents and Tools Table, Materials and Methods, Figures, Data Availability Section)
**Plants:** provide species and strain, ecotype and cultivar where relevant, unique accession number if available, and source (including location for collected wild specimens).	Not Applicable	
**Microbes:** provide species and strain, unique accession number if available, and source.	Not Applicable	

Human research participants	Information included in the manuscript?	In which section is the information available? (Reagents and Tools Table, Materials and Methods, Figures, Data Availability Section)
If collected and within the bounds of privacy constraints report on age, sex and gender or ethnicity for all study participants.	Yes	Materials and Methods

Core facilities	Information included in the manuscript?	In which section is the information available? (Reagents and Tools Table, Materials and Methods, Figures, Data Availability Section)
If your work benefited from core facilities, was their service mentioned in the acknowledgments section?	Yes	Acknowledgments

Design

Study protocol	Information included in the manuscript?	In which section is the information available? (Reagents and Tools Table, Materials and Methods, Figures, Data Availability Section)
If study protocol has been **pre-registered, provide DOI in the manuscript**. For clinical trials, provide the trial registration number **OR** cite DOI.	Not Applicable	
Report the **clinical trial registration number** (at ClinicalTrials.gov or equivalent), where applicable.	Not Applicable	

Laboratory protocol	Information included in the manuscript?	In which section is the information available? (Reagents and Tools Table, Materials and Methods, Figures, Data Availability Section)
Provide DOI OR other citation details if **external detailed step-by-step protocols** are available.	Yes	Materials and Methods, References

Experimental study design and statistics	Information included in the manuscript?	In which section is the information available? (Reagents and Tools Table, Materials and Methods, Figures, Data Availability Section)
Include a statement about **sample size** estimate even if no statistical methods were used.	Not Applicable	
Were any steps taken to minimize the effects of subjective bias when allocating animals/samples to treatment (e.g. **randomization procedure**)? If yes, have they been described?	Not Applicable	
Include a statement about **blinding** even if no blinding was done.	Not Applicable	
Describe **inclusion/exclusion criteria** if samples or animals were excluded from the analysis. Were the criteria pre-established?<br><br>If sample or data points were omitted from analysis, report if this was due to attrition or intentional exclusion and provide justification.	Not Applicable	
For every figure, are **statistical tests** justified as appropriate? Do the data meet the assumptions of the tests (e.g., normal distribution)? Describe any methods used to assess it. Is there an estimate of variation within each group of data? Is the variance similar between the groups that are being statistically compared?	Yes	Statistics and reproducibility

Sample definition and in-laboratory replication	Information included in the manuscript?	In which section is the information available? (Reagents and Tools Table, Materials and Methods, Figures, Data Availability Section)
In the figure legends: state number of times the experiment was **replicated** in laboratory.	Yes	Figure legends
In the figure legends: define whether data describe **technical or biological replicates**.	Yes	Figure legends

Ethics

Ethics	Information included in the manuscript?	In which section is the information available? (Reagents and Tools Table, Materials and Methods, Figures, Data Availability Section)
Studies involving **human participants**: State details of **authority granting ethics approval** (IRB or equivalent committee(s), provide reference number for approval.	Yes	Materials and Methods
Studies involving **human participants**: Include a statement confirming that **informed consent** was obtained from all subjects and that the experiments conformed to the principles set out in the WMA Declaration of Helsinki and the Department of Health and Human Services Belmont Report.	Not Applicable	
Studies involving **human participants:** For publication of **patient photos**, include a statement confirming that consent to publish was obtained.	Not Applicable	
Studies involving experimental **animals**: State details of **authority granting ethics approval** (IRB or equivalent committee(s), provide reference number for approval. Include a statement of compliance with ethical regulations.	Not Applicable	
Studies involving **specimen and field samples:** State if relevant **permits** obtained, provide details of authority approving study; if none were required, explain why.	Not Applicable	

Dual Use Research of Concern (DURC)	Information included in the manuscript?	In which section is the information available? (Reagents and Tools Table, Materials and Methods, Figures, Data Availability Section)
Could your study fall under dual use research restrictions? Please check biosecurity documents and list of select agents and toxins (CDC): https://www.selectagents.gov/sat/list.htm	Not Applicable	
If you used a select agent, is the security level of the lab appropriate and reported in the manuscript?	Not Applicable	
If a study is subject to dual use research of concern regulations, is the name of the **authority granting approval and reference number** for the regulatory approval provided in the manuscript?	Not Applicable	

Reporting

The MDAR framework recommends adoption of discipline-specific guidelines, established and endorsed through community initiatives. Journals have their own policy about requiring specific guidelines and recommendations to complement MDAR.

Adherence to community standards	Information included in the manuscript?	In which section is the information available? (Reagents and Tools Table, Materials and Methods, Figures, Data Availability Section)
State if relevant guidelines or checklists (e.g., **ICMJE, MIBBI, ARRIVE, PRISMA**) have been followed or provided.	Not Applicable	
For **tumor marker prognostic studies,** we recommend that you follow the **REMARK** reporting guidelines (see link list at top right). See author guidelines, under 'Reporting Guidelines'. Please confirm you have followed these guidelines.	Not Applicable	
For **phase II and III randomized controlled trials**, please refer to the **CONSORT** flow diagram (see link list at top right) and submit the CONSORT checklist (see link list at top right) with your submission. See author guidelines, under 'Reporting Guidelines'. Please confirm you have submitted this list.	Not Applicable	

Data Availability

Data availability	Information included in the manuscript?	In which section is the information available? (Reagents and Tools Table, Materials and Methods, Figures, Data Availability Section)
Have **primary datasets** been deposited according to the journal's guidelines (see 'Data Deposition' section) and the respective accession numbers provided in the Data Availability Section?	Yes	Data Availability
Were **human clinical and genomic datasets** deposited in a public access-controlled repository in accordance to ethical obligations to the patients and to the applicable consent agreement?	Not Applicable	
Are **computational models** that are central and integral to a study available without restrictions in a machine-readable form? Were the relevant accession numbers or links provided?	Yes	Data Availability
If publicly available data were reused, provide the respective **data citations in the reference list**.	Yes	Data Availability
